# Plasma levels of hsa‐miR‐3158‐3p microRNA on admission correlate with MRI findings and predict outcome in cerebral malaria

**DOI:** 10.1002/ctm2.396

**Published:** 2021-06-06

**Authors:** Himanshu Gupta, Praveen K. Sahu, Rajyabardhan Pattnaik, Anita Mohanty, Megharay Majhi, Akshaya K. Mohanty, Lukas Pirpamer, Angelika Hoffmann, Sanjib Mohanty, Samuel C. Wassmer

**Affiliations:** ^1^ Department of Infection Biology London School of Hygiene and Tropical Medicine London UK; ^2^ Center for the Study of Complex Malaria in India (CSCMi) Rourkela Odisha India; ^3^ Department of Intensive Care Ispat General Hospital Rourkela Odisha India; ^4^ Department of Radiology Ispat General Hospital Rourkela Odisha India; ^5^ Infectious Diseases Biology Unit Institute of Life Sciences Bhubaneswar Odisha India; ^6^ Department of Neuroradiology University Hospital Heidelberg Heidelberg Germany; ^7^ Department of Neuroradiology Bern University Bern Switzerland

Dear Editor,

We present here a new microRNA (miRNA) biomarker for the prognosis of cerebral malaria (CM) that could be used instead of expensive neuroimaging to monitor disease severity and progression. Severe *Plasmodium falciparum* malaria (SM) remains a leading cause of mortality worldwide and is characterized by a combination of unregulated inflammatory processes and sequestration of infected erythrocytes (IE) within microvessels. This can lead to the dysfunction of one or several vital organs, resulting in a broad clinical picture.^1^ CM is an acute neurologic complication and often fatal form of SM. Prompt diagnosis and treatment are the key for a positive outcome but remain challenging. Recent reports identified age‐specific magnetic resonance imaging (MRI) features on admission associated with CM fatality, namely brain swelling in children and decreased apparent diffusion coefficient (ADC) values indicative of global hypoxia in adults.^2^, ^3^ Because neuroimaging facilities are rarely accessible in malaria‐endemic countries, we investigated the potential of miRNAs, which are rapidly released upon organ damage, as biomarkers of such MRI features across different age groups in a cohort of Indian patients.

We retrospectively analyzed malaria‐positive samples (*n* = 79) from patients admitted to Ispat General Hospital, Rourkela, India, between 2013 and 2019. SM and uncomplicated malaria (UM) patients were defined using the modified WHO criteria (supplementary materials), and European malaria naïve donors (*n* = 32) served as healthy controls (HC). Clinical characteristics of the cohort are presented in Figure S1 and Table S1. TaqMan‐advanced quantitative reverse transcription PCR (RT‐qPCR) assays were used to measure miRNA levels using 50 μl of plasma (supplementary materials).^4^ The selection of miRNAs was based on published reports (supplementary materials; Figure S2). Cycle threshold values were <24 in all samples, with a coefficient of variance < 6% for the exogenous control (cel‐miR‐39‐3p) amplified by RT‐qPCR, indicative of a successful cDNA preparation. PCR efficiencies for all miRNAs had a 91%–106.6% range (Table S2). miRNA levels were compared in patients with CM alone (CM*), CM and another concomitant organ involvement (CM^+^), severe non‐CM (SNCM), UM, and HC. The relative expression levels (RELs) of hsa‐miR‐146a‐5p, hsa‐miR‐150‐5p, hsa‐miR‐222‐3p and hsa‐miR‐3158‐3p were significantly higher in patients with SM (CM, SNCM) compared to UM (*p* < 0.05; Figure 1). These four miRNAs remained associated with malaria severity when compared to both UM and HC or only UM patients (*p* < 0.05; Figures 2 and S3). A total of 1670 putative targets engaged in a wide range of biological and pathological mechanisms were identified for these miRNAs, including brain injury, as well as SM‐associated processes and immune responses (supplementary materials, Tables S3 and S4, Figure S4).

**FIGURE 1:**
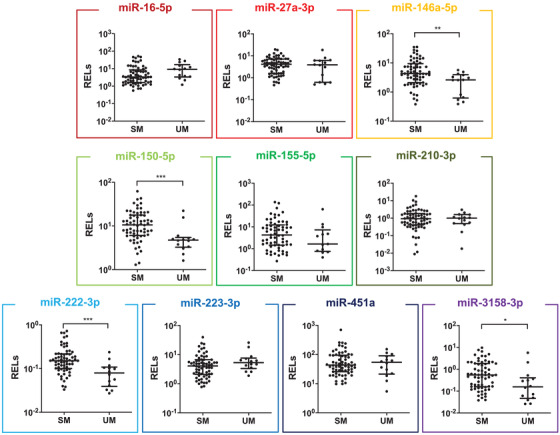
Plasma miRNA levels in SM. Levels of 10 selected miRNAs in plasma samples from Indian patients with SM, including fatal and non‐fatal CM (*n* = 5 and 37, respectively), non‐fatal SNCM (*n* = 23), were compared to samples from UM patients from the same population (*n* = 14). Each miRNA was allocated a color at random to facilitate their identification throughout the manuscript. Relative expression levels (RELs) were calculated with respect to the mean of two endogenous controls (hsa‐miR‐30d‐5p and hsa‐miR‐191‐5p) and compared between patients with different malaria severity groups. Statistical differences were obtained from Mann‐Whitney U test. T bars represent median and interquartile ranges. (*p < 0.05, **p < 0.005, ***p < 0.0005)

**FIGURE 2:**
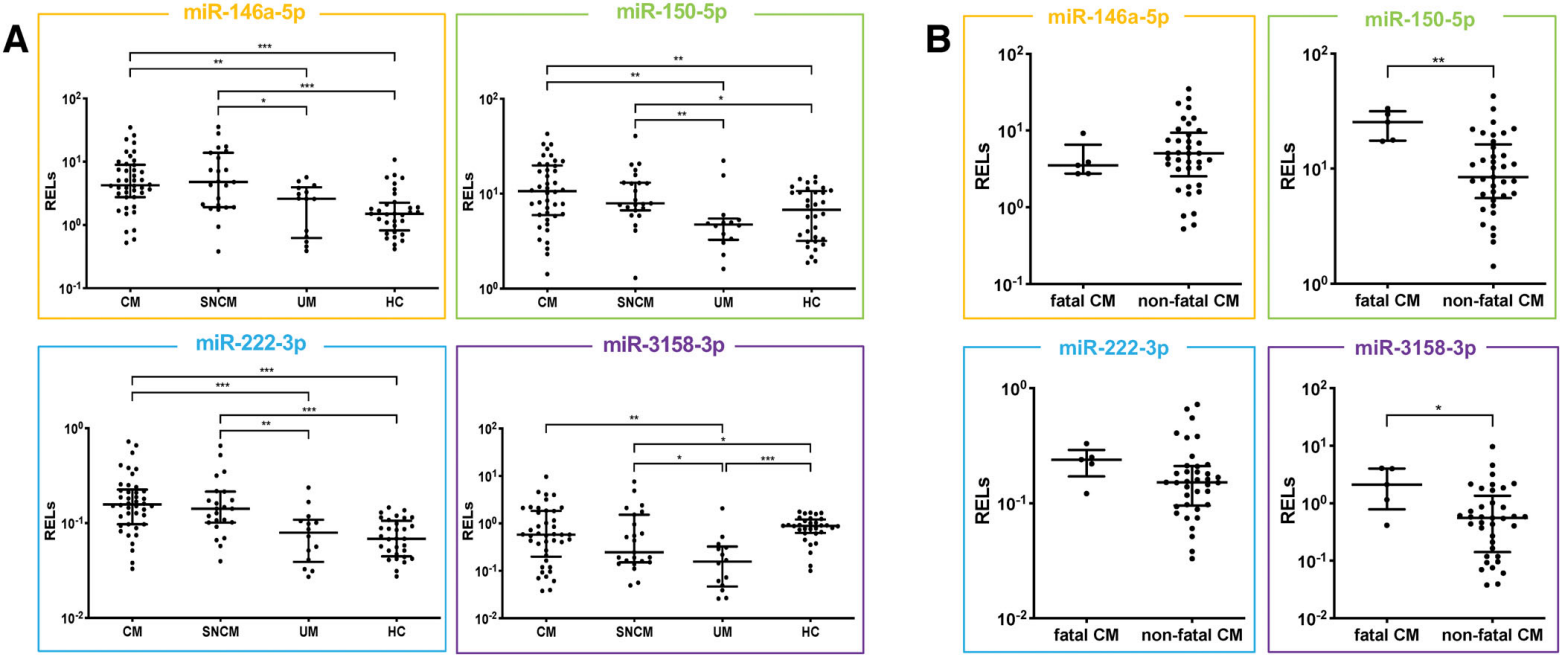
Association of miRNA plasma levels with malaria severity and CM fatality. Relative expression levels (RELs) of hsa‐miR‐146a‐5p, hsa‐miR‐150‐5p, hsa‐miR‐222‐3p, and hsa‐miR‐3158‐3p, all significantly higher in SM patients, were further compared between (A) patients with different malaria severity groups and European healthy controls (HC) as well as (B) patients with fatal CM and non‐fatal CM. RELs were calculated with respect to the mean of two endogenous controls (hsa‐miR‐30d‐5p and hsa‐miR‐191‐5p) and compared. Statistical differences were obtained from Mann‐Whitney U test. T bars represent median and interquartile ranges. (*p < 0.05, **p < 0.005, ***p < 0.0005) Abbreviations: CM, cerebral malaria; UM, uncomplicated malaria; SNCM, severe non‐cerebral malaria.

However, only hsa‐miR‐150‐5p and hsa‐miR‐3158‐3p showed significantly higher plasma levels in fatal CM compared to non‐fatal cases (*p* < 0.05; Figure 2 and S3). These two miRNAs could also discriminate patients with fatal CM from non‐fatal CM, SNCM, and UM cases, as well as HC, with AUC of 78%–100%, sensitivity of 80%–100%, and specificity of 74%–100% (p < 0.05; Table S5). No significant differences were observed when miRNA RELs were compared between patients with non‐fatal CM* and CM^+^ (Figure S5). Of note, both miRNAs were significantly higher in the plasma of fatal CM^+^ patients when compared to survivors (Figure S5). Among the four miRNAs, hsa‐miR‐3158‐3p RELs significantly decreased in plasmas collected at Day 30 compared to Day 0 in CM patients (*p* = 0.031; Figure S6), strongly suggesting its cerebral specificity. All enrolled CM patients underwent MRI scanning as part of a separate project, and imaging datasets were used to generate quantitative brain volume, as well as whole‐brain ADC maps.^2^ Using the Spearman correlation analysis, we show that hsa‐miR‐3158‐3p RELs correlated negatively with brain volume (*r* = ‐0.89; *p* = 0.033) and whole‐brain ADC (*r* = ‐0.47; *p* = 0.043) on admission in children and adults, respectively (Figure 3).

**FIGURE 3:**
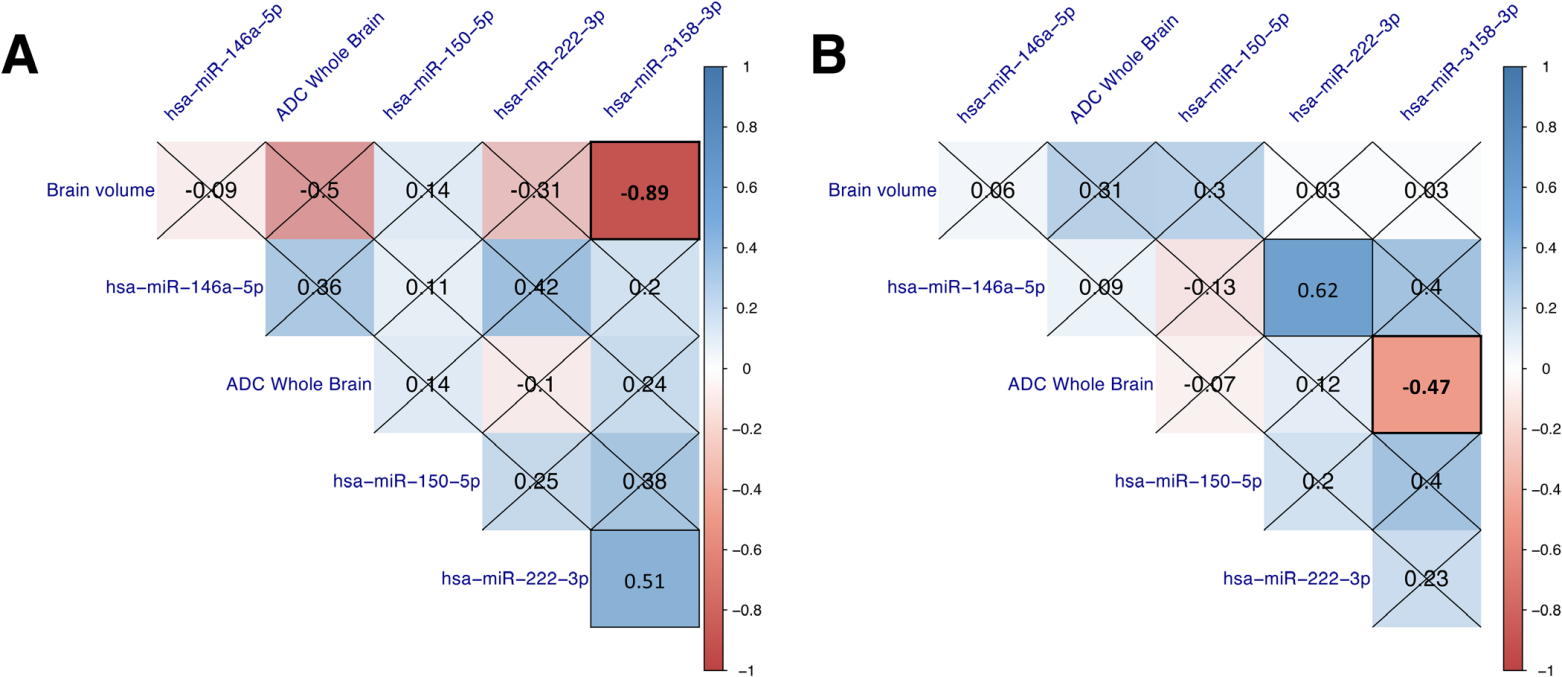
Correlation matrices between miRNA and MRI features in CM patients. Spearman's correlation test was used to assess the correlation between MRI data and miRNAs in children (≤16 years) (A) and adults (>16 years) (B). The color gradient symbolizes the strength of correlation for each comparison, and non‐significant correlations are marked with a cross Abbreviations: ADC, apparent diffusion coefficient.

Previous reports demonstrated that the expression and levels of circulating human miRNAs are highly sensitive to physiological and pathological stimuli.^5^ As a result, their changes in response to infection by *Plasmodium falciparum* raise the possibility of new diagnostic and potentially prognostic tools for SM patients.^6^ Here, we show that hsa‐miR‐3158‐3p levels on admission can both discriminate between SM and UM patients and predict CM fatality. Our results also suggest that hsa‐miR‐3158‐3p could be used as a diagnostic tool for severe/cerebral falciparum malaria across all age groups. In CM patients, we demonstrate for the first time that plasma hsa‐miR‐3158‐3p levels are negatively correlated with brain volume and whole‐brain ADC values. This indicates that the production of hsa‐miR‐3158‐3p is decreased in CM patients with high brain volume on admission, a feature associated with a poor outcome in children.^2^, ^3^ Inversely, hsa‐miR‐3158‐3p levels increased in patients with low global ADC values on admission, a hallmark of fatal adult CM.^2^ Such hypoxia‐specific signal in the brain is consistent with the mechanical obstruction of cerebral microvessels by sequestered IE,^7^ which may result in higher hsa‐miR‐3158‐3p production. Indeed, elevated circulating levels of hsa‐miR‐3158‐3p were reported in patients recovering from ischemic stroke.^8^ Its expression was also found significantly increased in an in vitro model of neurodegenerative disorders in response to oxidative stress.^9^ Hypoxic conditions trigger the production of reactive oxygen species and favor oxidative stress,^10^ potentially further contributing to the high plasma levels of hsa‐miR‐3158‐3p observed in fatal CM patients. The results of our clustering analysis confirmed that some of the gene targets of hsa‐miR‐3158‐3p are involved in hypoxia‐related processes, including HIF‐1 signaling (hsa04066), response to hypoxia (GO:0001666) and cellular response to hypoxia (GO:0071456). Additional validations of the association between brain‐specific hypoxia and the increased production of hsa‐miR‐3158‐3p are needed. Further limitations include our small sample size, which led to the loss of significance for some of our candidates when Benjamini‐Hochberg correction was applied (Table S6), and potential discrepancies in miRNA baselines between European controls and Indian patients. However, this is the first correlation analysis between MRI datasets and miRNA levels in CM patients, which opens new avenues for prognosis in this devastating disease.

In conclusion, hsa‐miR‐3158‐3p represents a promising biomarker candidate for CM prognosis across age groups that may be considered instead of neuroimaging to monitor disease progression and, potentially, inform clinical management in the future by helping the selection of adjunctive treatment targeting brain swelling and/or hypoxic injury.

## CONFLICT OF INTEREST

The authors declare that there is no conflict of interest that could be perceived as prejudicing the impartiality of the research reported.

## ETHICS, CONSENT, AND PERMISSION

Ethical approval was obtained from the Indian Council of Medical Research (TDR589/2010/ECDII), as well as from the institutional review boards from New York University School of Medicine (S12‐03016), the London School of Hygiene and Tropical Medicine, Heidelberg University, and Ispat General Hospital, Rourkela, Odisha, India. A signed informed consent was obtained from all participants and/or their legal guardians. In accordance with the Health Insurance Portability and Accountability Act, patient details were kept confidential using a unique study number.

## AUTHOR CONTRIBUTIONS

Sanjib Mohanty conceptualized the original MRI study of CM pathogenesis at IGH. Himanshu Gupta and Samuel C. Wassmer designed and conceptualized the miRNA analysis. Himanshu Gupta carried out the molecular determinations, bioinformatics, and statistical analyses. Praveen K. Sahu oversaw the recruitment of study patients, their follow‐up scans, and the collection, processing and storage of samples. Rajyabardhan Pattnaik, Anita Mohanty, Megharay Majhi, and Sanjib Mohanty managed patients during their hospitalization and collected the clinical data. Angelika Hoffmann designed the MRI sequence, led the imaging analyses and reviewed all cases. Akshaya K. Mohanty performed clinical laboratory work. Lukas Pirpamer generated the brain volume and ADC data. Himanshu Gupta and Samuel C. Wassmer generated the first draft of the manuscript and all authors contributed to its preparation.

## AVAILABILITY OF DATA AND MATERIALS

Anonymized data are available upon request.

## ROLE OF THE FUNDER/SPONSOR

Funders had no role in the study design, data collection, analysis and interpretation, manuscript preparation, and journal selection for publication. The corresponding authors confirm that they have all the data generated in the study and had final responsibility for the decision to submit for publication.

## Supporting information

Supporting Information
