# Supplementary material for: Plasma levels of hsa‐miR‐3158‐3p microRNA on admission correlate with MRI findings and predict outcome in cerebral malaria
Source: Clin Transl Med. 2021 Jun 6;11(6):e396. doi: 10.1002/ctm2.396 (PMC8181195; doi:10.1002/ctm2.396)
Supplement: Supplementary file 1 — Supporting Information [file CTM2-11-e396-s001.pdf]

SUPPLEMENTARY MATERIALS

Plasma levels of hsa-miR-3158-3p microRNA on admission correlate with MRI findings and predict outcome in cerebral malaria

Appendix List

METHODS 2
FIGURE S1 4
FIGURE S2 5
FIGURE S3 8
FIGURE S4 9
Figure S5 8
Figure S6 9
TABLE S1 10
TABLE S2 11
TABLE S3 12
TABLE S4 24
TABLE S5 57
TABLE S6 58
REFERENCES 58

METHODS

Study site, population, and sampling

This study took place in Rourkela, Odisha, where patients with *P. falciparum* malaria were admitted to Ispat General Hospital, from October 2013 to November 2019. SM and UM patients were defined using the modified WHO criteria [1,2]. SM cases were further categorized as CM and SNCM. None of the individuals enrolled in this study were pre-verbal, and the Glasgow Coma Score (GCS) was used to assess levels of consciousness. CM patients defined as GCS <11 out of 15 after correction of hypoglycemia (<2.2 mmol/l), presented asexual forms of *P. falciparum* in a peripheral blood smear, with or without associated complications such as severe malarial anemia (Hb <7 g/dL for adults and <5 g/dL for children), jaundice (bilirubin >3 mg/dL), acute kidney injury (serum creatinine >3 mg/dL), and hyperlactatemia (lactate >5 mmol/L). SNCM patients were fully conscious (GCS of 15) and had one or several complications described above. Patient characteristics are presented in **Table S1**; they were all treated according to Indian national guidelines. Study exclusion criteria comprised other plasmodial species co-infection, meningitis or other causes of encephalopathy, as well as bacterial infection, as detailed elsewhere [3]. We used consecutive sampling for this study, a non-probability sampling technique that seeks to include all accessible subjects as part of the sample. Because IGH is the only referral hospital for SM in Rourkela and its adjoining districts, this approach ensured that the sampling during the 6 years of enrollment was highly representative of the clinical cases in this locality.

Plasma samples

Whole blood samples (4.5mL, sodium citrate) were collected for all enrolled patients upon admission (day 0), and at follow-up for survivors (day 30 post-admission). 32 archived samples from European malaria-naive adults were used as healthy controls (HC). After centrifugation at 500 *x g* for 10 minutes at 4°C to pellet erythrocytes, platelets and lymphocyte, plasma was collected and frozen at −80°C until batch processing.

miRNAs selection

The selection of miRNAs was based on published reports, miRNAs association with SM and hypoxic conditions [4-11], and miRNAs found involved in mechanisms associated with *P. falciparum* infection [12,13].

Reverse transcriptase quantitative PCR (RT-qPCR)

50µl of plasma samples from the Indian patients and European healthy controls were used for RNA extraction using the miRNeasy Plasma/Serum kit (Qiagen), with the use of 5µg UltraPure™

glycogen/sample (Invitrogen). A synthetic RNA mimicking cel-miR-39-3p was added after lysis reaction as per the manufacturer's instructions. cDNA synthesis and RT-qPCR [ABI 7500 Fast Real-Time System (Applied Biosystems, Foster City, USA)] were performed using the TaqMan® Advanced miRNA assays. A standard curve of five serially diluted points was prepared with cDNA of randomly selected samples and was run in triplicate for each miRNA. Results were normalized using a combination of endogenous controls (ECs; hsa-miR-30d-5p and hsa-miR-191-5p) [9]. miRNA relative expression levels (RELs) were calculated with the $2^{-\Delta Ct}$ method [Ct (miRNA) – Mean Ct (ECs)], considering efficiencies of 100% for all the miRNAs and housekeeping genes [14].

In silico analysis

In silico analysis of identified miRNAs was achieved through three different gene target prediction programs: miRDB (http://mirdb.org/), DIANA-microT-CDS (http://www.microrna.gr/microT-CDS), and TargetScan (http://www.targetscan.org/vert_71/). The gene targets occurred in more than one database were identified using an online tool Venny2.1.0 (https://bioinfogp.cnb.csic.es/tools/venny/) and subjected to gene ontology and pathway enrichment analysis ($p<0.05$) via DAVID 6.8 (https://david.ncifcrf.gov/) using *Homo sapiens* as the reference species.

Statistical analysis

Mann-Whitney U test was used to compare RELs between the two groups. Receiver-operating characteristic (ROC) curves were used to assess the accuracy of miRNA biomarkers to detect fatal cerebral malaria. Area under the ROC curve (AUC) with 95% confidence interval (CI), sensitivity and specificity were also evaluated, to assess the discrimination power of studied miRNAs. Wilcoxon matched pairs signed rank test was used to compare miRNA RELs obtained in plasma samples of CM patients collected at day 0 and day 30. Benjamini-Hochberg correction method was used to adjust the p-values for multiple comparisons. Principal component analysis (PCA) was performed using R package prcomp, and the ggbiplot package was used for visualization. Hierarchical clustering was performed using an online ClustVis tool [15]. Spearman correlation analysis was performed to assess the correlation of miRNA RELs with previously identified features associated with poor outcomes in CM patients including brain swelling and decrease apparent diffusion coefficient (ADC) values. A two-sided $p<0.05$ was considered statistically significant. All statistical analyses were performed using R 4.0.3 in Linux-based system and GraphPad Prism 8.4.3.

SUPPLEMENTAL FIGURES

Figure S1: Consort diagram of the patients enrolled in our study. [SM: Severe malaria; UM: Uncomplicated malaria; CM: Cerebral malaria; SNCM: Severe non-cerebral malaria; CM*: only CM; CM+: CM plus multiple organ failure]

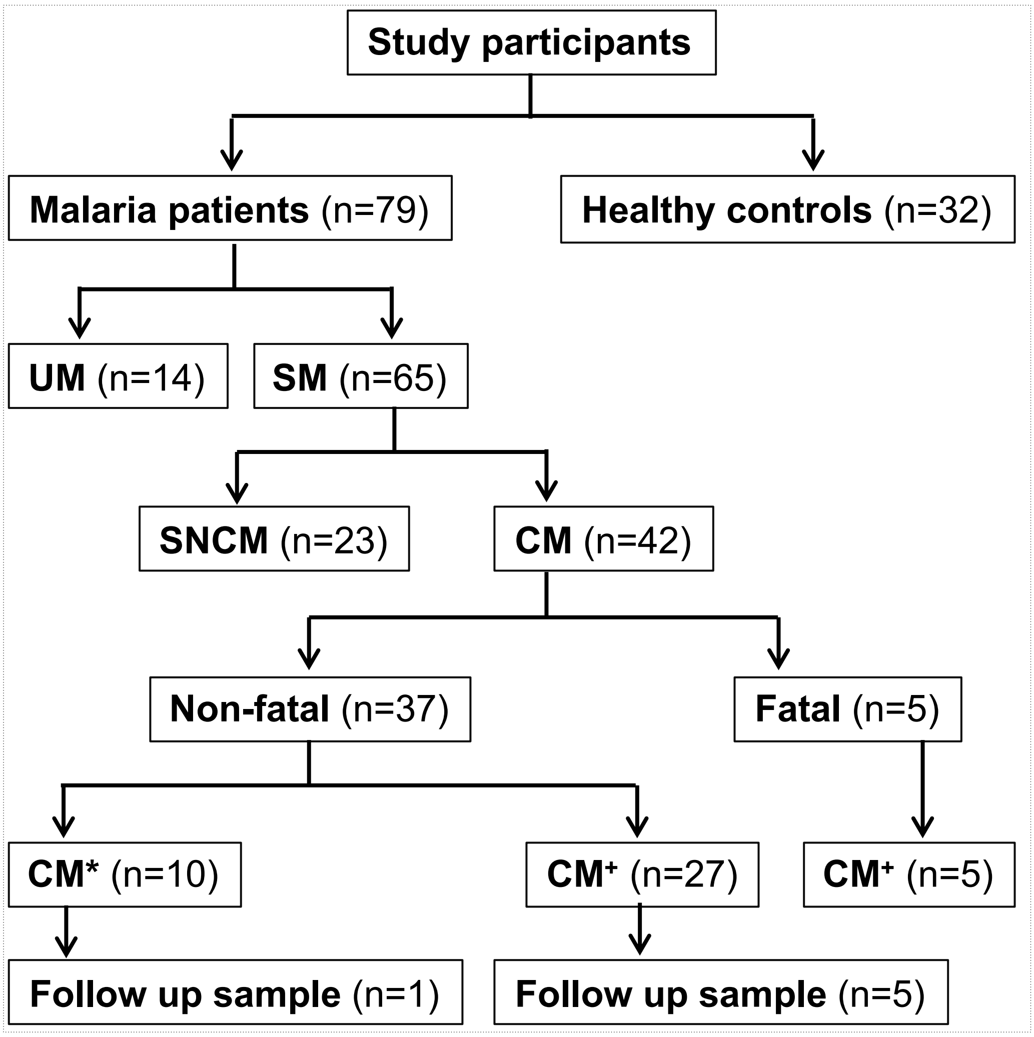

Figure S2: Structure of selected miRNAs. miRBase (http://www.mirbase.org/index.shtml) an online database was used to obtain the structure of the 10 miRNAs selected for this study.

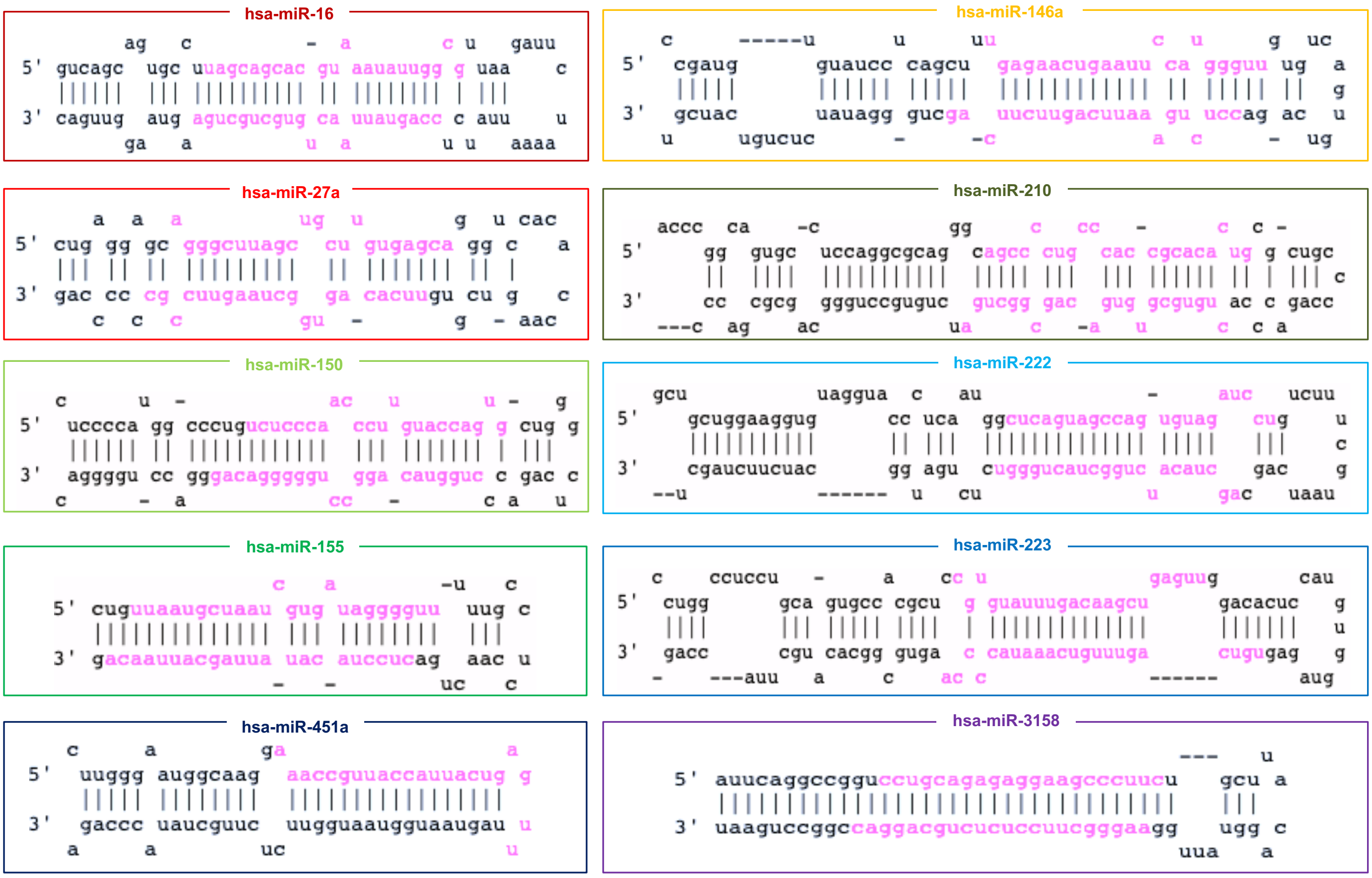

Figure S3: Clustering results: In principal component analysis (PCA), X axis shows principal component 1 and Y axis shows principal component 2 with 54.0% and 21.2% of the total variance, respectively. Relative expression levels of miR-146a-5p, miR-150-5p, miR-222-3p and miR-3158-3p were included in the PCA analysis. Ellipse shapes are showing clustering of different group samples (A). The results of clustering analysis show that miR-150-5p and miR-3158-3p cluster together and the other two miRNAs cluster together (B).

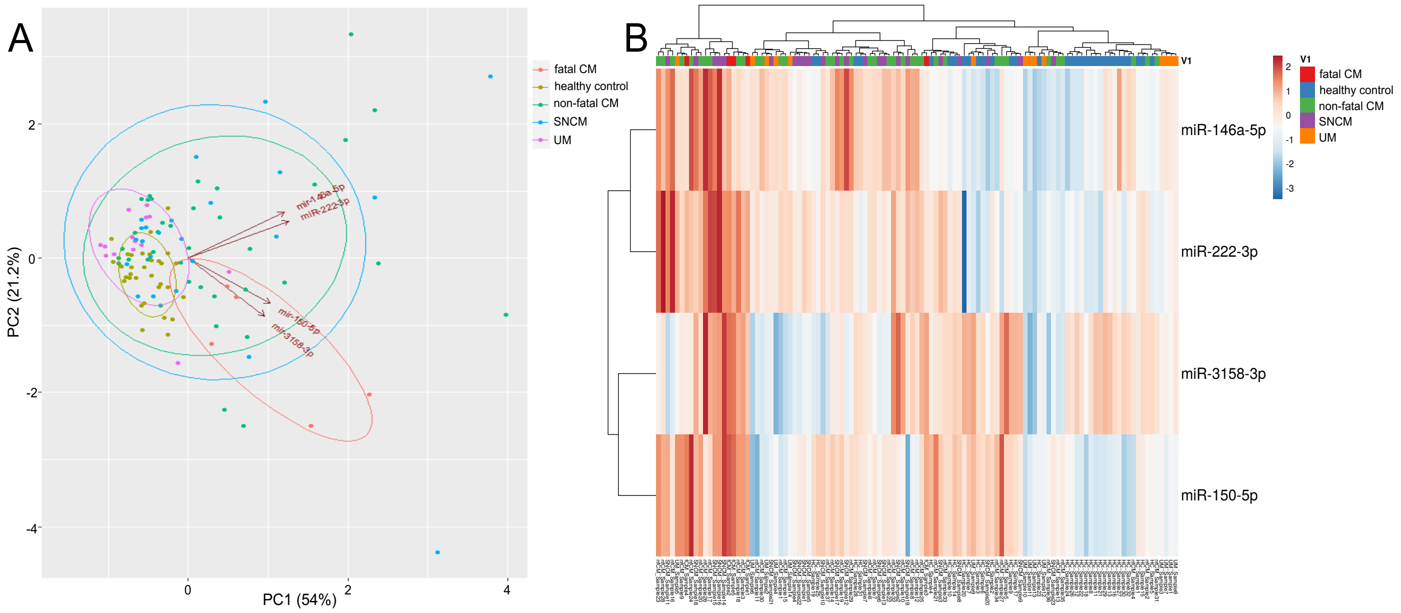

Figure S4: Gene targets of malaria-associated miRNAs. Venn diagram of miRNA gene targets and the numbers indicated in the black boxes represent the miRNA specific gene targets.

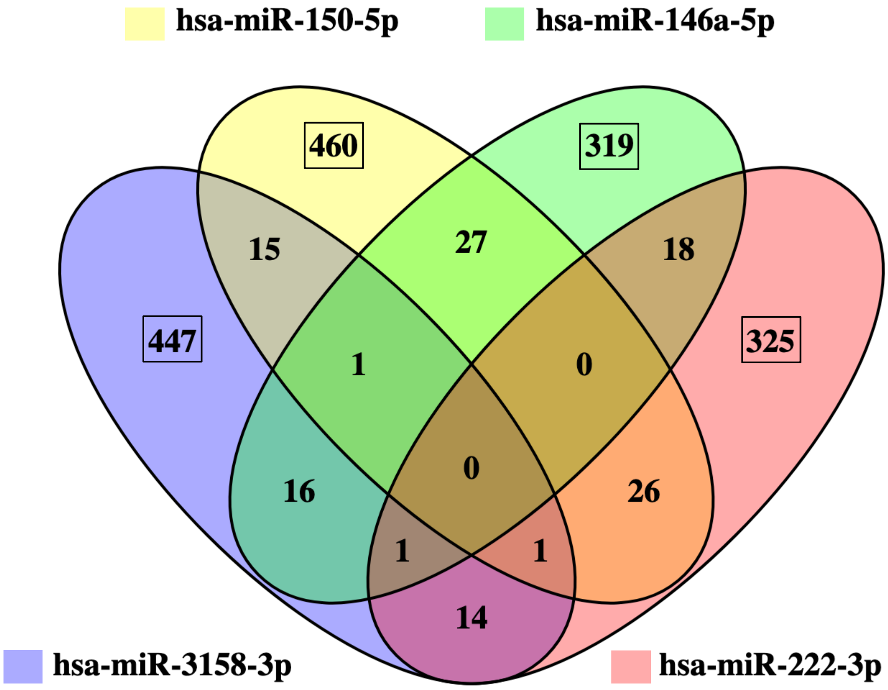

Figure S5: miRNA levels in patients with cerebral malaria only (CM*) compared to patients with cerebral malaria and another concomitant organ involvement (CM+). Relative expression levels (RELs) were calculated with respect to the mean of two endogenous controls (hsa-miR-30d-5p and hsa-miR-191-5p) and compared between CM patients. Statistical differences were obtained from Mann-Whitney U test. T bars represent median and interquartile ranges.

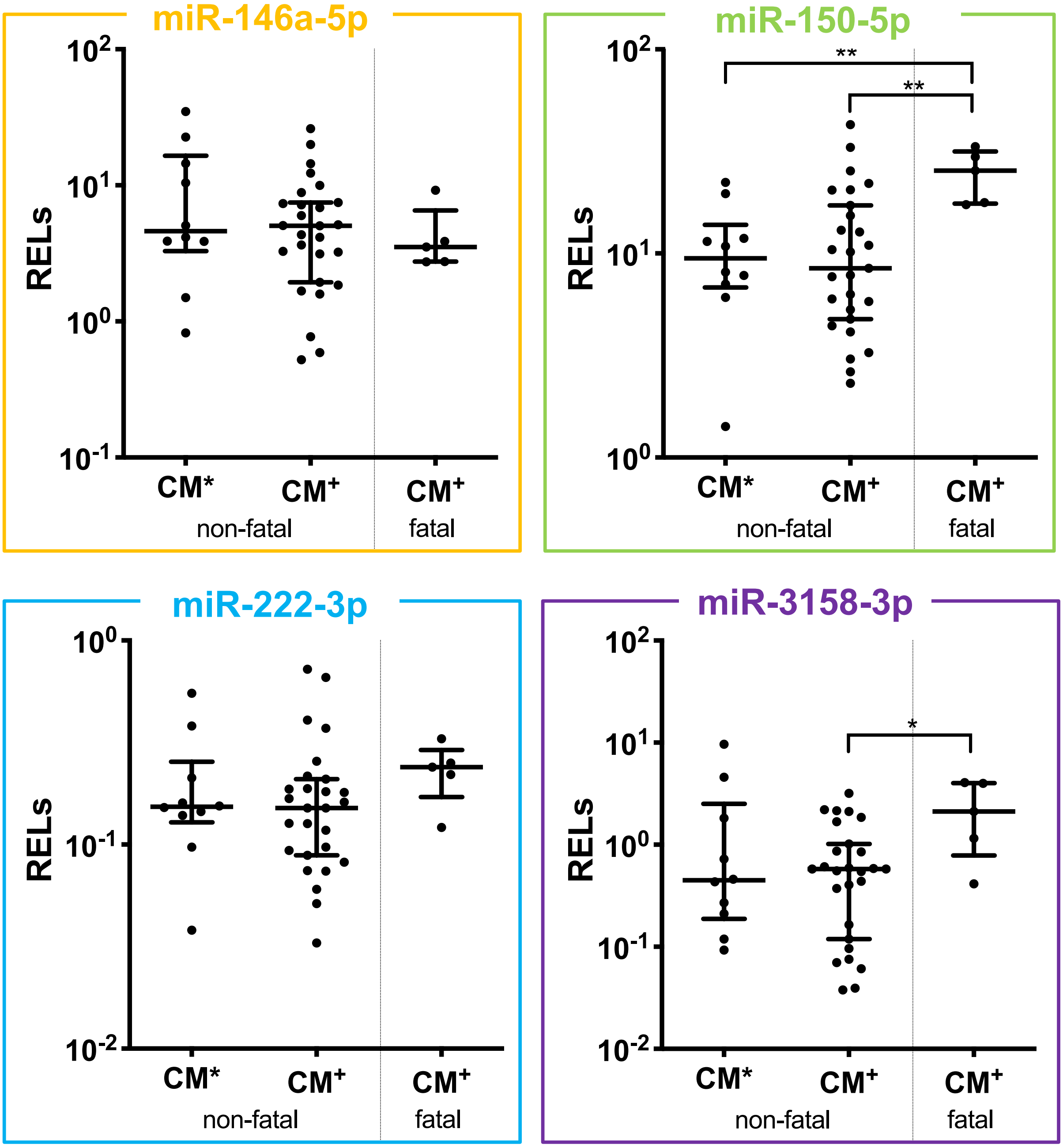

Figure S6: Follow-up matched sample analysis in CM survivors. Relative expression levels (RELs) were calculated with respect to the mean of two endogenous controls (has-miR-30d-5p and has-miR-191-5p) and compared between patient samples collected at admission (Day 0) and post-admission (Day 30). Statistical differences were obtained from Wilcoxon Signed Rank test. [*$p<0.05$, **$p<0.005$, ***$p<0.0005$].

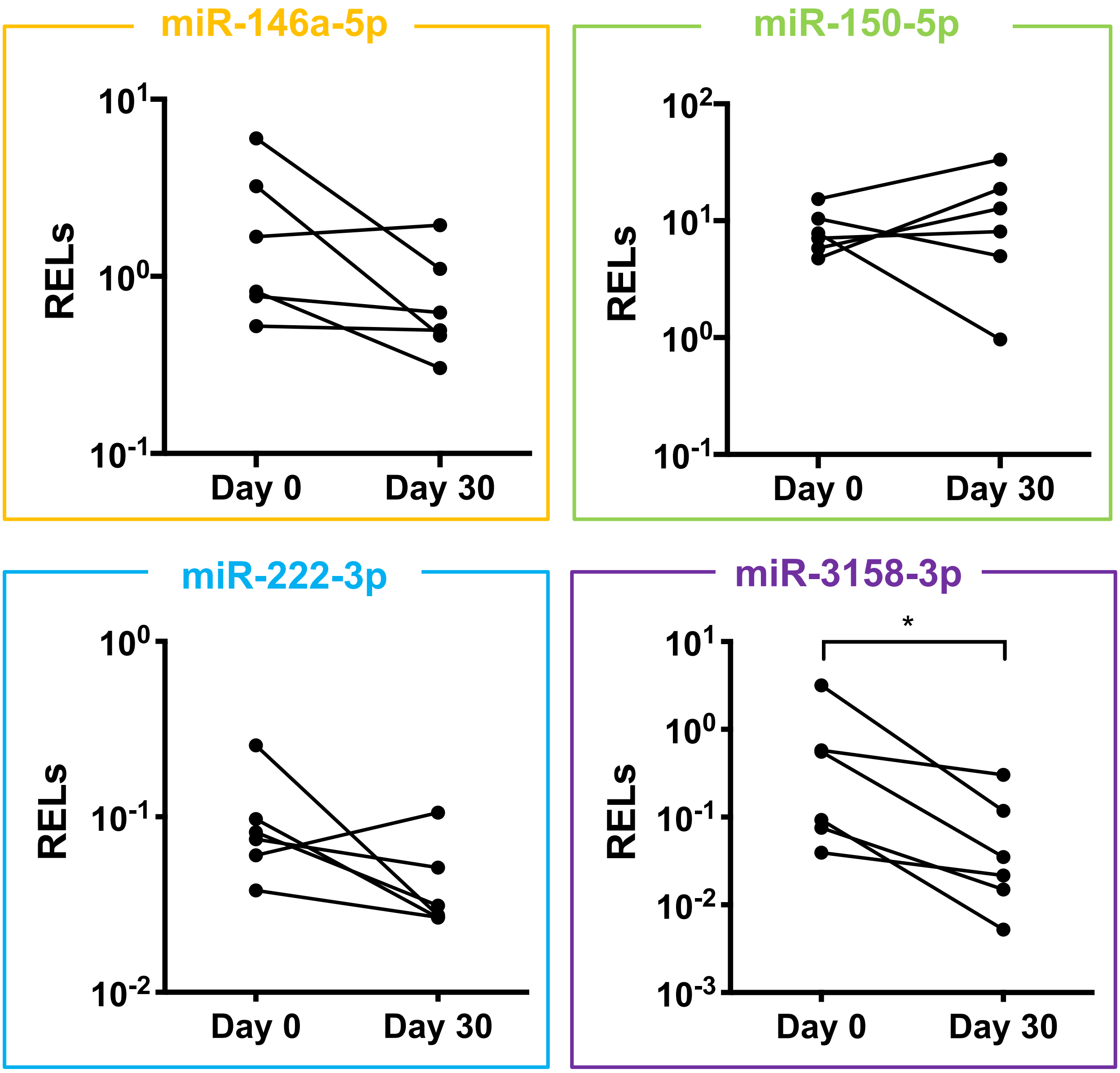

Table S1: Characteristics of Indian patients with severe and uncomplicated malaria recruited from October 2013 to November 2019. Values are expressed in geometric mean±geometric standard deviation factor.

	**fatal CM (n=5)**	**non-fatal CM (n=37)**	**SNCM (n=23)**	**UM (n=14)**
Age, years	16.1±2.9	15.0±2.4	39.5±1.5	35.4±1.6
Sex (n)				
Male	5	22	19	11
Female	0	15	4	3
Parasitemia (/µL)	1922±24.8	5393±19.4	3866±24.3	16409±24.5
[#]Hemoglobin (g/dL)	6.9±1.5	7.1±1.3	7.8±1.5	10.0±1.2
[a]Platelet count (x1000 per cu MM)	33.8±1.9	40.1±2.3	43.4±2.0	49.3±1.7
[ç]Random blood sugar (mg/dL)	136.1±1.5	104.1±1.6	88.0±1.9	123.9±1.2
[^]Bilirubin (mg/dL)	9.7±4.1	3.5±2.7	4.9±2.1	1.3±1.4
[^]Creatinine (mg/dL)	2.9±2.6	1.6±2.8	1.6±2.0	1.0±1.3

CM: cerebral malaria; SNCM: severe non-cerebral malaria; UM: uncomplicated malaria
[#]*No data for 2 sample (non-fatal CM=1; SNCM=1)*
[a]*No data for 5 sample (fatal CM=1; non-fatal CM=2; SNCM=2)*
[ç]*No data for 10 samples (fatal CM=1; non-fatal CM=7; SNCM=1; UM=1)*
[^]*No data for 1 sample (non-fatal CM=1)*

Table S2: PCR efficiencies of each miRNA used for RT-qPCR analysis.

Type	miRNA	PCR efficiencies
Targets	hsa-miR-16-5p	92.70%
	hsa-miR-27a-3p	92.00%
	hsa-miR-146a-5p	93.50%
	hsa-miR-150-5p	98.50%
	hsa-miR-155-5p	91.00%
	hsa-miR-210-3p	94.80%
	hsa-miR-222-3p	97.60%
	hsa-miR-223-3p	97.60%
	hsa-miR-451a	92.60%
	hsa-miR-3158-3p	99.40%
Endogenous controls	hsa-miR-30d-5p	101.00%
	hsa-miR-191-5p	99.90%
Exogenous control	cel-miR-39-3p	106.60%

Table S3: Predicted gene targets of hsa-miR-3158-3p, hsa-miR-150-5p, hsa-miR-146a-5p, and hsa-miR-222-3p, the four miRNAs associated with malaria severity in this study.

hsa-miR-3158-3p	hsa-miR-150-5p	hsa-miR-146a-5p	hsa-miR-222-3p
AC099552.4	C22orf46	ZNF253	ATP1B1
RP3-468K18.5	DNAJB7	CCK	PDIK1L
AC010547.9	ZNF25	SIPA1L1	S100PBP
RGSL1	NCR3LG1	RHOXF2	TP53BP2
C19orf82	PIRT	ZNF506	FBXO28
ZNF506	BORA	UPP2	RAB3GAP2
ZNF30	TDRP	PHKB	MARK1
RFX8	CCDC68	ST7L	LBR
OSBPL1A	LDLR	WDR47	AJAP1
RHOT1	VAPA	PSMF1	PPP1R15B
NUB1	PPP1R3A	ZNF649	CASZ1
ZNF18	SP5	MRS2	GTF2B
USP34	HEATR3	NFAT5	CCDC18
ZNF554	IBA57	DCDC1	ACADM
MROH7	MPZL1	CXXC4	MEX3A
GOLGA1	IRAK2	HMBOX1	CFHR5
CWH43	IFIT5	IER5L	MPZL1
ATP5SL	NRCAM	CCDC89	HIPK1
C9orf156	VEZF1	SEPT14	ZBTB37
CTNNA1	ARHGEF28	MS4A1	WDR77
ZNF333	CHD3	CD96	KCNK2
FGGY	SHC4	C9orf66	DENND1B
ZNF655	KMT2A	RHOXF2B	MIA3
KLK1	ZHX2	NOS1	AIDA
ZNF180	FKBP14	ZNF486	RIMS3
APC	SESTD1	TIMELESS	GNAI3
CD79A	TTI2	BZW1	NBPF3
DNM1L	SYNPO2L	ZBTB22	ARID1A
RP11-447L10.1	GRIN2B	PGK2	ARNT
RBKS	THAP8	RBL1	POGZ
GRIN3A	ZCCHC3	ROR1	WDR47
ZNF773	FAM32A	YES1	STMN1
PPT2-EGFL8	CFLAR	RHOBTB3	ETV3
ADAMTS6	IMPACT	FNBP4	AGO4
HS1BP3	GTF2IRD2B	COPS8	DNAJC6
ANKUB1	IPO5	TXNDC8	TRABD2B
TRIP10	ZNF229	ITCH	PHACTR4
ZNF232	CD84	TRIM5	CYR61
GTPBP1	FADS1	PHF20L1	PRUNE
FAM153B	SORCS3	*CCL5 [16]	H3F3A

COG6
NBPF10
ESPL1
PRIM1
FAM153A
CNOT10
CHRNA10
VPS13B
C14orf79
CCDC132
ST8SIA1
NAMPT
CCNL1
WDR62
ZNF626
TRIML2
CCDC148
GLI2
ZNF317
NRDE2
THOC1
MYBL1
WBSCR22
MTF1
CHKB-CPT1B
DKFZP761J1410
*CR1L [17]
CYP2C8
WT1
SLC44A3
PPM1D
PPWD1
SART3
USP10
ATP2A2
DEDD
PALB2
ZNF35
GORAB
ZFYVE26
PCDH10
C11orf82
MSL3
ITPR3
ZSCAN30
MRPL32

SNAP23
FRAT2
PERP
SV2B
SOWAHC
TMSB4Y
CERS5
ZNF182
PARP11
RASIP1
RBMXL2
ZC3HAV1
GLE1
TNRC6B
POU2F1
CYP4X1
EREG
ZADH2
CCDC170
SLC16A12
LRRC38
SNX30
GLP1R
ZDHHC22
ZNF700
LARP7
GPR26
DTX4
SMC3
MAMDC2
DIO2
OPA3
NUBPL
ZNF335
SLC35B4
IRGQ
R3HCC1L
ZNF773
SLC35A2
SREK1
AKR1D1
CACNG8
ABCB9
DUOX2
FBXL5
EZH1

LANCL1
PI15
ZNF275
RAB8B
TSPAN11
ARL17A
NOX4
TRIM66
FMNL3
PAQR5
THAP5
DCTN5
REEP5
DCAF17
ZNF540
B3GNT5
PRKCE
FAXDC2
LRP2
ONECUT2
ZC3H12B
ZNF493
CPM
ZNF676
EHMT2
ZDHHC7
IQGAP1
TNRC6A
PTAR1
CTAGE4
CTAGE8
ZFAND5
PPBP
VPS54
ZNF662
FZD3
C3orf38
MBNL3
POFUT2
FOXR2
MOB1B
PPM1K
GABRA1
STIL
BCL11A
PDGFRA

KLF7
SOX11
HNRNPA3
MBD5
RAB1A
CCT4
AGPS
SPRED2
AGFG1
ZFP36L2
PPP3R1
CYP1B1
GBX2
ATAD2B
RANBP2
BCL2L11
ERBB4
PKDCC
MAT2A
SPTBN1
EML6
FOXN2
SLC40A1
RFX8
CACNB4
AZI2
KBTBD8
GNAI2
KLHL18
TFG
SEC62
C3orf70
TMCC1
IGF2BP2
MSL2
KPNA1
COL6A5
GTF2E1
ARF4
EIF5A2
PLCL2
MAGI1
ACVR2B
*CD47 [18]
DCUN1D1
PDCD10

CCDC14
TNKS1BP1
GOSR2
AC012123.1
MAFG
PGLYRP4
FAM84B
LDB3
SERINC4
SLC9B2
MATR3
EFCAB4B
PMFBP1
CYB561D1
C9orf72
TRPC3
TMEM165
GALNT16
DERA
FAM159B
ZNF12
FUZ
EIF4G1
EYA1
MAST4
TUBE1
CCDC150
MROH7-TTC4
USP40
GRIK5
DNAJC6
ZKSCAN5
RP13-279N23.2
ZNF155
CKB
GPBP1L1
GOLGA6L1
#NF1[19]
PHIP
STARD7
DYNLRB1
RAB4B-EGLN2
ARHGEF40
MIEF1
RP11-296A16.1
ABCB11
PPARGC1A
SLITRK2
SOGA1
TNRC6A
CEP68
NEUROD6
ALCAM
LTBP2
RNF165
FAM180B
SLC25A30
GOLPH3L
C1orf21
NEMF
GAA
RNASEH1
EIF5
SLC8A1
CTH
KIF1C
ITGAX
MR1
RANBP9
NWD1
ZNF791
ERLIN2
OLIG2
GSG1L
RDH16
ZNF483
ZNF568
SUSD5
C19orf12
PLEKHA1
ZW10
PHF3
NPR3
ATF5
IL13RA1
CXCL14
UBE2J1
MYOM3
ERV3-1
ZC3H14
SAMD14
TBC1D13
GPM6B
TRIM38
PRMT3
MPPE1
BHLHE41
SET
KIAA1211
ATP10B
DTNA
INTS2
INO80D
ALX4
LPP
ZNF737
ADARB1
APOL6
SLC1A1
AUTS2
GIMAP6
FAF2
IYD
OPALIN
SIX4
AMPH
SLITRK4
PRR9
JAG1
CNIH4
ZNF354B
NEB
CNTF
CUL4B
LEPROT
GPX6
IFIT3
FAM98B
PSMB11
TRIP12
SS18
ZNF257
ZNF117
FANCM
ZNF841
CAMTA1
PLSCR4
BCAP29
VGLL4
SNX4
SMARCA5
TRPC3
SEC24B
BEND4
KIT
KDR
SRD5A3
GPM6A
SCD5
SLAIN2
ARHGEF38
STOX2
NDST3
IRF2
ZNF518B
UGT2B15
SNCA
RNF4
MAPK10
NAP1L5
DCUN1D4
SLC4A4
FNIP2
GAB1
TBC1D19
RNF44
SNCB
NIPAL4
MYO10
PANK3
NDFIP1
PAIP2
FAT2
PCDHA2
PCDHA1
PCDHA10
PCDHA5
PCDHA3
PCDHA8
PCDHA12
PCDHAC1
PCDHA13
PCDHA11
PCDHA7

PMS1
TCP10
PPP1R16B
AL645730.2
ZNF23
ZNF629
NEDD4L
FAM118A
RIMS2
TTLL6
TTLL1
DST
TOX3
TTC17
UROS
REPIN1
GSPT1
ZNF544
ITGA4
CAMKK1
AQP2
ABCB10
EVX2
MS4A14
KSR2
KIAA1045
INO80D
MTO1
PICALM
PAX6
RORB
WDR52
ZNF846
MSI2
TMEM229B
ZNF704
VSTM4
MAP3K9
SLC23A2
SEPT4
BPIFC
CPSF6
PCYOX1L
ZFP1
APPL1
HECW2

NBPF3
ZFP30
FAM133B
IRAK1
ZNF331
RNF135
ISLR
TMPPE
ORAI2
ANKRD12
ZNF793
CHRNB2
ZNF514
PIK3R1
ATAT1
NLN
CLMP
AICDA
RBL1
AP1G1
PNPO
GATAD1
EMC1
DNAJC16
RAD54L
PGPEP1
ZNF33A
RND2
MYPN
TSPAN11
VTI1A
ZNF555
SLA
ZFX
CCR2
ITIH5
GLT1D1
PPP2R2A
MEGF9
TTC26
PARD3
TP53INP2
RAB3B
CAMK4
PARD3B
TRNAU1AP

TOX
NPAP1
CEP170B
PRAMEF2
IQCH
MYO1B
KCTD3
TOR1A
FAM120AOS
CREBL2
KCNJ16
CDC37L1
LRRC27
CHST15
SRSF6
CHML
CASP7
ENTPD1
PMAIP1
FBXL22
ZNF138
KRTAP13-1
TET2
ARL8A
ZFP3
TCF20
FUT9
WDFY4
TLN2
MYO1H
FBXL17
ZNF449
ZDHHC17
OR9Q1
CMTM6
ZNF229
AVL9
CTAGE9
EPB41L4A
MED20
TFCP2L1
SLC38A1
KLHL4
C9orf72
DPY19L2
PCYOX1

PCDHA4
PCDHA6
PCDHA9
PCDHAC2
PIK3R1
GABRA1
SH3PXD2B
GDF9
FBN2
TMEM167A
HNRNPA0
PLCXD3
PGGT1B
SKP1
NIPBL
MIER3
LHFPL2
PAIP1
GPBP1
LIFR
ZFYVE16
ANKHD1
LRFN2
SYNCRIP
REV3L
MYLIP
TBC1D22B
OSTM1
ATXN1
RBM24
CNR1
UBE2J1
CLVS2
C6orf120
ESR1
CDK19
KHDRBS2
FRK
USP49
RUNX2
GRM1
SNRNP48
RREB1
ZKSCAN8
GRB10
ADAM22

SOX5
TMOD4
SCCPDH
CLEC4F
GATAD2B
CWC25
OR51E2
C6orf25
XRCC5
SRPK1
AFF2
NCF2
TRMT10A
NUTM2D
FNDC3B
EIF4EBP3
WDFY3
PRDM7
PPIL2
ZSCAN18
MBD1
*IL2 [20]
ZNF341
*#THBS1 [17,21]
RLIM
CDC42EP4
TNFRSF25
CCDC81
TMED7-TICAM2
#SMAD4 [22,23]
SUGT1
ARX
LSM12
CALM2
SMG9
ANKHD1-EIF4EBP3
ZNF550
TMEM38B
PNPLA7
ZIC5
PCDHB4
HNRNPA0
ZNF710
GIPC3
FIGN
FLT3LG

NEK2
CDH7
RAB32
SLC4A4
ABHD12
PDE1C
TBC1D14
LIMD1
GPR155
SAYSD1
ICMT
SIRPB2
ALS2CR12
SPTLC3
CYP8B1
CYTH1
ZMYND8
CREB1
SCRN3
ELK1
RBPMS2
KIAA0319
PCYOX1
CACNB3
CNTLN
GINS1
LARP1
TRMT12
VTA1
DBT
CALML4
ACSL4
TBC1D22B
C1orf198
ASTN2
GOLGA7
PEA15
POF1B
GJC1
ADORA2A
CYP20A1
MTA3
INPP5B
ATAD3B
FAM126A
ALPK1

TMEM19
BRK1
ELAVL1
MARCH6
FAM171A1
SLC9A6
ZNF365
CCDC170
ZNF436
COL9A1
TRMT6
FBXO4
RUNDC3B
GATAD1
WAS
SYT9
MOCS2
NUDT5
RALGPS2
BIVM
MAP3K8
PGM2L1
PSMC6
NPTN
SLC6A15
TRIM2
RACGAP1
KRT6B
ZNF555
TBX18
CACNA2D1
TMEM237
WNT3
C6orf203
PKN2
TMEM185B
FLNA
TRAF6
IRAK1
IGSF1
HIPK3
DCAF12
LCOR
ZBTB2
KLF7
ZNF367

KMT2C
RSBN1L
FKBP9
ANKIB1
NXPH1
INSIG1
CCDC126
RALA
OSBPL3
UBN2
YWHAG
LYPLA1
PCMTD1
MYBL1
SLC25A37
NRG1
TOX
PPP2R2A
RUNX1T1
CHD7
SYBU
ZNF704
TRAM1
KCNQ3
PLEKHA2
TRPS1
HMBOX1
ZFPM2
ANGPTL2
RORB
DMRT3
DCAF12
MEGF9
PPP6C
ZFAND5
PTBP3
LRRC19
RFX3
PHF2
PBX3
RECK
PALM2
LPPR1
KIAA0368
ITIH5
NSMCE4A

TSHZ2	PRKCB	MMP16	HNRNPH3
KRBOX4	TMPRSS11A	SLC10A3	DDIT4
RFT1	METTL7A	*CD80 [24]	FRAT2
TK2	FCRL2	ZDHHC13	HECTD2
IP6K1	HEY2	HNRNPD	CPEB3
DACH1	B3GNT6	FBXW2	RAB18
ZNF257	PDAP1	ERBB4	EMX2
RSF1	FOPNL	EIF4G2	USP6NL
STRBP	CORO2A	RFX7	NANOS1
AMD1	CPSF7	ABL2	CXCL12
FBXO27	DDX51	RARB	TCF7L2
NAA15	RSU1	SIAH2	BMI1
KCNQ5	SCN2B	MYBL1	CAMK1D
ASB15	MGAT3	SEC23IP	ETS1
C1QL3	FAM227A	BCORL1	SESN3
FAN1	USH1C	WWC2	WEE1
DNALI1	*TGFBR1 [25]	NUMB	HIPK3
ATP8A1	SLC7A11	CDKN2AIP	CDON
TSPAN7	CHMP4C	ZNRF3	KIAA1549L
CLIP3	ZNF711	ZNF652	CDKN1C
SLC25A4	ZNF445	C16orf72	GUCY1A2
BRD2	XPNPEP3	ROBO1	DLG2
TMEM178B	ADAM22	STRBP	GALNT18
MCMDC2	PLEKHA2	TMEM120B	CREBZF
#SLC8A3 [26,27]	MAPT	ZNF532	C11orf87
HOXA5	RAB12	NOVA1	CTTN
PPP2R1B	TMEM199	AAK1	KMT2A
TNNI1	MAPK13	ZNF512B	SVIP
SDR42E1	CPM	PPP1R11	SHANK2
TSHZ3	ITPRIPL2	DDHD1	ZNF385A
C10orf53	LPP	APPL1	PDZRN4
C15orf37	MYO5A	LFNG	NAP1L1
ANK3	ARHGEF10L	SYT1	MDM2
CBX6	SLC39A7	PIP5K1B	SYT10
C15ORF37	GJD4	GALNT10	STK38L
MROH9	RNF141	LRRC15	HOXC10
NR6A1	GPATCH8	RIMS2	CDKN1B
ZNF14	PTPRN2	XKR4	APOLD1
OAS2	TEAD1	VASN	CPNE8
IVD	DENND5B	RABGAP1	PLXNC1
GPATCH8	MUC4	CDS1	FRS2
ARIH1	PIGR	ACKR2	MON2
OAS1	NMNAT1	SORT1	NFYB
HTR2A	C2orf72	TBC1D20	ANKRD52
FAM69A	ZNF346	PTGFRN	DNAJC14
PUS3	KSR2	SCN3B	CUX2

ZNF559
FAXDC2
ZNF74
WIPF2
ZNF431
MKX
IL31RA
NF2
TGFBI
POMGNT1
MTRF1
KLF9
KANSL3
RPAP2
SEPT15
TMTC2
#ERBB2 [28,29]
LNX1
CHDH
IGDCC4
MCAT
NHS
RBM4B
#MECP2 [30,31]
POLR1A
CNTNAP2
AHCYL1
ISM2
SYVN1
CLDN1
GPN1
TWSG1
POLH
FAM105B
SOGA2
NFATC3
*CD46 [20]
PYGO2
BCKDHB
STARD4
PDE4C
LIX1
MTF2
MZF1
CEACAM6
SNX27
LRRC27
PSAPL1
SUMO1
DDOST
GALNT1
GALNT6
FUT1
PAFAH2
CCDC69
GIGYF2
PRRG4
RAB3IP
LRRC2
SYNJ2BP
KCTD3
ARRB1
CDKN1B
GRAMD1B
DHX36
SH3TC2
ZFR2
TSPEAR
ST6GAL1
GLYCTK
TMEM108
EHD1
MRPL27
CMKLR1
LRPAP1
AMOTL1
NOS1
PLEKHG4B
MRPL36
TP53INP1
SRSF3
KREMEN1
METTL2A
*MYB [8]
TADA1
ATP8A2
ADIPOR2
FOXD3
ZBTB4
HILPDA
MBTD1
CBL
GDNF
ARMC8
ESYT2
SAMD8
CCDC6
CARD10
FBXO28
RPA3
PRKAA2
ZFYVE1
BMPR1A
MYT1
SRSF12
USP3
SEMA3G
PRX
ZNRF2
GRID1
PTPRA
REL
FAM26E
STC1
TMEM194A
SNX22
CASK
DLGAP2
KIF26B
SLC2A14
MARK1
NRAS
KBTBD4
SMAD4
TMEM200C
AFAP1L2
RPAP2
TANC2
ZBTB26
SYT13
DGKG
SOX5
PM20D2
FMO2
ZBTB8B
VTI1B
ACER3
UBA6
*CD4 [24]
TUBA1A
WSB2
NTF3
CAPRIN2
APAF1
ST8SIA1
KRT81
NAA25
SPPL3
PITPNM2
TMEM132B
RPH3A
IKZF4
SUGT1
ZMYM2
DGKH
CDK8
STK24
FGF14
FNDC3A
ARHGEF7
FRY
SLITRK6
SLITRK5
FOS
BCL11B
AKAP5
RALGAPA1
RGS6
STYX
FERMT2
VASH1
DICER1
KLC1
CHD8
NOVA1
SEMA6D
BMF
TCF12
CHSY1
NGRN
CTDSPL2
EIF3J
FAM214A
DPP8

POLR1D
ADD2
STAT6
EIF3M
CMTR2
ASAP1
RWDD4
NFAT5
MPP7
MMP26
DGKH
CENPBD1
STK40
C11orf48
KANSL1L
NBR1
GFRA2
PDYN
MRPS10
CANX
SUV420H1
L1CAM
HMGA2
#INS [32]
IPO9
CPSF2
XPO5
PPP2R3A
LRRTM4
RAB11FIP4
FAM83F
NIT2
LIMD2
FKBP1A
CCDC18
ZNF778
NFIB
PMP22
PSD2
TSPAN14
TMEM47
EFCAB14
FAM76B
ZSWIM7
ATXN7L3
TAB2

MTCH2
UST
MBD6
USP13
SKP1
ADAM19
POM121C
ELOVL3
PDE7A
NOVA2
ZNF317
PAN2
ETF1
FTO
GDI1
RORB
PAX5
ZEB1
CMTM6
PAPPA
ONECUT2
NFASC
FBXW11
OSBPL9
ZSWIM6
SLC30A5
MDM4
UBFD1
CALCR
MDGA1
PDCD4
NDC1
USP49
RAB11A
LRRC58
GABRA4
SZRD1
NR2F2
ZFP91
GAN
IPO9
NKX2-4
ITIH6
MLXIP
GRHL2
GABRG2

SLC19A3
FAM83F
CLCN6
C16orf52
ZNF148
KPNA6
QKI
C21orf33
RFTN2
GNL1
AC012215.1
CELF3
SH3GL2
NRP2
SBSPON
GABPA
DUSP16
FAM169A
SLCO3A1
RP11-156E8.1
RCAN1
ZNF37A
ST5
SLC39A1
PNKP
ATG7
CDC14A
TM9SF2
IST1
TNIK
BAG1
MYO5A
PIK3CB
UBE2W
SMARCA5
PDZD8
DNAJB14
SLITRK3
NUDT17
SCN1A
TTL
TDRKH
FLOT2
MED1
FZD1
FAM210A

*THBS1 [17]
TLE3
RFX7
MESDC1
ZFHX3
IRX5
NFATC3
ZNF629
CBFB
SBK1
STX1B
BEAN1
RNPS1
POLR3E
ZFP90
CTCF
CMTM4
DYNC1LI2
KIAA0430
C16orf45
FBXO47
DCAF7
TNRC6C
ZNF652
SOCS3
AXIN2
KPNA2
SLC16A6
ITGB3
ASPA
VEZF1
KSR1
MED1
HEXIM1
NLK
MPRIP
NUFIP2
TAOK1
KDSR
MBD2
PIEZO2
SERPINB2
C18orf25
CDH2
ZNF181
BBC3

PRC1
SPOCD1
ELAVL1
ST8SIA2
ARL4C
HIC2
RRAS2
GBE1
SLC25A21-AS1
#ATM [33,34]
AC011897.1
DIO2
LCLAT1
MED29
PPCS
ZNF280B
TMEM150C
FAM9C
C10orf2
MBTD1
DRGX
ABHD2
ALKBH1
ZNF619
USP11
FBXO46
KCNB1
C9orf171
DLG4
CREM
RELN
GRIN2B
RFX3
ONECUT3
SHC1
KCNQ4
CACNA2D4
ZNF362
LRRC15
VEZT
NUTM2F
IFFO2
ZNRF3
FAM13A
RNF144A
LRCH3

PKHD1
CDK13
PLP2
DYRK1A
BASP1
CAMK2G
WDR5B
PIK3AP1
STX5
AIFM2
EGR2
SGMS1
KCNB1
EIF4B
NR1D2
PKP4
TEK
TSPYL5
ENTPD1
MPP6
ZMAT2
IGF2BP1
RC3H1
SRGAP3
CHD2
TP53
FOXO4
DENND4A
FNDC5
SP1
VPS53
MARCH6
ITGB3
TRIM66
EIF4E
RAD23B
RPP14
CERS3
MYOCD
SHISA4
STYXL1
CPD
SMARCD1
MMP14
MMP16
PTGFR

PRCP
NSD1
KDM2B
BTG2
C17orf78
USP47
NUCKS1
ADAM19
ERLEC1
RUNX1T1
LRRC8B
TAF9B
SLC2A3
KIF24
RNF4
GOSR1
JAZF1
TMEM216
PHOX2B
SGIP1

URI1
MIDN
ZNF615
NFATC2
VAPB
KIF16B
DYRK1A
ETS2
BRWD1
SOX10
SNAP29
CRKL
SUN2
BRWD3
AMMECR1
FMR1
WNK3
CLIC2
TMSB15B
FAM199X
NRK
NAP1L2
TSC22D3

ANAPC7	GOSR1
FKBP1C	TET3
SH3PXD2A	FAM49A
SFPQ	FOXP1
ZNF24	PRICKLE2
GLTSCR1L	PRRT2
IKZF3	UPF1
TCTE1	CYB561D1
NFIX	METAP1
DNAJC15	KCTD9
FNDC5	STX3
HMGCL	BSN
SYT15	PLXNC1
PEX26	TRPS1
PARM1	ST3GAL6
RNF150	EBF3
RPS23	JPH2
LRP6	KIAA0087
PRKRA	DGKI
HRK	AGO3
GPX3	CSNK1A1
CHD1L	PALD1
ANKS6	ACO1
ZNF207	FAM179B
LUZP1	GLIS3
TMEM53	ABI2
TRIM51	CNNM2
CD248	COL4A4
*#NFKB1 [35-37]	FDXR
HR	AZIN1
PLCXD3	AC027763.2
ANKRD65	VSIG10
CASP14	GSK3B
NPTXR	PIGG
PPIP5K2	PSMD11
GJD2	PEG10
CACNA1B	CNPPD1
RTKN2	DYNLT1
C18orf63	RASGEF1A
GRPEL1	RSBN1L
PHLDA1	DST
NKPD1	SCP2
TMED7	SOGA3
CNTN2	NEGR1
ACADSB	PAPD5
ADCYAP1R1	BCL9L

EXT1	LMO4
ATG9B	FAM117B
HFM1	CCNT1
ARID3B	CLCN6
EFNA4	ZCCHC17
ZNF420	SH3BP5L
POU2F1	UBR1
CPNE4	MAP1B
SNN	RSPH10B
TAF1B	WNT2B
SMARCD1	CXorf23
ADRA2A	AEBP2
NFYA	KCNN3
ZNF512	RIMKLA
C5orf51	TPM3
USP21	H1F0
SH2D1B	NEFM
PREPL	TLR5
ASB18	UBE2R2
ALDH3B2	IRF2BP2
POLDIP2	PIK3CB
PVRL4	PTBP2
PPARG	ANKRD52
UBE2F	TMCC1
ITSN1	SEC31B
SLA2	KLHL3
SGCD	EXO5
SNX24	AKT3
HADHA	VMP1
MED13L	BTBD9
ATXN3	ACTRT3
TRIM32	APC
SZT2	CELF3
FOXN3	DCAF6
MAX	WTAP
TXNIP	EPHB2
HEMK1	PRKCA
IL6ST	KCNIP1
ADAMTS14	HSP90B1
TM2D3	GGNBP2
CACNA2D1	C6orf120
	PRKAR1A
	ENSA
	HNRNPH3
	PURA
	PAFAH1B1

PDIA6
ZBTB7A
EP300
ZNF865
GRIPAP1
SUV39H2
E2F3
CACNA1G
ZNF189
SBF1
GCM2
ACTR1A
TESK2
RIMS2
ADARB1
UBE2E3
CAST
DUSP13
ZBTB20
IGFBPL1
FZD4
RAD51
PTP4A1
AKIRIN1
CAPZB
PA2G4
PRR3
CCND2
GPAM
*HIF1A [38,39]

*gene targets involved in brain injury and processes relevant to severe malaria, with associated reference.
#gene targets involved in hypoxia related processes, including hypoxia-inducible factor (HIF)-1 signaling (hsa04066), response to hypoxia (GO:0001666) and cellular response to hypoxia (GO:0071456).

Table S4: DAVID clustering analysis results for hsa-miR-3158-3p, hsa-miR-150-5p, hsa-miR-146a-5p, and hsa-miR-222-3p.

Category	Term	Genes	Fold Enrichment	p value	adjusted p value**
KEGG_PATHWAY	hsa04310:Wnt signaling pathway	CAMK2G, PPP3R1, CXXC4, TCF7L2, CHD8, WNT3, FRAT2, AXIN2, NFATC2, FBXW11, NFATC3, APC, CSNK1A1, PRKCA, NLK, TP53, FZD1, SMAD4, FZD3, SKP1, MAPK10, FZD4, PRKCB, WNT2B, EP300, CCND2, GSK3B, PRICKLE2, LRP6	2.590657628	4.04E-06	0.00107526
KEGG_PATHWAY	hsa04012:ErbB signaling pathway	PRKCA, ERBB4, PIK3CB, ERBB2, CAMK2G, CBL, ELK1, MAPK10, PRKCB, NRAS, CRKL, CDKN1B, EREG, GSK3B, GAB1, SHC1, NRG1, ABL2, PIK3R1, AKT3, SHC4	2.975713756	1.43E-05	0.00190373
KEGG_PATHWAY	hsa05205:Proteoglycans in cancer	ERBB4, CAMK2G, ERBB2, ELK1, ITGB3, PDCD4, IQGAP1, CTTN, WNT3, ANK3, GAB1, THBS1, FRS2, AKT3, PIK3R1, PRKCA, PIK3CB, CBL, TP53, FZD1, ESR1, FZD3, ITPR3, FZD4, FLNA, KDR, PRKCB, WNT2B, EIF4B, NRAS, HIF1A, MAPK13, RRAS2, MDM2	2.095752688	5.60E-05	0.00433197
KEGG_PATHWAY	hsa04390:Hippo signaling pathway	PARD3, MOB1B, GLI2, TCF7L2, WNT3, DLG4, LIMD1, AXIN2, FBXW11, DLG2, APC, PPP2R1B, NF2, TP53BP2, TGFBR1, FZD1, SMAD4, TEAD1, FZD3, CTNNA1, FZD4, WNT2B, YWHAG, CCND2, BBC3, GSK3B, BMPR1A, PPP2R2A	2.285978779	6.51E-05	0.00433197
KEGG_PATHWAY	hsa05166:HTLV-I infection	E2F3, TLN2, CREM, PPP3R1, ELK1, NFKB1, NFYB, CANX, FOS, WNT3, NFATC2, MYB, NFATC3, AKT3, PIK3R1, APC, EGR2, SLC25A4, PIK3CB, CREB1, TGFBR1, TP53, FZD1, SMAD4, FZD3, FZD4, ATM, WNT2B, NRAS, EP300, CCND2, ETS1, RRAS2, GSK3B, ETS2, PDGFRA, ANAPC7, TP53INP1, IL2	1.892875286	1.38E-04	0.00736363
KEGG_PATHWAY	hsa05200:Pathways in cancer	E2F3, FGF14, PPARG, NFKB1, GLI2, CXCL12, FLT3LG, FOS, MAX, WNT3, RALA, RARB, AKT3, PRKCA, PIK3CB, TP53, RUNX1T1, CTNNA1, PRKCB, RAD51, CCDC6, CRKL, EP300, HIF1A, PDGFRA, MDM2, GNAI3, GNAI2, ERBB2, TFG, KIT, TCF7L2, TPM3, ARNT, TRAF6, AXIN2, PIK3R1, APC, COL4A4, TGFBR1, CBL, SMAD4, FZD1, FZD3, MAPK10, APPL1, FZD4, WNT2B, NRAS, HSP90B1, CDKN1B, ETS1, GSK3B	1.662548907	2.28E-04	0.01008774
KEGG_PATHWAY	*hsa04010:MAPK signaling pathway [40]	FGF14, PPP3R1, ELK1, NFKB1, CACNB3, CACNB4, FOS, MAX, MAPT, MAP3K8, DUSP16, TRAF6, NFATC3, AKT3, PRKCA, CACNA2D1, NTF3, TAOK1, CACNG8, NLK, TGFBR1, NF1, TP53, MAPK10, TAB2, FLNA, PRKCB, CACNA2D4, NRAS, CRKL, ARRB1, MAPK13, RRAS2, CACNA1G, PDGFRA, STMN1, CACNA1B	1.802902801	5.55E-04	0.01864797
KEGG_PATHWAY	hsa04722:Neurotrophin signaling pathway	IRAK2, IRAK1, NTF3, PIK3CB, CAMK2G, TP53, NFKB1, MAPK10, NRAS, CRKL, CAMK4, MAPK13, GSK3B, GAB1, SORT1, SHC1, TRAF6, FRS2, PIK3R1, AKT3, CALM2, SHC4	2.260125448	5.61E-04	0.01864797
KEGG_PATHWAY	hsa04917:Prolactin signaling pathway	PIK3CB, SOCS3, ESR1, NFKB1, MAPK10, NRAS, FOS, MAPK13, CCND2, INS, GSK3B, SHC1, AKT3, PIK3R1, SHC4	2.604497955	0.00141259	0.03969418

KEGG_PATHWAY	hsa05220:Chronic myeloid leukemia	E2F3, PIK3CB, TGFBR1, CBL, TP53, SMAD4, NFKB1, NRAS, CRKL, CDKN1B, MDM2, SHC1, AKT3, PIK3R1, SHC4	2.568324373	0.00162659	0.03969418
KEGG_PATHWAY	hsa05215:Prostate cancer	E2F3, PIK3CB, CREB1, ERBB2, TP53, NFKB1, TCF7L2, NRAS, HSP90B1, EP300, CDKN1B, INS, GSK3B, PDGFRA, MDM2, AKT3, PIK3R1	2.381537146	0.00164149	0.03969418
KEGG_PATHWAY	hsa05214:Glioma	PRKCA, E2F3, PIK3CB, CAMK2G, TP53, PRKCB, NRAS, PDGFRA, MDM2, SHC1, AKT3, PIK3R1, CALM2, SHC4	2.655252275	0.00180947	0.04010983
KEGG_PATHWAY	hsa04921:Oxytocin signaling pathway	PRKCA, CACNA2D1, GNAI3, GNAI2, CACNG8, CAMK2G, PPP3R1, ELK1, RCAN1, CACNB3, CACNB4, ITPR3, CAMKK1, PRKCB, CACNA2D4, FOS, NRAS, CAMK4, GUCY1A2, PRKAA2, NFATC2, NFATC3, CALM2, CAMK1D	1.972473118	0.00203748	0.04169
KEGG_PATHWAY	hsa05213:Endometrial cancer	NRAS, PIK3CB, GSK3B, ERBB2, TP53, ELK1, AXIN2, CTNNA1, TCF7L2, AKT3, PIK3R1, APC	2.844913151	0.00251073	0.04770379
KEGG_PATHWAY	hsa05231:Choline metabolism in cancer	PRKCA, SLC44A3, PIK3CB, PIP5K1B, LYPLA1, DGKH, MAPK10, DGKI, WAS, PRKCB, NRAS, FOS, HIF1A, SP1, DGKG, PDGFRA, AKT3, PIK3R1	2.197061642	0.00282254	0.05005298
KEGG_PATHWAY	hsa04071:Sphingolipid signaling pathway	PPP2R1B, PRKCA, PPP2R3A, GNAI3, GNAI2, PIK3CB, SPTLC3, TP53, CERS5, NFKB1, MAPK10, SGMS1, CERS3, PRKCE, PRKCB, NRAS, MAPK13, PIK3R1, AKT3, PPP2R2A	2.054659498	0.00336403	0.05275067
KEGG_PATHWAY	*hsa04910:Insulin signaling pathway [41]	PHKB, PIK3CB, SOCS3, FLOT2, CBL, ELK1, MAPK10, PPP1R3A, PPARGC1A, NRAS, CRKL, EIF4E, INS, GSK3B, PRKAR1A, SHC1, PRKAA2, TRIP10, CALM2, AKT3, PIK3R1, SHC4	1.965326477	0.00344	0.05275067
KEGG_PATHWAY	hsa05210:Colorectal cancer	PIK3CB, TGFBR1, SMAD4, TP53, MAPK10, APPL1, TCF7L2, FOS, GSK3B, AXIN2, AKT3, PIK3R1, APC	2.584894207	0.00356959	0.05275067
KEGG_PATHWAY	hsa04014:Ras signaling pathway	FGF14, ELK1, NFKB1, KIT, FOXO4, GRIN2B, REL, INS, GAB1, TEK, RALA, SHC1, PIK3R1, AKT3, SHC4, PRKCA, PIK3CB, NF1, MAPK10, KDR, PRKCB, NRAS, KSR2, ETS1, RRAS2, ETS2, PDGFRA, EFNA4, KSR1, ABL2, CALM2	1.69100295	0.00457445	0.06166405
KEGG_PATHWAY	hsa04130:SNARE interactions in vesicular transport	SNAP29, STX5, STX3, VTI1B, GOSR2, GOSR1, SNAP23, STX1B, VTI1A	3.263282732	0.00482695	0.06166405
KEGG_PATHWAY	hsa05206:MicroRNAs in cancer	BMI1, E2F3, CYP1B1, ERBB2, DICER1, IGF2BP1, NFKB1, ITGB3, ZEB1, PDCD4, WNT3, SHC1, THBS1, BMF, SHC4, APC, RECK, PRKCA, TP53, MMP16, FZD3, PRKCE, HMGA2, ATM, BCL2L11, PRKCB, DDIT4, NRAS, CRKL, CDKN1B, EP300, CCND2, PDGFRA, MDM2, ZFPM2, STMN1, MDM4	1.594875555	0.00486821	0.06166405
KEGG_PATHWAY	*hsa04668:TNF signaling pathway [40]	CFLAR, DNM1L, PIK3CB, SOCS3, CREB1, NFKB1, JAG1, MAPK10, CCL5, MMP14, TAB2, FOS, MAPK13, CASP7, MAP3K8, ITCH, PIK3R1, AKT3	2.073861923	0.0052047	0.06292954
KEGG_PATHWAY	hsa04916:Melanogenesis	PRKCA, GNAI3, GNAI2, CREB1, CAMK2G, FZD1, FZD3, KIT, FZD4, TCF7L2, PRKCB, WNT2B, NRAS, WNT3, EP300, GSK3B, CALM2	2.095752688	0.0061711	0.0685639
KEGG_PATHWAY	hsa05161:Hepatitis B	PRKCA, E2F3, EGR2, PIK3CB, CREB1, TGFBR1, TP53, SMAD4, ELK1, NFKB1, MAPK10, PRKCB, STAT6, FOS, NRAS, EP300, CDKN1B, APAF1, NFATC2, NFATC3, PIK3R1, AKT3	1.870448647	0.00618622	0.0685639

KEGG_PATHWAY	hsa04120:Ubiquitin mediated proteolysis	SYVN1, SOCS3, PPIL2, CBL, UBE2J1, UBA6, UBE2F, SKP1, UBE2R2, UBE2E3, TRIM32, MDM2, UBE2W, FBXO4, NEDD4L, ANAPC7, ITCH, CUL4B, TRAF6, FBXW11, TRIP12	1.889686838	0.00681569	0.07251893
KEGG_PATHWAY	hsa04725:Cholinergic synapse	PRKCA, GNAI3, GNAI2, PIK3CB, CREB1, CAMK2G, ITPR3, PRKCB, KCNQ5, NRAS, FOS, KCNQ4, KCNQ3, CAMK4, CHRNB2, PIK3R1, AKT3, CACNA1B	1.99912816	0.00755869	0.07733123
KEGG_PATHWAY	hsa05202:Transcriptional misregulation in cancer	BMI1, KMT2A, CCNT1, PPARG, TP53, RUNX1T1, TSPAN7, PAX5, NFKB1, SIX4, HMGA2, ATM, WT1, SS18, MAX, EYA1, CDKN1B, SP1, REL, CCND2, MDM2, H3F3A, PBX3, RUNX2	1.771682442	0.00792637	0.07808945
KEGG_PATHWAY	hsa04550:Signaling pathways regulating pluripotency of stem cells	BMI1, PIK3CB, IL6ST, SMAD4, PAX6, FZD1, LIFR, FZD3, FZD4, WNT2B, NRAS, ACVR2B, WNT3, MAPK13, GSK3B, AXIN2, ZFHX3, PIK3R1, AKT3, BMPR1A, APC	1.849193548	0.00864946	0.08216988
KEGG_PATHWAY	hsa05142:Chagas disease (American trypanosomiasis)	PPP2R1B, IRAK1, CFLAR, GNAI3, GNAI2, PIK3CB, TGFBR1, NFKB1, MAPK10, CCL5, FOS, MAPK13, TRAF6, AKT3, PIK3R1, IL2, PPP2R2A	2.015146816	0.0090089	0.08263332
KEGG_PATHWAY	hsa05217:Basal cell carcinoma	WNT3, GSK3B, TP53, FZD1, FZD3, GLI2, AXIN2, FZD4, TCF7L2, APC, WNT2B	2.511250498	0.01033324	0.08897196
KEGG_PATHWAY	hsa04015:Rap1 signaling pathway	PARD3, GNAI3, GNAI2, FGF14, ADORA2A, TLN2, KIT, ITGB3, GRIN2B, INS, CNR1, TEK, RALA, THBS1, PIK3R1, AKT3, PRKCA, MAGI1, PIK3CB, KDR, PRKCB, NRAS, CRKL, MAPK13, SIPA1L1, PDGFRA, EFNA4, CALM2	1.643727599	0.01052932	0.08897196
KEGG_PATHWAY	hsa04919:Thyroid hormone signaling pathway	PRKCA, ATP1B1, PIK3CB, TP53, ESR1, RCAN1, ITGB3, MED13L, PRKCB, NRAS, EP300, HIF1A, DIO2, GSK3B, MDM2, AKT3, PIK3R1, MED1	1.929593268	0.01070339	0.08897196
KEGG_PATHWAY	hsa04915:Estrogen signaling pathway	GNAI3, GNAI2, PIK3CB, CREB1, ESR1, ITPR3, GRM1, NRAS, FOS, HSP90B1, SP1, SHC1, CALM2, AKT3, PIK3R1, SHC4	1.992397089	0.01287217	0.10375749
KEGG_PATHWAY	hsa05212:Pancreatic cancer	E2F3, PIK3CB, TGFBR1, ERBB2, TP53, SMAD4, RALA, NFKB1, MAPK10, AKT3, PIK3R1, RAD51	2.275930521	0.01430832	0.10961967
KEGG_PATHWAY	hsa04728:Dopaminergic synapse	PPP2R1B, PRKCA, SCN1A, PPP2R3A, GNAI3, GNAI2, CREB1, CAMK2G, MAPK10, ITPR3, PRKCB, FOS, GRIN2B, MAPK13, GSK3B, AKT3, CALM2, PPP2R2A, CACNA1B	1.829931116	0.01442364	0.10961967
KEGG_PATHWAY	*hsa04020:Calcium signaling pathway [40]	PRKCA, SLC8A3, ORAI2, SLC8A1, NOS1, SLC25A4, ERBB4, PHKB, ADORA2A, ERBB2, CAMK2G, PPP3R1, PTGFR, ITPR3, GRM1, PRKCB, CAMK4, ATP2A2, PDE1C, CACNA1G, PDGFRA, CALM2, HTR2A, CACNA1B	1.652910434	0.01751717	0.12597893
KEGG_PATHWAY	hsa04115:p53 signaling pathway	PPM1D, CCND2, BBC3, TP53, MDM2, APAF1, MDM4, PMAIP1, PERP, THBS1, ATM, SESN3	2.207992297	0.01775128	0.12597893
KEGG_PATHWAY	hsa04380:Osteoclast differentiation	CALCR, NCF2, PIK3CB, SOCS3, CREB1, TGFBR1, PPARG, PPP3R1, NFKB1, MAPK10, ITGB3, TAB2, FOS, CAMK4, MAPK13, NFATC2, TRAF6, PIK3R1, AKT3	1.788024296	0.01799699	0.12597893
KEGG_PATHWAY	hsa04110:Cell cycle	E2F3, CDC14A, RBL1, TP53, SMAD4, ESPL1, SKP1, SMC3, WEE1, ATM, CDKN1C, YWHAG, EP300, CDKN1B, CCND2, GSK3B, MDM2, ANAPC7	1.789542144	0.02155388	0.14072992
KEGG_PATHWAY	hsa04066:HIF-1 signaling pathway [39,42,43]	PRKCA, PIK3CB, ERBB2, CAMK2G, NFKB1, ARNT, PRKCB, EP300, HIF1A, CDKN1B, EIF4E, INS, TEK, AKT3, PIK3R1	1.92624328	0.02172763	0.14072992

KEGG_PATHWAY	*hsa04662:B cell receptor signaling pathway [44]	NRAS, FOS, PIK3CB, GSK3B, PPP3R1, NFKB1, PIK3AP1, CD79A, NFATC2, NFATC3, AKT3, PIK3R1	2.14399252	0.02177602	0.14072992
KEGG_PATHWAY	*hsa04068:FoxO signaling pathway [10]	PIK3CB, NLK, TGFBR1, SMAD4, MAPK10, FOXO4, GRM1, BCL2L11, ATM, NRAS, EP300, CDKN1B, MAPK13, INS, CCND2, MDM2, PRKAA2, PIK3R1, AKT3	1.747993901	0.02222051	0.14072992
KEGG_PATHWAY	hsa05216:Thyroid cancer	NRAS, CCDC6, PPARG, TP53, TFG, TCF7L2, TPM3	2.975713756	0.02615399	0.16178982
KEGG_PATHWAY	hsa04261:Adrenergic signaling in cardiomyocytes	PPP2R1B, PRKCA, ATP1B1, CACNA2D1, PPP2R3A, GNAI3, GNAI2, CACNG8, CREB1, CAMK2G, CREM, CACNB3, CACNB4, TPM3, CACNA2D4, MAPK13, AKT3, CALM2, PPP2R2A	1.697327412	0.02898082	0.1752022
KEGG_PATHWAY	hsa04151:PI3K-Akt signaling pathway	PPP2R3A, FGF14, NFKB1, ITGB3, KIT, COL6A5, INS, TEK, PIK3AP1, PRKAA2, MYB, THBS1, AKT3, PIK3R1, PPP2R1B, PRKCA, COL4A4, PIK3CB, CREB1, TP53, PKN2, ITGA4, BCL2L11, KDR, DDIT4, EIF4B, NRAS, HSP90B1, YWHAG, EIF4E, CDKN1B, CCND2, GSK3B, PDGFRA, MDM2, RELN, EFNA4, PPP2R2A, IL2	1.393595138	0.03014876	0.17821266
KEGG_PATHWAY	hsa05223:Non-small cell lung cancer	PRKCA, NRAS, E2F3, PIK3CB, ERBB2, TP53, RARB, AKT3, PIK3R1, PRKCB	2.201420891	0.03446471	0.1992959
KEGG_PATHWAY	hsa05031:Amphetamine addiction	PRKCA, FOS, GRIN2B, CAMK4, CAMK2G, CREB1, PPP3R1, GRIN3A, PDYN, CALM2, PRKCB	2.054659498	0.03826716	0.21206387
KEGG_PATHWAY	hsa04720:Long-term potentiation	PRKCA, NRAS, EP300, GRIN2B, CAMK4, CAMK2G, PPP3R1, ITPR3, GRM1, CALM2, PRKCB	2.054659498	0.03826716	0.21206387
KEGG_PATHWAY	hsa04713:Circadian entrainment	PRKCA, FOS, GNAI3, NOS1, GRIN2B, GNAI2, CREB1, ADCYAP1R1, CAMK2G, CACNA1G, GUCY1A2, ITPR3, CALM2, PRKCB	1.816751556	0.04170603	0.21916603
KEGG_PATHWAY	hsa05412:Arrhythmogenic right ventricular cardiomyopathy (ARVC)	CACNA2D1, CACNG8, SGCD, CACNB3, CACNB4, ITGA4, CDH2, ITGB3, CTNNA1, TCF7L2, CACNA2D4	2.023992939	0.04186644	0.21916603
KEGG_PATHWAY	*hsa04150:mTOR signaling pathway [45]	PRKCA, EIF4B, EIF4E, PIK3CB, INS, PRKAA2, AKT3, PIK3R1, PRKCB, DDIT4	2.125509826	0.04202055	0.21916603
GOTERM_BP_DIRECT	GO:0006355~regulation of transcription, DNA-templated	RORB, ZNF253, ZNF778, MED29, MAP3K9, ZNF773, ZFP90, RARB, RREB1, ZNF506, EMX2, ZNF791, ZNF649, ZNF793, PPARGC1A, ZNF37A, PRDM7, VGLL4, GPBP1L1, ZNF232, TADA1, ZNF619, MYBL1, MYT1, ZNF514, ZNF512, ZNF615, ZNF229, TCF20, INS, CASZ1, ZNF74, NKX2-4, TGFBR1, SMAD4, ZNF626, ZNF629, MZF1, ZNF117, ZNF385A, ZFHX3, TAF1B, ZNF486, EVX2, ZNF532, ZNF676, ZNF155, ZNF12, RLIM, ZBTB37, ZNF14, MBTD1, ZNF737, ZNF540, MKX, MYB, GATAD1, BHLHE41, ZNF493, USP13, ZBTB22, ZNF33A, ZFP30, ZNF544, ZNF354B, CNOT10, TP53, TLE3, RHOXF2B, ZNF333, ZBTB26, GTF2B, ZNF335, ZNF138, ZNF341, ZNF550, ZNF483, ZNF555, ZNF275, ZNF554, ZNF559, CREM, THAP5, ZNF35, ZNF655, ZNF652, ZNF30, ZFP36L2, SUMO1, NR1D2, GBX2, LIMD1, PRKAA2, USP34, ZNF568, NLK, FADS1, ZFP1, ZNF25, ZFP3, ZNF662, ZNF23, BCORL1, ZNF362, MAPK13, ZSCAN30, ZBTB2, NEUROD6, ZNF257, TP53INP1, FOXO4, GTF2E1, ZNF181, ZNF182, RNF141, MIER3, ZNF445, ZNF180, ZNF449, ZNF189, SIX4, RALGAPA1, HIF1A, EP300, IGSF1, ZNF431, ZNF436, ZNF841,	1.543674545	1.37E-10	5.92E-07

		ADORA2A, SOX5, ZMYND8, WT1, ZNF331, ZNF846, HNRNPD, AXIN2, ZNF420, RHOXF2, RUNX2, RFX8, RFX7, MSL3, ATF5, GCM2, ZNF317, EBF3, CDKN2AIP, RFX3, RSF1, ZKSCAN8, GPBP1, PAX6, HR, PAX5, ZKSCAN5, ZNF207, PRMT3, MAX, BZW1, HEY2, INO80D, KRBOX4, KHDRBS2, EGR2, DMRT3, ZFX, PKN2, ESR1, IRF2BP2, HMGA2, HOXC10, EYA1, ACVR2B, HIPK1, HIPK3, ZNF711, LCOR, IRX5, ZBTB8B, KMT2C, WBSCR22, NFYB, NFYA, DRGX, TSC22D3, ZNF512B, CHD7, CHD2, POM121C, PHF20L1, NFATC2, ZNF700, CHD3, CREB1, CBL, RCAN1, RNF44, ATXN3, SP1, SP5, IRF2, DENND4A			
GOTERM_BP_DIRECT	GO:0006351~transcription, DNA-templated	XRCC5, NAA15, RORB, ZNF253, MED20, GTF2IRD2B, ZNF778, MED29, MAP3K9, ZNF773, ZFP90, DHX36, RARB, ZNF506, ZHX2, MECP2, ZNF791, ZNF649, ZNF793, TOX3, ZNF37A, VGLL4, ERBB4, GPBP1L1, ERBB2, TFCP2L1, ZNF232, TADA1, ZNF619, ZNF514, MYT1, ZNF512, ZNF615, ARX, ZNF229, TCF20, HEXIM1, CASZ1, ZNF74, AEBP2, IKZF4, KLF7, KLF9, SMAD4, ZNF626, ZNF629, RNF4, ETS2, JAZF1, MZF1, ZNF117, ZNF385A, TAF1B, ZNF518B, ZNF18, ZNF486, ZNF532, EZH1, CCNT1, PPARG, ZNF155, ZNF676, ZNF12, ZEB1, RLIM, ZBTB37, ZNF14, MBTD1, ZNF737, ZNF540, NR2F2, BHLHE41, ZNF493, ZBTB22, ZBTB20, ZNF33A, POLR1D, ZFP30, ZNF544, CNOT10, ZNF354B, TP53, TLE3, POLR1A, ZNF333, MBD2, ZBTB26, ZNF335, FOXR2, ZNF138, SS18, ZNF341, BRWD1, USP21, ZNF550, ZSCAN18, TBX18, ZNF483, NSD1, ZNF555, ZNF275, ZNF554, ZNF559, CREM, THAP5, ZNF367, ZNF35, ZNF655, ZNF652, STAT6, ZNF30, NR1D2, BCL11A, GATAD2B, BCL9L, LIMD1, PRKAA2, FOXD3, ETV3, BRD2, ZNF568, NLK, ZNF24, ZFP1, ZNF25, ZFP3, ZNF662, POLR3E, ZNF23, ZNF865, ZNF362, MAPK13, BCORL1, ZSCAN30, SFPQ, ZBTB4, ZBTB2, NEUROD6, TP53INP2, ZNF257, TP53INP1, CBX6, ZNF181, ZNF182, MIER3, OLIG2, ZNF445, ZNF180, ZNF449, RBL1, ZNF189, FOXN2, ARID1A, SPOCD1, FOXN3, PA2G4, HIF1A, KDM2B, TIMELESS, ZNF431, ZNF436, TSHZ3, TSHZ2, ZNF841, ZNF331, ZNF846, ARNT, HNRNPD, ZNF420, CREBL2, RFX8, MAFG, ZMYM2, CREBZF, RNPS1, MED13L, FOXP1, SUV39H2, MSL3, PHF3, PHF2, ZNF317, EBF3, RFX3, BMI1, E2F3, ZKSCAN8, GPBP1, DEDD, NR6A1, HR, PAX6, PAX5, ZKSCAN5, BZW1, HEY2, INO80D, ZNF280B, EGR2, KHDRBS2, DMRT3, ZFX, PKN2, ESR1, CCNL1, RUNX1T1, IRF2BP2, PURA, PRKCB, HOXC10, EYA1, HIPK1, BTG2, ZNF711, ZNF710, ZFPM2, CUX2, LCOR, IRX5, ZBTB8B, KMT2C, WBSCR22, NFYB, NFIX, TCF7L2, DRGX, HIC2, CHD8, ZNF512B, CHD7, POU2F1, CHD2, MLXIP, ZNF700, CHD3, TXNIP,	1.440361592	1.07E-09	2.31E-06

		ZBTB7A, HMBOX1, ATXN7L3, ATXN1, ATXN3, SP5, DENND4A, URI1, NFIB			
GOTERM_BP_DIRECT	GO:0045893~positive regulation of transcription, DNA-templated	E2F3, RSF1, GPBP1, PPARG, NAA15, PAX6, CTCF, RORB, NFKB1, GLI2, IL31RA, FOS, PICALM, MYOCD, ZFP90, NR2F2, MYB, IRAK1, EGR2, RREB1, SOX11, TP53, MECP2, ESR1, FOXN2, ARID1A, SIX4, HMGA2, PPARGC1A, TOX3, FOXN3, HIF1A, CAMK4, CD80, ZNF711, USP21, SMARCA5, NSD1, MED1, KMT2A, ERBB4, GPBP1L1, ELK1, AFAP1L2, NFYB, MYBL1, NFYA, WT1, ARNT, PHIP, CHD8, NR1D2, HNRNPD, NFATC2, RUNX2, FBXW11, NFATC3, CREBL2, KLF7, NOS1, TGFBR1, CREB1, HMBOX1, FZD1, SMAD4, FZD4, ATXN7L3, CDKN1C, ATF5, SP1, RNF4, ETS1, ETS2, LRP6, RFX3, ZFHX3, TP53INP2, TP53INP1	1.740762744	1.67E-06	0.00240242
GOTERM_BP_DIRECT	GO:0000122~negative regulation of transcription from RNA polymerase II promoter	IMPACT, SNCA, CBX6, MYOCD, ZFP90, RARB, OLIG2, RREB1, GABPA, RBL1, ZHX2, MECP2, ZNF649, ZNF189, ARID1A, EP300, KDM2B, TIMELESS, MTF2, ZNF431, TSHZ3, TSHZ2, TFCP2L1, TAF9B, WT1, ARX, NIPBL, HEXIM1, TRAF6, AEBP2, SMAD4, EHMT2, FOXP1, SUV39H2, FNIP2, CDKN1C, TRPS1, ETS2, JAZF1, MZF1, ZFHX3, BMI1, CPEB3, DICER1, PPARG, NR6A1, PAX6, WWC2, PAX5, CTCF, NFKB1, ZEB1, GLI2, RLIM, ZNF148, HEY2, NR2F2, BHLHE41, MYB, ZNF280B, ZBTB20, SOX11, SLA2, TP53, ESR1, HMGA2, MBD2, MBD1, ACVR2B, BTG2, MDM2, ZFPM2, CUX2, MDM4, TBX18, NSD1, MED1, FRK, GLIS3, USP3, NFIX, TCF7L2, STAT6, TSC22D3, CHD8, REL, BCL11A, GATAD2B, NEDD4L, NFATC2, FOXD3, ETV3, TXNIP, ZBTB7A, DACH1, SFPQ, ZBTB4, IRF2, URI1, NFIB	1.596319112	2.80E-06	0.00301074
GOTERM_BP_DIRECT	GO:0045892~negative regulation of transcription, DNA-templated	XRCC5, RSF1, PPARG, HR, ZNF12, CTCF, RORB, ZEB1, ZNF253, RLIM, PDCD4, ZNF148, HEY2, ZNF540, NR2F2, BHLHE41, MYB, NRG1, SOX10, ZNF280B, MTA3, TP53, RUNX1T1, ZHX2, MECP2, BASP1, SIX4, HMGA2, MBD2, MBD1, FOXN3, PURA, PA2G4, EREG, TIMELESS, MDM2, ZFPM2, TSHZ3, CREM, TCF7L2, WT1, HIC2, SUMO1, CHD8, NIPBL, SET, NR1D2, HEXIM1, PRAMEF2, POU2F1, LIMD1, TRAF6, RUNX2, FBXW11, ZBTB7A, IKZF4, CREBZF, HMBOX1, ZNF24, SMAD4, FZD1, DACH1, FOXP1, SUV39H2, ATXN1, CDKN1C, ATF5, CDKN1B, SFPQ, ZBTB4, USP47, RFX3, ZFHX3	1.681413477	1.28E-05	0.01105604
GOTERM_BP_DIRECT	GO:0045944~positive regulation of transcription from RNA polymerase II promoter	LMO4, CASK, JAG1, FOXO4, GDNF, CBFB, MYOCD, DHX36, RARB, PHOX2B, GABPA, RBL1, ZNF649, SIX4, PPARGC1A, GRHL2, DCAF6, HIF1A, EP300, MTF1, MTF2, ARRB1, PYGO2, SLC40A1, CAMTA1, ONECUT3, ONECUT2, TAF9B, ELK1, MYBL1, WT1, ARNT, TCF20, NIPBL, HOXA5, AUTS2, TRAF6, MAFG, IKZF4, IKZF3, SMAD4, ARID3B, TET2, FOXP1, ATF5, GCM2, TET3, RNF4, ETS1, EBF3, ETS2, TRPS1,	1.452796843	1.81E-05	0.01299448

		ARF4, MZF1, RFX3, TCF12, ATAD2B, NAMPT, EZH1, NR6A1, PPARG, CCNT1, PAX6, PAX5, CTCF, NFKB1, ZEB1, GLI2, FLT3LG, FOS, ZNF148, HEY2, NRG1, ALX4, MYB, CYR61, SOX10, EGR2, SOX11, CCNL1, TP53, CDK8, ESR1, HMGA2, SS18, EYA1, KPNA6, ZFPM2, MED1, GLIS3, KMT2A, CREM, PPP3R1, NFIX, TCF7L2, STAT6, PHIP, CHD8, CHD7, REL, BCL11B, BCL11A, GBX2, NFAT5, BCL9L, YES1, NFATC2, NFATC3, PIK3R1, FOXD3, NOS1, NUCKS1, CREB1, CAPRIN2, SP1, GSK3B, CDON, LRP6, IRF2, PBX3, NEUROD6, IL2, NFIB, BMPR1A			
GOTERM_BP_DIRECT	GO:0006366~transcription from RNA polymerase II promoter	LMO4, CCNT1, PAX6, PAX5, CTCF, NFKB1, FOXO4, GLI2, MED20, CBFB, MAX, FOS, GTF2E1, MYOCD, ZNF148, ALX4, MYB, PHOX2B, SOX10, EGR2, RREB1, SOX11, GABPA, TP53, ESR1, SIX4, HMGA2, GTF2B, MBD1, GRHL2, HIF1A, EP300, MTF1, ARRB1, GLIS3, CAMTA1, KMT2A, ONECUT3, ONECUT2, TAF9B, SOX5, ELK1, NFIX, NFYA, MYBL1, WT1, REL, HOXA5, BCL11B, GBX2, NFAT5, VEZF1, NFATC2, RUNX2, NFATC3, MAFG, IKZF3, NUCKS1, CREB1, SMAD4, ARID3B, ATF5, GCM2, EBF3, ETS1, TRPS1, MZF1, IRF2, PBX3, NEUROD6, ZFHX3, TCF12, NFIB	1.63552695	3.27E-05	0.02012512
GOTERM_BP_DIRECT	GO:0007399~nervous system development	PCDHA6, PCDHA7, PCDHA8, FUT9, PCDHA2, ERBB4, PCDHA3, SCN3B, FGF14, PCDHA4, CAMK2G, PCDHA5, GPM6B, L1CAM, JAG1, PCDHA1, MYLIP, ST8SIA2, MYT1, GDNF, PCDHAC2, PCDHAC1, EPHB2, FOS, ROBO1, SMARCD1, GBX2, DLG4, PCDHA10, PCDHA11, NRG1, DLG2, NTF3, SCN2B, ARHGEF7, MAP1B, PCDHB4, MBD5, PURA, ATXN3, EP300, ZIC5, DYRK1A, APAF1, NEUROD6, GFRA2	1.842163379	7.53E-05	0.04049863
GOTERM_BP_DIRECT	GO:0048701~embryonic cranial skeleton morphogenesis	IRX5, TGFBR1, PDGFRA, PAX5, MMP16, SIX4, MMP14, RUNX2, GRHL2, SLC39A1	3.707579872	9.58E-04	0.45827571
GOTERM_BP_DIRECT	GO:0007411~axon guidance	NRP2, PAX6, L1CAM, GLI2, CXCL12, EPHB2, DRGX, ARX, RANBP9, WNT3, ROBO1, ANK3, GBX2, KLF7, ZNF280B, CREB1, PTPRA, SMAD4, NFASC, NRAS, CNTN2, SPTBN1, RELN, SIAH2, EFNA4, EXT1, KIF26B	1.951726008	0.00124894	0.4835088
GOTERM_BP_DIRECT	GO:0043066~negative regulation of apoptotic process	STIL, IL6ST, SNCA, NAA15, NFKB1, GLI2, PDCD4, GDNF, IL31RA, BAG1, ITCH, RARB, CYR61, SOX10, IRAK1, SOCS3, TP53, WNK3, SIX4, HMGA2, FLNA, PA2G4, CCND2, HIPK3, SERPINB2, MDM2, SIAH2, MDM4, SLC40A1, MED1, CAMK1D, ERBB4, PAFAH2, PKHD1, TAF9B, WT1, PHIP, STK40, PRAMEF2, BCL11B, TEK, PRKAA2, AGO4, PALB2, TRAF6, THBS1, PIK3R1, CFLAR, PDCD10, CBL, PTGFR, KDR, IFIT3, ATF5, HSP90B1, CDKN1B, GSK3B, ARF4, USP47, IL2	1.515626058	0.0012551	0.4835088
GOTERM_BP_DIRECT	GO:0008284~positive regulation of cell proliferation	NAMPT, E2F3, PRC1, IL6ST, NAP1L1, GDNF, FLT3LG, IL31RA, CD47, MYOCD, WDR77, CEACAM6, SHC1, GDF9, RARB, NRG1, CDK13, SHC4, STX3,	1.504513635	0.00136835	0.4835088

		SOX11, MTA3, LIFR, MECP2, PURA, EIF4G1, HOXC10, CRKL, EREG, HIPK1, CCND2, PDGFRA, MDM2, EIF5A2, CCK, GNAI2, ERBB4, PKHD1, CD248, ST8SIA1, KIT, TSPYL5, PHIP, INS, PRAMEF2, ADRA2A, BRK1, THBS1, RUNX2, PGGT1B, ACER3, PDCD10, NTF3, TGFBR1, HILPDA, PTGFR, KDR, CNTF, CDKN1B, ETS1, IL2, HTR2A			
GOTERM_BP_DIRECT	GO:0007223~Wnt signaling pathway, calcium modulating pathway	TNRC6C, NLK, PPP3R1, AGO3, FZD3, AGO4, TNRC6B, FZD4, TNRC6A, TCF7L2, CALM2	3.241755735	0.00146007	0.4835088
GOTERM_BP_DIRECT	GO:0035194~posttranscriptional gene silencing by RNA	TNRC6C, AGO3, AGO4, TNRC6B, TNRC6A	8.209641146	0.00160713	0.49419179
GOTERM_BP_DIRECT	GO:0006461~protein complex assembly	PPP2R1B, PARD3, KMT2A, MAGI1, TP53, MPP6, PPARGC1A, WAS, CARD10, MAX, KCNQ5, RANBP9, PICALM, ZNF148, DLG4, MDM2, MDM4, TRAF6, ADD2, APC, ZW10	2.080719394	0.00229822	0.62486034
GOTERM_BP_DIRECT	GO:0061158~3'-UTR-mediated mRNA destabilization	ZFP36L2, UPF1, CPEB3, HNRNPD, QKI, RC3H1	5.746748802	0.00232236	0.62486034
GOTERM_BP_DIRECT	GO:0006357~regulation of transcription from RNA polymerase II promoter	ZNF518B, ZNF18, NFKB1, ZEB1, MED20, GTF2IRD2B, CBFB, FOS, WDR77, SMARCD1, NR2F2, SOX10, RBL1, ARID1A, PURA, FOXR2, PRKCB, BRWD3, BRWD1, MTF1, SMARCA5, ZNF710, ZSCAN18, GLIS3, TSHZ3, TSHZ2, TFCP2L1, ZNF367, TADA1, TCF7L2, WT1, STAT6, ZNF704, CHD2, VEZF1, NFATC3, RUNX2, CHD3, ETV3, MAFG, RFX8, KLF7, IKZF3, ZMYM2, BRD2, KLF9, NUCKS1, CREB1, RFX7, MED13L, ZNF662, FOXP1, ZNF865, ATF5, PRKAR1A, URI1, TCF12	1.485554112	0.00264319	0.66934793
GOTERM_BP_DIRECT	GO:0016567~protein ubiquitination	TRIML2, SYVN1, PAX6, UBA6, MYLIP, KLHL4, RLIM, ASB15, KLHL3, MED20, ASB18, ZNRF3, ARIH1, DCAF12, TRIM2, ATG7, KBTBD8, FBXW2, FBXO4, ITCH, NEDD4L, DCAF17, FBXW11, RC3H1, DTX4, NUB1, SOCS3, CBL, UBE2J1, PDZRN4, GAN, SKP1, UBR1, MSL2, DCAF6, RNF44, KLHL18, DCAF7, RNF4, ARRB1, TRIM32, WSB2, FBXL5, MDM2, FBXL22, MDM4, RNF135, MED1	1.536735056	0.00306582	0.7332416
GOTERM_BP_DIRECT	GO:0007169~transmembrane receptor protein tyrosine kinase signaling pathway	FRK, MPZL1, NTF3, ERBB4, PIK3CB, ERBB2, KIT, KDR, IL31RA, TEK, NPTN, ROR1, CD4, NRG1, YES1, ABL2, GFRA2, SHC4	2.155030801	0.00355984	0.79346922
GOTERM_BP_DIRECT	GO:0017148~negative regulation of translation	CPEB3, FMR1, NANOS1, SYNCRIP, IGF2BP1, IGF2BP2, WT1, PURA, CAPRIN2, EIF4E, BTG2, ZNF540, GIGYF2	2.576128773	0.00368627	0.79346922
GOTERM_BP_DIRECT	GO:0009612~response to mechanical stimulus	TXNIP, BTG2, ETS1, PPARG, RCAN1, THBS1, GLI2, MMP14, MBD2, CLCN6, PIEZO2, KCNK2, CXCL12	2.532465574	0.00426924	0.86677347
GOTERM_BP_DIRECT	GO:0007409~axonogenesis	PARD3, CCK, CREB1, LRRC38, SLITRK2, NRCAM, SLITRK4, PICALM, SLITRK3, ANK3, GSK3B, NUMB, ATP8A2, CNTN2, STMN1, SLITRK6, SLITRK5, LRFN2	2.11105058	0.0044295	0.86677347
GOTERM_BP_DIRECT	GO:0060402~calcium ion transport into cytosol	SLC8A3, CACNA2D1, SLC8A1, JPH2, CACNB3, ITPR3	4.925784688	0.00506653	0.94832295
GOTERM_BP_DIRECT	GO:0045664~regulation of neuron differentiation	EYA1, YWHAG, NTF3, BCL11B, CDON, CASZ1, ZFHX3	4.022724162	0.00570433	1
GOTERM_BP_DIRECT	GO:0006888~ER to Golgi vesicle-mediated transport	STX5, DYNC1LI2, SEC24B, VAPA, VAPB, VTI1B, DCTN5, VTI1A, RAB1A, TMED7, MPPE1, MIA3, COG6, ANK3, INS, CNIH4, ARF4, BCAP29, SPTBN1, GOSR2, GOSR1, SEC23IP, TBC1D20, TMED7-TICAM2, ZW10	1.795859001	0.00587605	1
GOTERM_BP_DIRECT	*GO:0007155~cell adhesion [17]	NRP2, PCDHA6, PCDHA7, ATP1B1, PCDHA8, PCDHA2,	1.427297088	0.00617881	1

		PCDHA3, TLN2, PCDHA4, PCDHA5, CASK, L1CAM, PCDHA1, CXCL12, CD47, CD96, ROBO1, TGFBI, CNTNAP2, CYR61, PRKCA, MAGI1, FLOT2, PCDHB4, AJAP1, CTNNA1, PRKCE, GRHL2, CNTN2, SUSD5, RELN, DST, PLXNC1, CYP1B1, ITGB3, CDH2, PCDHAC2, PCDHAC1, ISLR, ALCAM, ITGAX, COL6A5, TOR1A, PCDHA10, CD4, PCDHA11, THBS1, ENTPD1, APC, LPP, PCDH10, ITGA4, ATP2A2, CDON, ADAM22, ABL2, IL2			
GOTERM_BP_DIRECT	GO:0042787~protein ubiquitination involved in ubiquitin-dependent protein catabolic process	RNF144A, HECW2, SYVN1, CBL, UBA6, GAN, MYLIP, KLHL3, BTBD9, LNX1, ARIH1, KBTBD4, C18ORF25, RNF165, TRIM32, HECTD2, MDM2, ANKIB1, NEDD4L, ANAPC7, ITCH, CUL4B, SIAH2, TRIP12	1.802901585	0.00672477	1
GOTERM_BP_DIRECT	GO:0050821~protein stabilization	ATP1B1, SYVN1, PDCD10, CREB1, NLK, FLOT2, TAF9B, NAA15, ZSWIM7, PPARGC1A, FLNA, ZNF207, SUMO1, GOLGA7, EP300, CCT4, AAK1, MDM4, CDC37L1, PIK3R1, USP13, CREBL2	1.85924226	0.00688931	1
GOTERM_BP_DIRECT	GO:0048863~stem cell differentiation	GPM6A, PSMD11, MTF2, KIT, HMGA2, FOXO4, RUNX2, SHC4	3.405480772	0.00695881	1
GOTERM_BP_DIRECT	GO:0072659~protein localization to plasma membrane	CALCR, MYO5A, ATP1B1, ZDHHC22, TNIK, SCN3B, ANK3, PPIL2, LRP6, RAB11A, CACNB3, SCP2, FLNA	2.371674109	0.00737792	1
GOTERM_BP_DIRECT	GO:0042752~regulation of circadian rhythm	TIMELESS, NR1D2, SFPQ, CREB1, CREM, PPARG, HNRNPD, RORB, PRKAA2, MAPK10, PPARGC1A	2.580172932	0.00852549	1
GOTERM_BP_DIRECT	GO:0006486~protein glycosylation	GALNT1, ST6GAL1, FUT9, GALNT6, ST8SIA1, OAS2, ST8SIA2, POMGNT1, GALNT10, B3GNT5, B3GNT6, ST3GAL6, SRD5A3, GALNT16, FUT1, GALNT18, EXT1, LRP2, DDOST	1.932534995	0.00860702	1
GOTERM_BP_DIRECT	GO:0007507~heart development	NRP2, ERBB4, ERBB2, PPARG, GLI2, WT1, ROBO1, HEXIM1, CASP7, GAB1, TEK, SHC1, SH3PXD2B, SMG9, TGFBR1, NF1, ADIPOR2, MBD2, MBD1, TAB2, ATM, EIF4G2, ACVR2B, EP300, CRKL, TRPS1, ADAM19	1.695761942	0.00868043	1
GOTERM_BP_DIRECT	GO:0001764~neuron migration	PHOX2B, CCK, MDGA1, PAX6, FZD3, CXCL12, MARK1, DDIT4, DRGX, NRCAM, GPM6A, MAPT, CNTN2, RELN, ALKBH1, PAFAH1B1, KIAA0319, NR2F2	1.970313875	0.0089388	1
GOTERM_BP_DIRECT	GO:0035162~embryonic hemopoiesis	HIF1A, KMT2A, KIT, KDR, MED1, FLT3LG	4.310061602	0.00954782	1
GOTERM_BP_DIRECT	GO:0003222~ventricular trabecula myocardium morphogenesis	CHD7, TGFBR1, HEY2, NRG1, BMPR1A, MED1	4.310061602	0.00954782	1
GOTERM_BP_DIRECT	GO:0009791~post-embryonic development	GABRG2, ACADM, KMT2A, ACO1, TGFBR1, MECP2, SZT2, TET2, ATF5, ACVR2B, PYGO2, ALX4, GIGYF2, PLEKHA1	2.204232417	0.00962157	1
GOTERM_BP_DIRECT	GO:0048015~phosphatidylinositol-mediated signaling	PIRT, ERBB4, PIK3CB, ERBB2, NPR3, KIT, TNRC6C, EREG, CD80, GAB1, PDGFRA, AGO3, AGO4, TNRC6B, NRG1, TNRC6A, FRS2, PIK3R1	1.951726008	0.00980894	1
GOTERM_BP_DIRECT	GO:0007156~homophilic cell adhesion via plasma membrane adhesion molecules	PCDHA6, PCDHA7, PCDHA8, PCDHA9, PCDHA2, PCDHA3, PIK3CB, PCDHA4, PCDHB4, PCDHA5, PCDH10, PCDHA1, CDH2, PCDHAC2, PCDHAC1, CD84, CDH7, ROBO1, FAT2, NPTN, PCDHA10, PCDHA11, PCDHA12, PCDHA13	1.745847737	0.00984875	1
GOTERM_BP_DIRECT	GO:0007268~chemical synaptic transmission	MYO5A, SYT1, SNCB, CACNB3, CACNB4, GJC1, AMPH, KCNQ5, KCNQ3, GRIN2B, DLG4, PAFAH1B1, SLC1A1, NOVA1, DLG2, DTNA, LRFN2, GJD2, SCN2B, SYT10, FLOT2, PCDHB4,	1.580355921	0.00995408	1

		BSN, PDYN, GRM1, ATXN3, CACNA1G, AKAP5, LRP6, PMP22, SLITRK5, HTR2A, CACNA1B			
GOTERM_BP_DIRECT	GO:0006896~Golgi to vacuole transport	VPS54, VTI1B, GOSR2, VTI1A	7.662331736	0.01073243	1
GOTERM_BP_DIRECT	GO:0006449~regulation of translational termination	UPF1, MTRF1, GLE1, ETF1	7.662331736	0.01073243	1
GOTERM_BP_DIRECT	GO:0035556~intracellular signal transduction	ITSN1, GUCY1A2, SHC1, NRG1, AKT3, SHC4, PRKCA, TNIK, ARHGEF7, PKN2, WNK3, PRKCE, MARK1, SRPK1, DCDC1, PRKCB, SS18, MAST4, CRKL, KSR2, CAMK4, CD80, STMN1, KSR1, STYXL1, GNAI2, ARHGEF28, KIT, ASB15, ASB18, PLCL2, SH3BP5L, CORO2A, DGKG, PRKAA2, STK38L, GPR155, TGFBR1, NLK, SMAD4, DGKH, DGKI, RACGAP1, CCDC68, PDZD8, MAPK13, GSK3B, PRKAR1A, WSB2, RGS6	1.425992259	0.01083165	1
GOTERM_BP_DIRECT	GO:0061025~membrane fusion	SNAP29, STX5, STX3, DNM1L, VAPA, ATG7, VTI1B, GOSR2, SNAP23, STX1B	2.612158546	0.01210765	1
GOTERM_BP_DIRECT	GO:0010468~regulation of gene expression	BMI1, PHOX2B, ATP1B1, TSHZ2, TGFBR1, KIAA0430, NF1, SART3, STX1B, GDNF, HIF1A, NIPBL, STK40, SORT1, ALKBH1, POFUT2, SHC4	1.953894593	0.01226181	1
GOTERM_BP_DIRECT	GO:1900740~positive regulation of protein insertion into mitochondrial membrane involved in apoptotic signaling pathway	YWHAG, TP53BP2, BBC3, PPP3R1, TP53, PMAIP1, BMF, BCL2L11	3.064932695	0.01269012	1
GOTERM_BP_DIRECT	GO:0045773~positive regulation of axon extension	EIF4G2, MAPT, GSK3B, MAP1B, RAB11A, PAFAH1B1, L1CAM, NRG1	3.064932695	0.01269012	1
GOTERM_BP_DIRECT	GO:0030307~positive regulation of cell growth	ERBB2, TGFBR1, TAF9B, MMP14, EIF4G1, EIF4G2, KDM2B, INS, CDKN2AIP, TRIM32, USP47, H3F3A, ACSL4, NRG1, IL2	2.052410286	0.01310856	1
GOTERM_BP_DIRECT	GO:0007420~brain development	XRCC5, SYT1, SEPT4, CKB, CHD8, NIPBL, B3GNT5, CNTNAP2, SMG9, EGR2, MDGA1, NF1, AFF2, GRHL2, SLC7A11, ZNF335, ATM, BCL2L11, DDIT4, RAB18, ARF4, PYGO2, H3F3A, RELN, STMN1, ZFHX3, MED1	1.633286502	0.01379889	1
GOTERM_BP_DIRECT	GO:0001666~response to hypoxia [39,46]	NOX4, ATP1B1, NOS1, CREB1, NF1, SMAD4, APOLD1, MECP2, MMP14, CXCL12, ATM, PRKCB, DDIT4, ARNT, HSP90B1, EP300, HIF1A, CDKN1B, MYOCD, ETS1, TEK, CHRNB2, LIMD1, APAF1, THBS1	1.670566512	0.0140127	1
GOTERM_BP_DIRECT	GO:0007422~peripheral nervous system development	SOX10, EGR2, ERBB2, NF1, NFASC, PMP22, NRG1	3.352270135	0.01468063	1
GOTERM_BP_DIRECT	GO:0007154~cell communication	SLC8A3, GJD2, GJD4, SLC8A1, KREMEN1, NF1, TRIP10, NRG1, GJC1	2.722144169	0.01482516	1
GOTERM_BP_DIRECT	GO:0007205~protein kinase C-activating G-protein coupled receptor signaling pathway	PARD3, CCK, ADORA2A, DGKG, DGKH, DGKI, GRM1, IL2	2.966063898	0.01519187	1
GOTERM_BP_DIRECT	GO:0035278~miRNA mediated inhibition of translation	TNRC6C, AGO3, AGO4, TNRC6B, TNRC6A	4.788957335	0.01595896	1
GOTERM_BP_DIRECT	GO:0070989~oxidative demethylation	TET3, CYP2C8, FTO, ALKBH1, TET2	4.788957335	0.01595896	1
GOTERM_BP_DIRECT	GO:0042552~myelination	SLC8A3, MYO5A, EGR2, ERBB2, NFASC, ZNF24, QKI, OLIG2, PMP22, FAM126A	2.498586436	0.01609938	1
GOTERM_BP_DIRECT	GO:0048511~rhythmic process	SP1, NR1D2, SFPQ, CREM, PPARG, RORB, PRKAA2, NFYA, MAPK10, FBXW11, SUV39H2	2.341268031	0.01674226	1
GOTERM_BP_DIRECT	GO:0030335~positive regulation of cell migration	PRKCA, PDCD10, NTF3, TGFBR1, ONECUT2, SUN2, KIT, CCL5, LRRC15, MMP14, KDR, SEMA6D, SEMA3G, INS, RRAS2, PTP4A1, NUMB, TRIM32, ADRA2A, PDGFRA, CEACAM6, THBS1, PIK3R1, CYR61, APC, ATP8A1	1.624081183	0.01685678	1

GOTERM_BP_DIRECT	GO:0001844~protein insertion into mitochondrial membrane involved in apoptotic signaling pathway	BBC3, PMAIP1, BMF, BCL2L11	6.567712917	0.0175764	1
GOTERM_BP_DIRECT	GO:0051151~negative regulation of smooth muscle cell differentiation	RBPMS2, EREG, RCAN1, FOXO4	6.567712917	0.0175764	1
GOTERM_BP_DIRECT	GO:0048589~developmental growth	SOX10, NIPBL, SMAD4, PYGO2, ALKBH1, GLI2, BMPR1A	3.218179329	0.0179301	1
GOTERM_BP_DIRECT	GO:0051899~membrane depolarization	KMT2A, ADORA2A, SCN3B, CHRNB2, CACNB3, CACNB4, CACNA1B	3.218179329	0.0179301	1
GOTERM_BP_DIRECT	GO:0035264~multicellular organism growth	STIL, DUOX2, PKDCC, GRHL2, CDKN1C, ATF5, STK40, HOXA5, H3F3A, RARB, SLITRK6, PALB2, GIGYF2, PLEKHA1	2.011362081	0.02003426	1
GOTERM_BP_DIRECT	GO:0048812~neuron projection morphogenesis	CTTN, SLC9A6, NTF3, GPM6A, ADORA2A, DICER1, ZSWIM6, DYNLT1, ZNF335, WEE1	2.394478668	0.02097842	1
GOTERM_BP_DIRECT	GO:0030148~sphingolipid biosynthetic process	ACER3, VAPA, VAPB, SPTLC3, ELOVL3, CERS5, ALDH3B2, KDSR, CERS3, SGMS1	2.394478668	0.02097842	1
GOTERM_BP_DIRECT	GO:2001244~positive regulation of intrinsic apoptotic signaling pathway	SEPT4, DNM1L, BBC3, PRKRA, TP53, PMAIP1, BMF, BCL2L11	2.78630245	0.02120987	1
GOTERM_BP_DIRECT	GO:0002088~lens development in camera-type eye	TGFBR1, CDON, PAX6, PYGO2, SLITRK6, NHS, MED1, WNT2B	2.78630245	0.02120987	1
GOTERM_BP_DIRECT	GO:0090110~cargo loading into COPII-coated vesicle	MIA3, TBC1D20, RAB1A	11.4934976	0.02135263	1
GOTERM_BP_DIRECT	GO:0071494~cellular response to UV-C	POLH, IMPACT, MDM2	11.4934976	0.02135263	1
GOTERM_BP_DIRECT	GO:0051612~negative regulation of serotonin uptake	NOS1, SNCA, GPM6B	11.4934976	0.02135263	1
GOTERM_BP_DIRECT	GO:0008340~determination of adult lifespan	BBC3, TFCP2L1, TP53, RAD54L, ATM	4.420576002	0.02149378	1
GOTERM_BP_DIRECT	GO:0060996~dendritic spine development	SLC9A6, ARF4, UBA6, ACSL4, EPHB2	4.420576002	0.02149378	1
GOTERM_BP_DIRECT	GO:0045070~positive regulation of viral genome replication	TRIM38, ADARB1, VAPB, PKN2, CCL5, SRPK1, LARP1	3.094403201	0.02163971	1
GOTERM_BP_DIRECT	GO:0007049~cell cycle	SEPT4, PARD3, E2F3, RABGAP1, GNAI3, GNAI2, CCNT1, THAP5, WTAP, MAP3K8, PARD3B, FAM32A, CREBL2, TXNIP, SEPT14, TP53BP2, RBL1, TP53, PKN2, PRKCE, APPL1, TET2, SUV39H2, MAPK13, CCND2, PTP4A1, TMPRSS11A, CUL4B, SIAH2	1.535997376	0.02256095	1
GOTERM_BP_DIRECT	GO:0007612~learning	SLC8A3, ARF4, DLG4, CNTN2, UBA6, CNTNAP2, CHRNB2, SHANK2, ATP8A1, EPHB2, SORCS3	2.218043397	0.02387886	1
GOTERM_BP_DIRECT	GO:0044829~positive regulation by host of viral genome replication	VAPA, NUCKS1, VAPB, TBC1D20	5.746748802	0.02632625	1
GOTERM_BP_DIRECT	GO:0035196~production of miRNAs involved in gene silencing by miRNA	PRKRA, DICER1, AGO3, AGO4	5.746748802	0.02632625	1
GOTERM_BP_DIRECT	GO:0007143~female meiotic division	EREG, KIAA0430, PRKAR1A, LFNG	5.746748802	0.02632625	1
GOTERM_BP_DIRECT	GO:0010506~regulation of autophagy	SOGA1, EP300, SOGA3, MAPT, USP10, VMP1, ABL2, ATM, USP13, TP53INP1	2.298699521	0.02683876	1
GOTERM_BP_DIRECT	GO:0045821~positive regulation of glycolytic process	HIF1A, INS, PRKAA2, ARNT, HTR2A	4.104820573	0.02806467	1
GOTERM_BP_DIRECT	GO:0010801~negative regulation of peptidyl-threonine phosphorylation	EIF4G1, PARD3, SPRED2, CALM2, DDIT4	4.104820573	0.02806467	1
GOTERM_BP_DIRECT	GO:1900153~positive regulation of nuclear-transcribed mRNA catabolic process, deadenylation-dependent decay	ZFP36L2, TNRC6C, CPEB3, NANOS1, TNRC6B	4.104820573	0.02806467	1
GOTERM_BP_DIRECT	GO:0043161~proteasome-mediated ubiquitin-dependent protein catabolic process	CSNK1A1, RAD23B, TP53, SKP1, RLIM, BTBD9, PSMB11, UBE2R2, TRIM38, PSMF1, PSMC6, RNF44, ATXN3, KBTBD4, C18ORF25, RNF4, PSMD11, RNF165, ARRB1, GSK3B, UBE2W, FBXL22, ANAPC7, SIAH2, NEDD4L, FBXW11, APC	1.5286918	0.02948846	1
GOTERM_BP_DIRECT	GO:0098609~cell-cell adhesion	CAST, VAPA, ZC3HAV1, VAPB, EIF5, ASAP1, ESYT2, CAPZB, RAB1A, IQGAP1, LARP1, BZW1,	1.441988629	0.03037818	1

		CTTN, PICALM, NUMB, EHD1, VASN, STX5, MYO1B, STK24, CBL, PKN2, MPP7, MPRIP, EIF4G1, TNKS1BP1, EIF4G2, TMEM47, CRKL, UBFD1, IST1, SPTBN1, PERP, GIGYF2			
GOTERM_BP_DIRECT	GO:0060021~palate development	TGFBR1, SMAD4, PKDCC, EPHB2, SUMO1, ACVR2B, CHD7, INSIG1, LRP6, PYGO2, ALX4, PLEKHA1, BMPR1A	1.965993011	0.03041812	1
GOTERM_BP_DIRECT	GO:0090200~positive regulation of release of cytochrome c from mitochondria	DNM1L, BBC3, TP53, HRK, PMAIP1, BMF, BCL2L11	2.873374401	0.03053564	1
GOTERM_BP_DIRECT	GO:0003281~ventricular septum development	LMO4, MDM2, LUZP1, DCTN5, LRP2, FRS2, CYR61	2.873374401	0.03053564	1
GOTERM_BP_DIRECT	GO:0071480~cellular response to gamma radiation	XRCC5, NOX4, ELK1, TSPYL5, ATM, RAD51	3.283856458	0.03094725	1
GOTERM_BP_DIRECT	GO:0045732~positive regulation of protein catabolic process	STX5, CDKN1B, PPP2R3A, ATG7, GSK3B, TRIM32, NDFIP1, NEDD4L, MYLIP, CUL4B, APC	2.107141227	0.03298193	1
GOTERM_BP_DIRECT	GO:0008344~adult locomotory behavior	SNCA, MECP2, PAFAH1B1, ADAM22, PBX3, SLITRK6, GDNF, CXCL12, GIGYF2, BTBD9	2.210288001	0.03376643	1
GOTERM_BP_DIRECT	GO:0030336~negative regulation of cell migration	RECK, CYP1B1, ADARB1, NF2, STK24, NF1, ABHD2, DACH1, ZMYND8, FUZ, MIA3, ROBO1, SRGAP3, STC1, TP53INP1	1.81476278	0.03487244	1
GOTERM_BP_DIRECT	GO:0016569~covalent chromatin modification	BMI1, AEBP2, BRD2, RSF1, RBL1, WBSCR22, CTCF, ARID1A, CBX6, CHD8, CHD7, MBTD1, BCORL1, MTF2, SMARCD1, CHD2, SMARCA5	1.729110259	0.03519678	1
GOTERM_BP_DIRECT	GO:0042953~lipoprotein transport	MIA3, ZDHHC17, PPARG, LRP2, PRKCB	3.831165868	0.03570189	1
GOTERM_BP_DIRECT	GO:0001953~negative regulation of cell-matrix adhesion	NF2, NF1, CASK, THBS1, PIK3R1	3.831165868	0.03570189	1
GOTERM_BP_DIRECT	GO:0048255~mRNA stabilization	PAIP1, HNRNPD, ELAVL1, AXIN2, GDNF	3.831165868	0.03570189	1
GOTERM_BP_DIRECT	GO:0000289~nuclear-transcribed mRNA poly(A) tail shortening	EIF4G1, PAN2, EIF4B, TNKS1BP1, EIF4E, PAIP1, CNOT10	2.774292525	0.03576265	1
GOTERM_BP_DIRECT	GO:0014066~regulation of phosphatidylinositol 3-kinase signaling	PPP1R16B, CD80, EREG, ERBB4, PIK3CB, ERBB2, GAB1, PDGFRA, PIP5K1B, KIT, NRG1, FRS2, PIK3R1	1.915582934	0.03635231	1
GOTERM_BP_DIRECT	GO:0035988~chondrocyte proliferation	STC1, MMP16, MMP14, HMGA2	5.108221158	0.03697997	1
GOTERM_BP_DIRECT	GO:0032464~positive regulation of protein homooligomerization	BBC3, HRK, BMF, BCL2L11	5.108221158	0.03697997	1
GOTERM_BP_DIRECT	GO:0010863~positive regulation of phospholipase C activity	PDGFRA, ESR1, KIT, ABL2	5.108221158	0.03697997	1
GOTERM_BP_DIRECT	GO:0060999~positive regulation of dendritic spine development	EIF4G2, CPEB3, FMR1, NRG1, ZMYND8, IL2	3.134590256	0.03726992	1
GOTERM_BP_DIRECT	GO:1904886~beta-catenin destruction complex disassembly	CSNK1A1, GSK3B, LRP6, FZD1, FRAT2, APC	3.134590256	0.03726992	1
GOTERM_BP_DIRECT	GO:0034113~heterotypic cell-cell adhesion	NRCAM, ITGAX, NFASC, ITGA4, ITGB3, PERP	3.134590256	0.03726992	1
GOTERM_BP_DIRECT	GO:0071456~cellular response to hypoxia [39,46]	SLC8A3, VASN, IRAK1, SLC8A1, HIF1A, BBC3, TP53, MDM2, STC1, MDM4, PMAIP1, PRKCE, PPARGC1A, KCNK2, SUV39H2	1.795859001	0.03768934	1
GOTERM_BP_DIRECT	GO:0071205~protein localization to juxtaparanode region of axon	CNTN2, NFASC, CNTNAP2	8.620123203	0.04025186	1
GOTERM_BP_DIRECT	GO:0045759~negative regulation of action potential	SUMO1, CNR1, CHRNB2	8.620123203	0.04025186	1
GOTERM_BP_DIRECT	GO:0030900~forebrain development	STIL, TWSG1, KDM2B, ARID1A, APAF1, LRP2, NR2F2, PPARGC1A, FRS2	2.248727792	0.04288581	1
GOTERM_BP_DIRECT	GO:0038128~ERBB2 signaling pathway	NRAS, EREG, ERBB4, ERBB2, GAB1, SHC1, NRG1, PIK3R1	2.419683706	0.04300692	1
GOTERM_BP_DIRECT	GO:2001237~negative regulation of extrinsic apoptotic signaling pathway	PHIP, CFLAR, CTTN, TGFBR1, SIAH2, SGMS1, THBS1, TCF7L2	2.419683706	0.04300692	1
GOTERM_BP_DIRECT	GO:0060291~long-term synaptic potentiation	SLC8A3, STX3, SNCA, MECP2, NPTN, RELN, ITPR3, SHANK2	2.419683706	0.04300692	1
GOTERM_BP_DIRECT	GO:0000381~regulation of alternative mRNA splicing, via spliceosome	SRSF6, SRSF12, SREK1, FMR1, DYRK1A, CELF3, RNPS1, WTAP	2.419683706	0.04300692	1
GOTERM_BP_DIRECT	GO:0009952~anterior/posterior pattern specification	KMT2A, TGFBR1, EMX2, GLI2, HOXC10, ACVR2B, CRKL, HIPK1,	1.867693361	0.04306158	1

		BTG2, HOXA5, CDON, ALX4, NR2F2			
GOTERM_BP_DIRECT	GO:0006511~ubiquitin-dependent protein catabolic process	USP40, NUB1, USP3, UBA6, SKP1, RLIM, ZNRF3, ARIH1, PSMF1, ATXN3, PSMC6, PSMD11, USP21, USP47, USP11, USP49, USP10, FBXO4, ITCH, CUL4B, SIAH2, USP34, TP53INP2, USP13	1.515626058	0.04415676	1
GOTERM_BP_DIRECT	GO:0006479~protein methylation	PRMT3, BTG2, GSPT1, PCMTD1, ETF1, HEMK1	2.998303723	0.04432716	1
GOTERM_BP_DIRECT	GO:0016239~positive regulation of macroautophagy	HIF1A, ATG7, RAB12, PRKAA2, KDR, LARP1	2.998303723	0.04432716	1
GOTERM_BP_DIRECT	GO:0006607~NLS-bearing protein import into nucleus	TRPS1, IPO5, KPNA6, RANBP2, KPNA2, KPNA1	2.998303723	0.04432716	1
GOTERM_BP_DIRECT	GO:0086002~cardiac muscle cell action potential involved in contraction	CACNA2D1, SCN1A, SCN2B, SCN3B, CACNA1G	3.591718001	0.04441992	1
GOTERM_BP_DIRECT	GO:0045956~positive regulation of calcium ion-dependent exocytosis	SYT1, SYT10, KCNB1, CACNA1G, SYT9	3.591718001	0.04441992	1
GOTERM_BP_DIRECT	GO:0030949~positive regulation of vascular endothelial growth factor receptor signaling pathway	GRB10, HIF1A, ITGB3, ARNT, PRKCB	3.591718001	0.04441992	1
GOTERM_BP_DIRECT	*GO:0080111~DNA demethylation [47]	TET3, FTO, AICDA, ALKBH1, TET2	3.591718001	0.04441992	1
GOTERM_BP_DIRECT	GO:0035855~megakaryocyte development	EP300, C6ORF25, KIT, ZNF385A, MED1	3.591718001	0.04441992	1
GOTERM_BP_DIRECT	GO:0060216~definitive hemopoiesis	ZFP36L2, KMT2A, HIPK1, TEK, CBFB	3.591718001	0.04441992	1
GOTERM_BP_DIRECT	GO:0048488~synaptic vesicle endocytosis	SYT1, SNCA, ITSN1, CANX, SH3GL2	3.591718001	0.04441992	1
GOTERM_BP_DIRECT	GO:0097194~execution phase of apoptosis	CFLAR, TAOK1, BBC3, STK24, CASP7	3.591718001	0.04441992	1
GOTERM_BP_DIRECT	GO:0042475~odontogenesis of dentin-containing tooth	ACVR2B, NF2, BCL11B, LRP6, GLI2, TRAF6, CTNNA1, RUNX2, BCL2L11, BMPR1A	2.089726837	0.04632134	1
GOTERM_BP_DIRECT	GO:0048147~negative regulation of fibroblast proliferation	NF1, TRIM32, TP53, FBXO4, DACH1, PMAIP1, TP53INP1	2.595305911	0.04785212	1
GOTERM_BP_DIRECT	GO:0070936~protein K48-linked ubiquitination	TRIM38, UBE2E3, SYVN1, RNF4, NEDD4L, ITCH, MARCH6, KLHL3, UBE2R2	2.20088252	0.04790403	1
GOTERM_BP_DIRECT	GO:0000165~MAPK cascade	ERBB4, CAMK2G, ERBB2, RASGEF1A, KIT, CCL5, GDNF, IL31RA, PSMB11, PEA15, PSMF1, GRIN2B, INS, TEK, DLG4, SHC1, NRG1, FRS2, NLK, NF1, PTPRA, NRAS, PSMC6, EREG, MAPK13, PSMD11, PDGFRA, SPTBN1, KSR1, CALM2, GFRA2, IL2	1.403785967	0.0485905	1
GOTERM_BP_DIRECT	GO:0048791~calcium ion-regulated exocytosis of neurotransmitter	SYT1, SYT10, SYT9, SYT13, SYT15, RPH3A, RIMS2, RIMS3	2.357640534	0.04863704	1
GOTERM_BP_DIRECT	GO:2000727~positive regulation of cardiac muscle cell differentiation	MYOCD, GSK3B, NRG1, FOXP1	4.597399042	0.04948845	1
GOTERM_BP_DIRECT	GO:0048280~vesicle fusion with Golgi apparatus	STX5, VTI1B, GOSR2, VTI1A	4.597399042	0.04948845	1
GOTERM_BP_DIRECT	GO:0061072~iris morphogenesis	HIF1A, HIPK1, PAX6, WNT2B	4.597399042	0.04948845	1
GOTERM_BP_DIRECT	GO:0060411~cardiac septum morphogenesis	CHD7, HEY2, MDM2, JAG1	4.597399042	0.04948845	1
GOTERM_BP_DIRECT	*GO:0042118~endothelial cell activation [48]	TGFBR1, SMAD4, APOLD1, FOXP1	4.597399042	0.04948845	1
GOTERM_BP_DIRECT	GO:0031115~negative regulation of microtubule polymerization	SNCA, STMN1, CLIP3, CAPZB	4.597399042	0.04948845	1
GOTERM_BP_DIRECT	GO:0060055~angiogenesis involved in wound healing	PIK3CB, ETS1, PRCP, ITGB3	4.597399042	0.04948845	1
GOTERM_BP_DIRECT	GO:0008593~regulation of Notch signaling pathway	IL6ST, CD46, SYNJ2BP, LFNG	4.597399042	0.04948845	1
GOTERM_BP_DIRECT	GO:0050884~neuromuscular process controlling posture	SCN1A, PRRT2, ATP8A2, GAA	4.597399042	0.04948845	1
GOTERM_BP_DIRECT	GO:2000009~negative regulation of protein localization to cell surface	GPM6B, LEPROT, NEDD4L, ASTN2	4.597399042	0.04948845	1
GOTERM_BP_DIRECT	GO:0071481~cellular response to X-ray	XRCC5, NIPBL, NUCKS1, ATM	4.597399042	0.04948845	1
GOTERM_CC_DIRECT	GO:0005634~nucleus	XRCC5, NAA15, CMTR2, SYNCRIP, RORB, SGMS1, ZNF253, WTAP, SART3, ZNF778, ZNF773, WDR77, PTBP3, DHX36, PTBP2, H1F0,	1.259321695	9.17E-12	6.49E-09

TNIK, PIK3CB, RREB1, ZNF506,
MECP2, ZHX2, ZNF791, ZNF649,
BSN, ESPL1, ZNF793, SKP1,
TOX3, ZNF37A, DCAF6, VGLL4,
RAD23B, PUS3, ERBB4, ERBB2,
ZNF232, ZNF619, MYBL1, ZNF514,
ZNF512, ZNF615, ZNF229, ARX,
CENPBD1, HEXIM1, DUSP16,
CASZ1, VEZF1, CCDC89, ZC3H14,
ZNF626, KCNK2, ATM, ZNF629,
WDR62, TRPS1, RGS6, RBMXL2,
MZF1, H3F3A, ZNF117, TAF1B,
ZNF518B, ZNF486, ZNF18,
ZNF532, ZNF155, NAP1L1,
ZNF676, ZNF12, NAP1L2, NAP1L5,
ZNF346, RLIM, ZNF14, ZNF737,
QKI, USP11, ZNF540, USP10,
BHLHE41, MYB, GATAD1, ZNF493,
ZNF33A, ZNF544, CNOT10,
ZDHHC7, TLE3, TP53, ZSWIM7,
CLIC2, RHOXF2B, OSTM1,
ZNF333, SRPK1, ZNF335, FLNA,
ZNF138, BRWD3, SS18, ZNF341,
BRWD1, CCND2, AICDA, ZNF550,
ZSCAN18, TMSB4Y, NSD1,
ZNF483, ARL4C, THOC1, ZNF555,
ZNF275, ZNF554, ZNF559, CREM,
ZNF367, ZNF35, AZIN1, ZNF655,
NUFIP2, ZNF652, ZNF30, FAN1,
GBX2, LIMD1, FBXW11, TRIP12,
USP40, BRD2, UPF1, ZNF568,
NUB1, NUCKS1, FADS1, ZFP1,
ZNF24, PPP1R11, ELAVL1, ZNF25,
DGKH, ZFP3, DGKI, APPL1, ETF1,
ZNF662, ZNF23, ZNF865,
ZSCAN30, ZNF362, GSK3B,
PTP4A1, SFPQ, POLDIP2, ZBTB4,
USP47, USP49, ZBTB2, ZNF257,
REPIN1, PRC1, XPO5, EIF5, STYX,
CBFB, IQGAP1, GTF2E1, ASPA,
MIER3, ZNF445, PMS1, ZNF449,
RSBN1L, STK24, MTA3, GABPA,
NUDT5, FMR1, RBL1, RBKS,
ARID1A, SIX4, GRHL2, WEE1,
SCCPDH, DCUN1D1, PA2G4,
EP300, KDM2B, HIF1A, TIMELESS,
ARRB1, SMARCA5, TESK2,
ZNF431, DCUN1D4, NMNAT1,
CAMTA1, PPIL2, CERS5, CERS3,
ZNF331, ARNT, SESN3, PEG10,
NIPBL, RNF165, OSBPL1A, NUMB,
HNRNPD, AUTS2, MBNL3, TRAF6,
RHOXF2, GINS1, CREBZF,
ARID3B, EHMT2, SUV39H2,
CDKN1C, ATF5, HNRNPH3,
CDKN1B, EBF3, ZNF317, ZIC5,
DYRK1A, E2F3, ZKSCAN8, RSF1,
CDC14A, GPBP1, DICER1, HR,
PAX6, PAX5, CTCF, ZKSCAN5,
RAB3IP, ZNF207, TRIM5, MAX,
CASP7, HEY2, S100PBP, NRG1,
INO80D, ZNF280B, ACADM, ZFX,
CDK8, ESR1, PKN2, CCNL1,
IRF2BP2, HMGA2, PURA,
TMEM38B, HOXC10, PPM1D,
TRNAU1AP, IPO5, CPSF7, CPSF6,
ZFPM2, LCOR, IRX5, KMT2A,
KMT2C, NFYB, NFYA, TSPYL5,
DRGX, HIC2, TSC22D3, CHD8,
CHD1L, ZNF512B, CHD7, REL,
FIGN, CHD2, MLXIP, NEDD4L,
NFATC2, NFATC3, CHD3,
ADARB1, NF2, CREB1, NF1, CBL,
RCAN1, KLK1, DACH1, EXO5,
FAM9C, SMC3, HSP90B1,
DENND4A, APAF1, SNCA, LUZP1,
SLC35A2, ZFP91, PICALM, ZFP90,
RARB, PAN2, EMX2, PPARGC1A,
PRDM7, PNKP, CAMK4, MTF1,
MTF2, PDGFRA, STC1, ANAPC7,

GOTERM_CC_DIRECT	GO:0005654~nucleoplasm	NEK2, GPBP1L1, TFCP2L1, SLC2A14, IGF2BP1, IGF2BP2, OAS1, OAS2, MYT1, ASB15, ASB18, HNRNPA3, TCF20, NGRN, NKX2-4, IKZF4, AEBP2, KLF7, IKZF3, MOCS2, KLF9, TP53BP2, KANSL3, FTO, SMAD4, RAD54L, HNRNPA0, KDR, TNKS1BP1, RNF4, ETS1, ETS2, JAZF1, ZFHX3, TCF12, ATAD2B, PCDHA2, KIAA0368, CPEB3, CCNT1, FERMT2, PPARG, TRMT10A, NFKB1, COPS8, ZEB1, GLI2, CAMKK1, ZBTB37, MBTD1, BAG1, MKX, NR2F2, IP6K1, ZBTB22, ZBTB20, POGZ, SLC25A4, PALD1, ZFP30, ZNF354B, MBD6, POLR1A, MBD5, FAM76B, MBD2, ZBTB26, GRM1, GTF2B, MBD1, ZCCHC17, STX1B, FOXR2, RAD51, SIAH2, TBX18, MED1, REV3L, USP3, FAM98B, THAP5, RPP14, STAT6, PHIP, ZFP36L2, SUMO1, SET, NR1D2, BCL11B, BCL11A, FAT2, BCL9L, PRKAA2, AGO4, FAM32A, ETV3, NEMF, RBM24, NLK, MEX3A, POLR3E, CAPRIN2, PSMD11, NEUROD6, PBX3, TP53INP2, CALM2, PGK2, TP53INP1, SEPT4, GRPEL1, ZC3HAV1, FGF14, TDRP, PMAIP1, FOXO4, CBX6, ZNF181, ZNF182, MYOCD, DERA, OLIG2, ITCH, ZNF180, AKIRIN1, GNL1, ZNF189, FOXN2, BASP1, FOXN3, RALGAPA1, FOPNL, TRIM32, CELF3, PYGO2, BIVM, CPD, DST, TSHZ3, TSHZ2, ZNF841, ONECUT3, ONECUT2, SOX5, ELK1, ITGB3, ZMYND8, WT1, ZNF846, PPP1R16B, MTCH2, HOXA5, HECTD2, SLC30A5, STRBP, AXIN2, NOVA2, RUNX2, NOVA1, CREBL2, MAFG, RFX8, LPP, RFX7, RNPS1, SUGT1, TET2, FOXP1, MSL3, UBE2E3, PSMC6, TET3, GCM2, PHF2, SPTBN1, RFX3, PLEKHA2, CDK19, BMI1, PRX, C9ORF72, METAP1, HECW2, NR6A1, DPY19L2, PDCD4, SYNPO2L, FOS, RANBP9, TUBA1A, ALX4, ZW10, IRAK2, SOX10, IRAK1, PARM1, EGR2, SOX11, DMRT3, UBE2F, PRKCE, UBN2, PRKCB, EIF4G1, EYA1, CTH, SBF1, HIPK1, LARP7, HIPK3, ZNF711, ZNF710, UBE2W, MDM2, KPNA6, MDM4, CUX2, KPNA2, MATR3, KPNA1, CAMK1D, GLIS3, FRK, MOB1B, ZBTB8B, NFIX, TCF7L2, PSMB11, RPA3, ZNF704, NFAT5, POU2F1, PHF20L1, ZNF700, PIK3R1, PPWD1, APC, TXNIP, ZBTB7A, HMBOX1, TMEM53, RACGAP1, GORAB, ATXN7L3, ATXN1, TOX, ATXN3, RPAP2, SP1, CASP14, CEP68, MYPN, SP5, URI1, GGNBP2, NFIB XRCC5, SNCB, SYNCRIP, INTS2, RORB, WTAP, SART3, MED20, GTF2IRD2B, PRIM1, NPAP1, MED29, WDR77, RARB, H1F0, TNIK, POLH, MAGI1, RREB1, ZHX2, NAA25, DCTN5, SKP1, PPARGC1A, CTDSPL2, PNKP, DCAF7, MTF1, CAMK4, MTF2, ANAPC7, RAD23B, ERBB4, GNAI2, SRSF12, TAF9B, TADA1, MYT1, HNRNPA3, TCF20, HEXIM1, DUSP16, SYBU, CASZ1, ZNF74,	1.282026626	1.56E-06	5.53E-04

GOTERM_CC_DIRECT	GO:0005737~cytoplasm	VEZF1, RPS23, ZC3H14, IKZF4, AEBP2, KLF9, TP53BP2, KANSL3, SREK1, SMAD4, RAD54L, ATM, HNRNPA0, FNIP2, TNKS1BP1, SRSF3, CCT4, RNF4, NSMCE4A, SRSF6, ETS1, TRPS1, ETS2, FNBP4, H3F3A, ZFHX3, ZNF385A, TAF1B, EVX2, EZH1, CCNT1, PPARG, FERMT2, ZNF12, NFKB1, ZEB1, COPS8, GLI2, RLIM, FANCM, DNAJC14, ZNF148, ANKRD12, TRMT6, ZNF540, USP10, DPP8, IP6K1, GATAD1, STX5, ZBTB20, POGZ, POLR1D, ZDHHC7, MBD6, POLR1A, TP53, TLE3, ANKHD1-EIF4EBP3, VEZT, MBD2, STX1B, GTF2B, RAD51, PDIK1L, SNRNP48, CCND2, USP21, SIAH2, NSD1, THOC1, MED1, REV3L, FAM98B, ZNF367, RPP14, STAT6, SUMO1, FAN1, SET, STK40, NR1D2, BCL11A, GATAD2B, BCL9L, SNAP23, PRKAA2, FAM32A, TRIP10, TRIP12, FOXD3, CWC25, UPF1, NLK, ELAVL1, DGKI, MAPK10, ANKHD1, ITPR3, POLR3E, CHML, BCORL1, PSMD11, SFPQ, CALM2, TP53INP1, SEPT4, PRC1, XPO5, FOXO4, CBX6, GTF2E1, AKIRIN1, STK24, FMR1, GABPA, MTA3, RBL1, ARID1A, GRHL2, WEE1, PA2G4, EP300, HIF1A, KDM2B, TIMELESS, ARRB1, SMARCA5, TESK2, PYGO2, ZNF436, NMNAT1, GPN1, TSHZ3, SYVN1, PPIL2, ONECUT2, ELK1, ENSA, WT1, ARNT, TRIM66, NIPBL, IVD, OSBPL1A, PRKRA, HNRNPD, ZNF420, PALB2, AXIN2, RUNX2, GINS1, MAFG, ARID3B, TEAD1, RNPS1, EHMT2, FOXP1, SUV39H2, MSL3, MSL2, ATF5, HNRNPH3, PSMC6, PHF2, CDKN1B, CDKN2AIP, DYRK1A, PLEKHA1, BMI1, NAMPT, C9ORF72, E2F3, RSF1, CDC14A, GPBP1, NR6A1, HR, PAX6, CTCF, PDCD4, LARP1, FOS, MAX, CASP7, HEY2, AKT3, CDK13, NIPAL4, PRKCA, SOX10, KHDRBS2, RUNX1T1, CDK8, ESR1, PKN2, UBN2, HMGA2, PRKCB, HOXC10, EYA1, HIPK1, LARP7, IPO5, CPSF7, CPSF6, MDM2, KPNA6, ZFPM2, MDM4, CPSF2, KPNA2, MATR3, KPNA1, PPP2R2A, GLIS3, KMT2A, KMT2C, CAMK2G, PPP3R1, WBSCR22, NFYB, NFYA, TCF7L2, RPA3, PSMF1, CHD8, MPPE1, CHD1L, REL, CHD2, POU2F1, NFAT5, PNPO, SLC39A7, NEDD4L, NFATC2, NFATC3, TNRC6A, APC, CHD3, ADARB1, SNX27, PM20D2, CREB1, EXO5, RACGAP1, GORAB, SMC3, ATXN1, ATXN3, RNF44, RPAP2, SP1, IRF2, CUL4B, SCP2 RBPMS2, NAA15, CMTR2, SART3, AMOTL1, MAP3K8, WDR77, ARHGEF40, DHX36, TNIK, NCF2, MAGI1, RREB1, ZHX2, UBE2J1, NAA25, ESPL1, SKP1, BTBD9, DCDC1, MARK1, GRB10, DCAF6, DCAF7, SIPA1L1, ROR1, ARL8A, SH3GL2, RAD23B, RALGPS2, PLCXD3, GNAI3, GNAI2, ERBB2, UBA6, SH3BP5L, ARIH1, DUSP13, KBTBD4, HEXIM1, DUSP16, CASZ1, CCDC89, CDC42EP4, SEC23IP, ARHGEF10L, RPS23,	1.179125927	3.02E-06	7.14E-04

SGIP1, ZC3H14, SEPT14, PAIP2,
ARHGEF38, TAOK1, PAIP1,
CCDC81, ATM, FNIP2, SRSF3,
AIDA, PTAR1, PKP4, APOL6,
ZNF385A, LRRC19, ZNF346,
LRRC15, RLIM, SLC23A2, MAPT,
USP11, ZNF540, QKI, USP10,
DPP8, CEP170B, SH3PXD2B,
FMNL3, SH3PXD2A, AIFM2, ACO1,
TP53, ERLIN2, PAPD5, CLIC2,
RFTN2, OSTM1, MMP14, SRPK1,
FLNA, CARD10, WDR47, MAST4,
BRWD1, KSR2, MTRF1, USP21,
SRGAP3, SERPINB2, USH1C,
AICDA, TMSB4Y, ARL4C, THOC1,
SNAP29, FGGY, CREM,
EPB41L4A, ABI2, AZIN1, PSAPL1,
ZNF655, ZNF365, NUFIP2, AMPH,
FAN1, LRRTM4, ANKS6, DGKG,
LIMD1, SNAP23, ACSL4, TRIP10,
TRIP12, USP40, BRD2, UPF1,
NUB1, NBPF10, NUCKS1,
PPP1R11, ELAVL1, DGKH, DGKI,
ANKHD1, APPL1, ETF1, TTC17,
CNTF, BCORL1, GSK3B, PTP4A1,
SFPQ, USP47, PRC1, XPO5,
AP1G1, IMPACT, EIF5, STYX,
IQGAP1, LNX1, EIF4EBP3,
GTF2E1, ASPA, ATAT1, ATG7,
AAK1, FRS2, STK24, MTA3, FMR1,
RBKS, SIX4, WEE1, DDIT4,
CCDC6, PA2G4, EP300, HIF1A,
ARRB1, PCMTD1, TESK2, RELN,
STMN1, RNF135, SLC40A1,
ZNF436, CAMTA1, PAFAH2, PPIL2,
CXXC4, NHS, CCL5, AZI2, ARNT,
SESN3, PEG10, RNF165,
OSBPL1A, PRKRA, TEK, ADRA2A,
MBNL3, TRAF6, GINS1, PHACTR4,
MYO1B, SHANK2, MPRIP,
GLYCTK, CDKN1C, ATF5, MYO10,
CDKN1B, PRKAR1A, CACNA1G,
E2F3, CDC14A, GPBP1, LRRC8B,
DEDD, DICER1, PAX6, ZNF207,
PRMT3, TRIM5, MAX, TRIM2,
DCAF12, CASP7, ZFYVE16, HEY2,
ALS2CR12, DLG4, NBPF3, NRG1,
CNTLN, SLA2, ESR1, PKN2,
IRF2BP2, IPO9, GAN, PURA,
TRNAU1AP, IPO5, ZFPM2, KMT2A,
UPP2, CDH2, UBE2R2, NDC1,
TSC22D3, MPPE1, CHD1L, FIGN,
PNPO, NEDD4L, NFATC2,
NFATC3, INPP5B, CHD3, WDFY3,
ALPK1, PDCD10, ADARB1, NF2,
SNX27, NF1, CBL, RCAN1, DACH1,
EXO5, SMC3, IFIT3, CUL4B,
RAB3GAP2, SNCB, VAPB, SNCA,
RNASEH1, GTF2IRD2B, ZNRF2,
GDF9, GLE1, RARB, POLH, VTI1B,
WNK3, UBR1, VASH1, CAMK4,
TXNDC8, MTF2, CCR2, PDGFRA,
STC1, LRCH3, KIF26B, NEK2,
TFCP2L1, C14ORF79, IGF2BP1,
IGF2BP2, AFAP1L2, OAS1, TADA1,
OAS2, MYT1, ASB15, CAPZB,
ASB18, FUZ, HNRNPA3, FBXO4,
CDC37L1, IKZF3, MOCS2, KLF9,
OSBPL9, TP53BP2, SMAD4,
TNKS1BP1, PMFBP1, CCT4,
SEMA6D, RNF4, ETS1, ETS2,
JAZF1, RGSL1, ZFHX3, TCF12,
CPEB3, FERMT2, NFKB1, TTLL6,
COPS8, ZEB1, TTLL1, KCNIP1,
CKB, BAG1, ANKRD12, TMSB15B,
IP6K1, POGZ, PCDHB4, POLR1A,
ANKHD1-EIF4EBP3, VEZT, MBD2,
MBD1, STX1B, RAD51, SNRNP48,
ZDHHC17, SGCD, SIAH2, CR1L,
MYO5A, RAB3B, USP3, FAM98B,

		LANCL1, TFG, LIX1, FKBP1A, RPP14, STAT6, PLCL2, ZFP36L2, DNALI1, SUMO1, FAM117B, SET, STK40, BCL11A, AGO3, PRKAA2, AGO4, DTNA, GDI1, RBM24, NOS1, FZD3, MAPK10, PTGFR, ITPR3, CAPRIN2, CHML, CALM2, PGK2, FAM126A, TP53INP1, CAST, STIL, ZC3HAV1, NIT2, TDRP, FAM179B, CASK, FOXO4, KLHL4, ROBO1, DERA, OLIG2, ITCH, C1ORF198, SOCS3, LYPLA1, BASP1, METTL7A, RALGAPA1, TRIM32, CELF3, BIVM, DYNLRB1, DST, GPN1, ZFAND5, PKHD1, SNX4, ENSA, ZMYND8, WT1, SBK1, HECTD2, ANKIB1, STRBP, AXIN2, RUNX2, STK38L, PRR9, CFLAR, ZMYM2, GLT1D1, LPP, RNPS1, RIMKLA, UBE2E3, KCTD9, TET3, EIF4E, PHF2, SLAIN2, PRICKLE2, SPTBN1, AHCYL1, CD79A, FAM84B, PLEKHA2, PLEKHA1, BMI1, PRX, NAMPT, C9ORF72, METAP1, HECW2, TLN2, PDCD4, LARP1, BZW1, CD96, CTTN, RANBP9, ACTR1A, SRD5A3, AKT3, CDK13, NIPAL4, ZW10, PRKCA, IRAK2, IRAK1, PARM1, EGR2, SOX11, RUNX1T1, UBE2F, PRKCE, EML6, PRKCB, EIF4G1, CTH, EYA1, ACVR2B, PANK3, HIPK1, LARP7, HIPK3, UBE2W, MDM2, KPNA6, NRK, KPNA2, KPNA1, CAMK1D, FRK, MOB1B, CYTH1, CD248, NANOS1, WBSCR22, TCF7L2, PSMB11, DDHD1, NFAT5, MSI2, YES1, PIK3R1, APC, TXNIP, DNM1L, MAP1B, HMBOX1, RACGAP1, GORAB, ATXN1, ATXN3, RNF44, ACTRT3, RPAP2, SP1, CASP14, CEP68, KCNN3, IRF2, URI1, SCP2, GGNBP2			
GOTERM_CC_DIRECT	GO:0005622~intracellular	ATP1B1, LTBP2, FGF14, RASGEF1A, INTS2, MYLIP, TRIM51, ITSN1, GDNF, RAB1A, IL31RA, ZNF778, ZNF181, FAM49A, GRIN2B, PGLYRP4, ZFP90, MAP3K9, ZNF773, TBC1D13, RALA, ZNF445, ZNF180, GTPBP1, PIK3CB, SOCS3, ZNF506, NUDT5, ZNF649, ZNF791, ZNF189, ZNF793, RPH3A, TBC1D22B, DCDC1, DDIT4, TRIM38, PITPNM2, RAB18, TRIM32, AKAP5, ZNF431, ARL8A, STMN1, SLC40A1, GLP1R, STYXL1, RALGPS2, ZNF841, ZNF619, RIMS2, ZNF514, ZNF331, ZNF615, ZNF846, SH3BP5L, ZNF229, B3GNT5, OSBPL1A, ZNF74, ZNF420, RHOBTB3, SMG9, GPR155, RAB8B, TGFBR1, KCNB1, SMAD4, ZNF626, PDZD8, HEATR3, ZNF317, ARF4, RHOT1, ZNF486, ZKSCAN8, ZNF155, ZNF676, ZNF12, ZKSCAN5, TRIM5, TRIM2, ZNF737, GOLGA1, ZNF540, USP10, SHC1, SH2D1B, KRBOX4, AKT3, ZNF493, SHC4, PRKCA, STX5, ZNF33A, STX3, ARHGEF7, ZNF544, ZFP30, ZNF354B, CLIC2, ZNF333, STX1B, PRKCB, ZNF138, MAST4, KSR2, EREG, CD80, RRAS2, IPO5, ZNF550, KSR1, ZNF483, FRK, ZNF559, ZNF655, PLCL2, ZNF30, DGKG, RAB11A, POM121C, NEDD4L, ZNF700, GDI1, PIRT, ZNF568, SNX27, NLK, PTPRA, ZFP1, NFASC, DGKH, RCAN1, DGKI, NPR3, MAPK10,	1.322030201	2.84E-04	0.05030245

		RACGAP1, ZNF662, CCDC68, ZNF23, NRAS, ARL17A, GSPT1, MAPK13, WSB2, GFRA2, ZNF257, IL2			
GOTERM_CC_DIRECT	GO:0030424~axon	NRP2, CCK, SNCA, DICER1, IGF2BP1, CANX, IQGAP1, IL31RA, EPHB2, ALCAM, ATAT1, KLC1, ROBO1, CNR1, MAPT, CNTNAP2, RAB11A, NEFM, DTNA, GABRG2, ACADM, CREB1, KCNB1, NF1, FMR1, BSN, FZD3, EIF4G2, CNTF, ADAM22, SLC38A1, DST, CHRNA10, HTR2A	1.810027927	0.00103678	0.1174061
GOTERM_CC_DIRECT	GO:0043025~neuronal cell body	MYO5A, SCN1A, PARD3, AGFG1, IL6ST, ADORA2A, ELK1, GRIN3A, CANX, EPHB2, ALCAM, PICALM, GPM6A, KLC1, CNTNAP2, PAFAH1B1, PTBP2, ACSL4, ARHGEF7, VTI1B, FZD3, DGKI, PSD2, ITPR3, PDYN, PPARGC1A, SHANK2, KCNK2, FLNA, VTI1A, PURA, EIF4B, MYO10, KCNN3, GSK3B, CCR2, CNTN2, LRP6, SORT1, SIAH2, ZNF385A, BMPR1A, CACNA1B, HTR2A	1.650826591	0.00120846	0.1174061
GOTERM_CC_DIRECT	GO:0043194~axon initial segment	NRCAM, SCN1A, KCNQ3, CCK, ANK3, NFASC	6.446409622	0.00128557	0.1174061
GOTERM_CC_DIRECT	GO:0005829~cytosol	XRCC5, DYNC1LI2, VPS54, SNCA, VPS53, PPCS, MYLIP, AMOTL1, ITSN1, RAB1A, ZNRF2, PGPEP1, WDR77, GAB1, MAP3K8, DHX36, NWD1, PAN2, GTPBP1, NCF2, PIK3CB, MECP2, VTI1B, ESPL1, DCTN5, WNK3, UBR1, SKP1, CTNNA1, VTI1A, BCL2L11, GRB10, RAB18, CAMK4, CCR2, ANAPC7, SH3GL2, ADD2, ERBB4, GNAI2, NEK2, ARHGEF28, ASAP1, IGF2BP1, UBA6, IGF2BP2, OAS1, CACNB3, OAS2, CACNB4, CAPZB, EPHB2, PEA15, ARIH1, GOLPH3L, DUSP16, FBXO4, AMD1, SEC23IP, RPS23, ARHGEF10L, MOCS2, OSBPL3, TAOK1, PAIP1, SMAD4, TNNI1, DENND1B, TNKS1BP1, CCT4, GBE1, ARF4, RGS6, GOSR2, GOSR1, C19ORF12, PHKB, PPARG, FERMT2, NFKB1, GLI2, RLIM, CAMKK1, CKB, BAG1, MAPT, GOLGA1, FRAT2, PIK3AP1, IP6K1, SH2D1B, STX5, FMNL3, AIFM2, PALD1, POLR1D, ACO1, ARHGEF7, CNOT10, TP53, OSTM1, STX1B, WAS, FLNA, CCND2, SRGAP3, USH1C, SORT1, SIAH2, TMSB4Y, KSR1, EIF5A2, MYO5A, MIDN, PPP6C, PARD3, RAB3B, RABGAP1, UROS, ABI2, TFG, FKBP1A, AZIN1, STAT6, ZFP36L2, SET, PDE1C, KLC1, WIPF2, PREPL, RAB11A, AGO3, AGO4, PRKAA2, TRIP10, USP34, FBXW11, TRIP12, PHLDA1, CSNK1A1, GDI1, NOS1, UPF1, MAT2A, VTA1, NLK, ELAVL1, DGKI, MAPK10, APPL1, ETF1, POLR3E, YWHAG, CHML, MAPK13, PSMD11, GSK3B, IST1, TP53INP2, CALM2, HTR2A, TP53INP1, FAM126A, CAST, STIL, CHMP4C, PRC1, XPO5, AP1G1, EIF5, STYX, RASGEF1A, CASK, PMAIP1, FOXO4, KLHL3, IQGAP1, ASPA, ATAT1, ANK3, ATG7, TBC1D14, SPRED2, DERA, DNAJC6, ITCH, FNDC3A, SOCS3, STK24, NUDT5, LYPLA1, PDE4C, RPH3A, DDIT4, TRIM38, HIF1A, ARRB1, NBR1, TRIM32, AKAP5, STMN1, CLIP3, RNF135, DST, TMEM216, TCP10,	1.16223353	0.00140705	0.1174061

		PRKRA, HNRNPD, HECTD2, EIF3J, TRAF6, AXIN2, BMF, EIF3M, RHOBTB3, SMG9, CFLAR, ZMYM2, RNPS1, SUGT1, EIF4B, GLYCTK, PSMC6, MYO10, EIF4E, CDKN1B, BBC3, PDE7A, PRKAR1A, RHOT1, SPTBN1, AHCYL1, ABL2, CDK19, NAMPT, METAP1, SEC24B, KIF24, DICER1, BORA, PPIP5K2, PIP5K1B, WWC2, PDCD4, RAB3IP, PRMT3, FOS, TRIM5, RANBP9, ACTR1A, CASP7, SHC1, RANBP2, TUBA1A, ZW10, IRAK2, PRKCA, IRAK1, RSU1, PKN2, IPO9, KIF16B, PRKCE, PGM2L1, PRKCB, EIF4G1, EIF4G2, CTH, CRKL, BTG2, MDM2, KPNA6, AKR1D1, KPNA2, KPNA1, PPP2R2A, MOB1B, TNFRSF25, CYTH1, CAMK2G, PPP3R1, UPP2, FAM13A, SEC62, TPM3, PSMB11, PSMF1, TSC22D3, DDHD1, TNRC6C, REL, PNPO, PAFAH1B1, BRK1, NEDD4L, NFATC2, TNRC6B, YES1, NFATC3, TNRC6A, INPP5B, PIK3R1, APC, DTX4, TXNIP, DNM1L, PDCD10, GIMAP6, SNX27, NF1, CBL, MAP1B, RACGAP1, EXO5, TAB2, SMC3, MON2, USP6NL, IFIT3, HSP90B1, ATXN3, GSPT1, NEB, IRF2, CUL4B, APAF1			
GOTERM_CC_DIRECT	GO:0005794~Golgi apparatus	SYT1, ZC3HAV1, LDLR, SNCB, AP1G1, VAPB, C6ORF25, VPS54, SNCA, PPARG, VPS53, SLC35A2, RAB1A, AQP2, CTTN, ATAT1, PICALM, ZNF148, ANK3, TBC1D14, CD46, WDR77, ST3GAL6, GOLGA1, CNTNAP2, LEPROT, FNDC3A, AKT3, CDK13, H1F0, STX5, PARM1, ACO1, MDGA1, ZDHHC7, ESR1, NAA25, VTI1B, DYNLT1, PKDCC, PRKCE, CTNNA1, RPH3A, VTI1A, LRPAP1, KIF1C, ZDHHC17, TXNDC8, LARP7, ZDHHC13, IPO5, SORT1, ADAM19, PTGFRN, EXT1, TMED7-TICAM2, MYO5A, GLIS3, GALNT1, GNAI3, FUT9, GALNT6, ST8SIA1, TMED7, MPPE1, ZDHHC22, OPALIN, SLC35B4, KBTBD8, SLC30A5, PREPL, SLC39A7, RAB11A, FUT1, YES1, AXIN2, INPP5B, TNRC6A, RNF144A, GDI1, ST6GAL1, DNM1L, PDCD10, OSBPL9, GORAB, KDR, NRAS, ARL17A, CXCL14, ARF4, TMEM165, LRP6, ATP8A2, POFUT2, GOSR2, GOSR1, LRP2, PERP, ATP8A1	1.355762858	0.0015518	0.1174061
GOTERM_CC_DIRECT	GO:0030426~growth cone	ORAI2, MYO5A, TSHZ3, STX3, FMR1, CBL, DICER1, SNCA, IGF2BP1, BASP1, SHANK2, IQGAP1, CTTN, KLC1, GSK3B, CNR1, MAPT, TOR1A, PAFAH1B1, PTBP2, CALM2	2.139541124	0.00165828	0.1174061
GOTERM_CC_DIRECT	GO:0030054~cell junction	PRX, SLC8A3, NAMPT, SYT1, CPEB3, SNCA, DUOX2, GRIK5, SYT9, GRIN3A, AMOTL1, ITSN1, IL31RA, ZNRF2, GRIN2B, PRRT2, DLG4, SV2B, DLG2, GRID1, SHC4, SH3PXD2B, GABRG2, SH3PXD2A, MAGI1, CACNG8, PTPRN2, FMR1, PKN2, BSN, BASP1, CTNNA1, RPH3A, ZDHHC17, SIPA1L1, PARD3, RIMS2, GSG1L, RIMS3, AMPH, LRRTM4, TOR1A, DTNA, LRFN2, GABRA1, DNM1L, GABRA4, DLGAP2, KCNB1, MAP1B, SHANK2, KDR, TMEM47, CEP68, PKP4, CHRNB2, PERP, CHRNA10	1.493394822	0.00212384	0.1366979

GOTERM_CC_DIRECT	GO:0005912~adherens junction	CD96, TMEM47, NF2, MAGI1, WNK3, VEZT, MPP7, LIMD1, AJAP1, JAG1, CTNNA1, APC	2.836420233	0.00260816	0.15388139
GOTERM_CC_DIRECT	GO:0000790~nuclear chromatin	USP3, PAX6, TCF7L2, STAT6, GATAD2B, NFATC2, RUNX2, ETV3, FOXD3, H1F0, PHOX2B, POGZ, NUCKS1, CREB1, GABPA, TP53, SMAD4, ESR1, ARID1A, MBD2, EHMT2, MBD1, RAD51, SP1, TIMELESS, TRPS1, RFX3, ZNF385A, TCF12	1.775824412	0.00340803	0.18560662
GOTERM_CC_DIRECT	GO:0016281~eukaryotic translation initiation factor 4F complex	EIF4G1, EIF4EBP3, EIF4B, EIF4G2, EIF4E	6.565787577	0.00453608	0.22939624
GOTERM_CC_DIRECT	GO:0031410~cytoplasmic vesicle	SNAP29, C9ORF72, SLC9A6, AGFG1, KIAA0368, ERBB2, CXXC4, AMOTL1, STARD4, KLC1, DUSP16, SYBU, RAB11A, SH3TC2, PHLDA1, ATG9B, FLOT2, MMP14, ATM, USP6NL, ARRB1, NBR1, IST1, LRP6, SORT1, RAB12, DST, GGNBP2, TP53INP2, ADD2, TP53INP1, HTR2A	1.609316445	0.00882616	0.40734149
GOTERM_CC_DIRECT	GO:0043197~dendritic spine	SLC8A3, SLC8A1, NOS1, FMR1, MAP1B, IGF2BP1, ASAP1, DGKI, CANX, ZMYND8, SHANK2, CTTN, GPM6A, ARRB1, SIPA1L1, ARF4, DLG4	2.009130999	0.00956245	0.40734149
GOTERM_CC_DIRECT	GO:1990909~Wnt signalosome	WNT3, GSK3B, LRP6, FZD1, APC	5.372008018	0.01035614	0.40734149
GOTERM_CC_DIRECT	GO:0016442~RISC complex	EIF4E, DICER1, AGO3, LIMD1, AGO4	5.372008018	0.01035614	0.40734149
GOTERM_CC_DIRECT	GO:0031201~SNARE complex	SNAP29, STX5, SYT1, STX3, VTI1B, GOSR2, SNX4, GOSR1, SNAP23, STX1B, VTI1A	2.452879133	0.01231103	0.45874771
GOTERM_CC_DIRECT	GO:0042734~presynaptic membrane	SYT1, ZDHHC17, PICALM, STX3, ADORA2A, FMR1, GRIK5, NPTN, CASK, RIMS2, ZNRF2, IL31RA	2.287435672	0.01399901	0.47306246
GOTERM_CC_DIRECT	GO:0032433~filopodium tip	MYO5A, MYO10, OSBPL3, FMR1, FZD3	4.924340683	0.01450869	0.47306246
GOTERM_CC_DIRECT	GO:0032993~protein-DNA complex	EYA1, SP1, NFYB, NFYA, HMGA2, TCF7L2, MED1	3.309156939	0.01579966	0.47306246
GOTERM_CC_DIRECT	GO:0005905~clathrin-coated pit	CTTN, PICALM, ATAT1, DNM1L, LDLR, ARRB1, AAK1, SORT1, LRP2, ITSN1, SGIP1	2.363683528	0.01584822	0.47306246
GOTERM_CC_DIRECT	GO:0016020~membrane	XRCC5, ATP1B1, DYNC1LI2, TMEM19, IL6ST, SNCA, NAA15, DNAJB14, SYNCRIP, VPS53, INTS2, LUZP1, SGMS1, GRIN3A, AQP2, PRIM1, PICALM, AGPS, PRRT2, GLE1, RECK, GTPBP1, ABCB11, MDGA1, PNKP, MGAT3, PDGFRA, ARL8A, GIGYF2, ORAI2, GNAI3, GNAI2, ERBB2, TFCP2L1, ASAP1, CACNB3, OAS2, CAPZB, SORCS3, SERINC4, LFNG, RPS23, SPPL3, OSBPL3, TGFBR1, ATP2A2, AIDA, ARF4, GOSR2, GOSR1, NRP2, KCNJ16, KIAA0368, LDLR, NAP1L1, GLI2, CANX, FLT3LG, DNAJC14, SLC23A2, PIK3AP1, ZFYVE1, STX5, FLOT2, CNOT10, STX1B, FLNA, KSR2, ZDHHC13, CHSY1, KSR1, GRAMD1B, CR1L, MED1, MYO5A, FKBP1A, KIT, NUFIP2, KLC1, FMO2, RAB11A, AGO3, FUT1, VMP1, AGO4, ACSL4, CSNK1A1, SLC8A1, XKR4, FADS1, ELAVL1, ITPR3, MARCH6, RAB32, NRAS, YWHAG, SLC16A6, PSMD11, KREMEN1, CMTM6, CAST, AP1G1, JAG1, CBFB, EIF4EBP3, RNF141, PGLYRP4, SPRED2, ITCH, PARD3B, FRS2, DDOST, FNDC3A, STK24, FMR1, RPH3A, METTL7A, SCCPDH, PA2G4, IGSF1, NBR1, PCMTD1, LCLAT1, KDSR, CPD, STMN1, DYNLRB1, PLXNC1, SYVN1, MAMDC2, ADORA2A,	1.149609716	0.01603602	0.47306246

		ADCYAP1R1, SNX4, MIA3, ITGAX, MTCH2, PRKRA, B3GNT6, SLC30A5, ENTPD1, STK38L, ICMT, ITGA4, MUC4, PDZD8, PSMC6, DIO2, PRKAR1A, RHOT1, SYT15, PLEKHA2, DDX51, SEC24B, KIAA0430, PIP5K1B, TMEM237, LARP1, BZW1, FOS, PIGG, CNTNAP2, SV2B, RANBP2, NRG1, DENND5B, SLC1A1, LBR, DLG2, NIPAL4, ZW10, PCYOX1L, ESR1, MPP6, IPO9, SLC7A11, TMEM38B, EIF4G1, EIF4G2, RRAS2, IPO5, CPSF7, GAA, CPSF6, KPNA6, KPNA2, CPSF2, MATR3, CALCR, GALNT1, CAMK2G, CDH2, ESYT2, SEC62, NDC1, PSMF1, CCDC126, TOR1A, BCAP29, PALM2, SLC39A7, CD4, EHD1, INPP5B, PIK3R1, SLC39A1, PLP2, DNM1L, NF2, NF1, CYP20A1, HSP90B1, CHRNA10, SCP2, ATP8A1			
GOTERM_CC_DIRECT	GO:0005783~endoplasmic reticulum	CAST, KIAA0368, PCDHA2, ATP10B, VAPA, VAPB, C6ORF25, GRIK5, SYNCRIP, PDIA6, PCDHA1, SGMS1, SLC35A2, CANX, FOS, PIGG, ANK3, ELOVL3, INSIG1, SRD5A3, DLG4, DDOST, ZFYVE1, ZW10, PRKCA, ACO1, TP53, ERLIN2, METTL7A, CDS1, PRKCE, SRPK1, VASH1, LRPAP1, PNPLA7, KIF1C, ZDHHC13, RRAS2, KDSR, KSR1, EXT1, TMED7-TICAM2, MYO5A, FKBP9, SYVN1, MAMDC2, CERS5, OAS1, CERS3, OAS2, SEC62, PSMF1, TMED7, MPPE1, ZDHHC22, FNDC5, SET, TOR1A, SLC35B4, BCAP29, POU2F1, SLC39A7, VMP1, MR1, THBS1, SEC23IP, TRAM1, PLP2, ABCB9, DNM1L, ICMT, HS1BP3, ITPR3, KCNK2, KDR, RAB32, HSP90B1, ATP2A2, BBC3, CNIH4, PTP4A1, LRP6, FAF2, TMTC2, C19ORF12, LRP2, TBC1D20, ATP8A1	1.25606371	0.01977346	0.5537179
GOTERM_CC_DIRECT	GO:0043235~receptor complex	ERBB4, LDLR, ADCYAP1R1, PTPRN2, ERBB2, TGFBR1, PTPRA, LIFR, PIGR, ITGB3, ITPR3, CAPRIN2, ACVR2B, ADRA2A, ROR1, LRP6, LRP2, SLITRK5, CR1L	1.768109726	0.02066658	0.5537179
GOTERM_CC_DIRECT	GO:0005875~microtubule associated complex	PEA15, RANBP9, RABGAP1, ACTR1A, MAPT, MAP1B, PAFAH1B1, RANBP2	2.78080415	0.02160619	0.5537179
GOTERM_CC_DIRECT	GO:0014069~postsynaptic density	ADORA2A, CPEB3, CAMK2G, GRIK5, GRIN3A, SORCS3, MAPT, DLG4, DNAJC6, AXIN2, DLG2, DLGAP2, CACNG8, FMR1, MAP1B, BSN, GRM1, SHANK2, ARRB1, GSK3B, SIPA1L1, PKP4, NPTN, SPTBN1, ADD2	1.605763266	0.02199117	0.5537179
GOTERM_CC_DIRECT	GO:0019005~SCF ubiquitin ligase complex	ARIH1, KBTBD4, FBXO27, USP47, FBXL5, FBXL22, FBXO4, SKP1, FBXW11, BTBD9	2.363683528	0.02288103	0.5537179
GOTERM_CC_DIRECT	GO:0016023~cytoplasmic, membrane-bounded vesicle	NTF3, KIAA0368, AGFG1, SLA2, ATM, KDR, USP6NL, STARD4, KLC1, IST1, NUMB, DUSP16, SYBU, SORT1, AXIN2, DST, GGNBP2, ADD2, HTR2A, PHLDA1	1.712814151	0.02346262	0.5537179
GOTERM_CC_DIRECT	GO:0044233~ER-mitochondrion membrane contact site	RAB32, ACSL4, CANX, ZFYVE1	5.90920882	0.02444598	0.55831471
GOTERM_CC_DIRECT	GO:0031234~extrinsic component of cytoplasmic side of plasma membrane	FRK, ATP2A2, CYTH1, FERMT2, DLG4, RACGAP1, YES1, ESYT2, ABL2, KCNIP1, IQGAP1, DTNA	2.085603113	0.02663369	0.58927035
GOTERM_CC_DIRECT	GO:0005769~early endosome	MYO5A, LDLR, KIAA0368, ASTN2, ST8SIA2, RAB1A, GRIPAP1, ZFYVE16, NUMB, CNTNAP2, USP10, CD4, EHD1, PARM1, ABCB9, NF2, MYO1B, SNX27, ACKR2, KIF16B, KDR, RND2,	1.496655509	0.03069789	0.6207643

		RAB32, PTP4A1, LRP6, SORT1, KIAA0319, SIAH2, TMED7-TICAM2			
GOTERM_CC_DIRECT	GO:0005891~voltage-gated calcium channel complex	CACNA2D1, CACNG8, CACNA1G, CACNB3, CACNB4, CACNA1B, CACNA2D4	2.852721499	0.0317661	0.6207643
GOTERM_CC_DIRECT	GO:0045202~synapse	GABRA1, SLC9A6, NOS1, SNCB, TLN2, CPEB3, FMR1, DGKI, GRIN3A, CACNB4, CDH2, ITSN1, RPH3A, NRCAM, SUMO1, PRRT2, LRP6, CNTN2, DLG4, USH1C, MDM2, DNAJC6, SH3GL2, DTNA	1.567083002	0.03205741	0.6207643
GOTERM_CC_DIRECT	GO:0033268~node of Ranvier	SCN1A, KCNQ3, ANK3, CNTN2, NFASC	3.939472546	0.03264378	0.6207643
GOTERM_CC_DIRECT	GO:0005868~cytoplasmic dynein complex	DYNC1LI2, SNX4, DYNLT1, DYNLRB1, BCL2L11	3.939472546	0.03264378	0.6207643
GOTERM_CC_DIRECT	GO:0008076~voltage-gated potassium channel complex	KCNJ16, KCNB1, KCNK2, KCNIP1, KCNQ5, CTTN, KCNQ4, SUMO1, KCNQ3, DLG4, CNTN2, CNTNAP2, CALM2, DLG2	1.880202806	0.03331786	0.6207643
GOTERM_CC_DIRECT	GO:0005913~cell-cell adherens junction	CAST, VAPA, ZC3HAV1, VAPB, EIF5, ASAP1, CDH2, ESYT2, CAPZB, RAB1A, IQGAP1, LARP1, BZW1, CTTN, PICALM, FAT2, NUMB, EHD1, APC, VASN, STX5, MYO1B, STK24, FLOT2, CBL, PKN2, MPP7, CTNNA1, FLNA, MPRIP, EIF4G1, TNKS1BP1, EIF4G2, CRKL, UBFD1, IST1, SPTBN1, GIGYF2	1.390402075	0.03622814	0.64142509
GOTERM_CC_DIRECT	GO:0005667~transcription factor complex	E2F3, LMO4, ONECUT3, CREM, NR6A1, NAA15, ZEB1, ARNT, FOS, REL, LIMD1, ALX4, RUNX2, RBL1, SMAD4, DACH1, ATF5, HIF1A, EP300, ETS1, TRPS1, RFX3, PBX3, ZFHX3, TCF12	1.530883114	0.03623871	0.64142509
GOTERM_CC_DIRECT	GO:0005643~nuclear pore	NDC1, SUMO1, AGFG1, IPO5, KPNA6, PARP11, POM121C, GLE1, RANBP2, KPNA2, EIF5A2, KPNA1	1.969736273	0.03868524	0.65414044
GOTERM_CC_DIRECT	GO:0030425~dendrite	SLC9A6, CCK, GNAI2, IL6ST, ADORA2A, CPEB3, DICER1, GRIK5, ELK1, KCNIP1, EPHB2, ALCAM, MAX, SUMO1, ANK3, MAPT, CNTNAP2, NRG1, STX3, KCNB1, NF1, FMR1, BSN, FZD3, PSD2, PDYN, GRM1, PURA, EIF4B, CCR2, NPTN, SORT1, RELN, URI1, ZNF385A, KPNA1, BMPR1A, CACNA1B, HTR2A	1.375875486	0.03880494	0.65414044
GOTERM_CC_DIRECT	GO:0005884~actin filament	MYO5A, CTTN, MYO1B, TEK, WIPF2, ACKR2, YES1, CAPZB, FLNA, IQGAP1, TPM3	2.000039908	0.04541623	0.70632432
GOTERM_CC_DIRECT	GO:0008021~synaptic vesicle	SYT1, RAB3B, STX3, SNCA, VTI1B, DGKI, RPH3A, STX1B, VTI1A, AMPH, TOR1A, DLG4, SV2B, SLC40A1	1.798454858	0.0455019	0.70632432
GOTERM_CC_DIRECT	GO:0044224~juxtaparanode region of axon	DLG4, CNTN2, CNTNAP2, DLG2	4.727367056	0.04611944	0.70632432
GOTERM_CC_DIRECT	GO:0005856~cytoskeleton	SEPT4, PARD3, PRC1, TMEM216, FERMT2, EPB41L4A, ABI2, IGF2BP2, MYLIP, KLHL4, CAPZB, KLHL3, RAB3IP, TPM3, FUZ, CTTN, TOR1A, TEK, SYBU, PREPL, BRK1, TRIP10, SEPT14, TNIK, NOS1, NF2, LDB3, GAN, BASP1, PRKCE, SLC7A11, MARK1, TNKS1BP1, CCDC6, ACTRT3, SIPA1L1, PKP4, AKAP5, USH1C, SGCD, DST, ADD2	1.337934072	0.0465764	0.70632432
GOTERM_CC_DIRECT	GO:0005789~endoplasmic reticulum membrane	SLC8A3, RAB3GAP2, SEC31B, RFT1, SEC24B, SLC9A6, ATP10B, VAPA, LRRC8B, VAPB, DNAJB14, PDIA6, CANX, RAB1A, DNAJC14, PIGG, ELOVL3, INSIG1, SRD5A3, SCD5, DDOST, ZW10, STX5, SPTLC3, FAXDC2, UBE2J1, ERLIN2, VTI1B, CDS1, VTI1A, CYP4X1, RAB18, FKBP14, LCLAT1, SORT1, KDSR, PTGFRN, KSR1, EXT1, UGT2B15, EIF5A2, DST, TMED7-TICAM2, FKBP9, GALNT1, CYP1B1, SYVN1, FAM69A, CERS5,	1.206520594	0.04688876	0.70632432

		FKBP1A, CERS3, FKBP1C, SEC62, TMCC1, GJC1, TMED7, MIA3, FMO2, BCAP29, SLC39A7, CD4, POM121C, MR1, ACSL4, TRAM1, SLC39A1, NOX4, ACER3, PLP2, DNM1L, OSBPL3, FADS1, CYP2C8, ICMT, ITPR3, KCNK2, HSP90B1, ATXN3, ATP2A2, GOSR2, POFUT2, AHCYL1, SVIP, TMTC2, RDH16, C19ORF12, CYP8B1, TBC1D20			
GOTERM_CC_DIRECT	GO:0005811~lipid particle	IRAK1, SCCPDH, AIFM2, ALDH3B2, FAF2, HILPDA, ACSL4, TRAF6, EHD1, METTL7A, REPIN1	1.969736273	0.04964222	0.7322228
GOTERM_MF_DIRECT	GO:0003700~transcription factor activity, sequence-specific DNA binding	LMO4, RORB, FOXO4, CBFB, ZNF182, ZNF773, ZNF445, ZNF449, RREB1, MTA3, GABPA, MECP2, ZHX2, ZNF649, ZNF189, FOXN2, ZNF793, GRHL2, FOXN3, ZNF37A, PA2G4, HIF1A, MTF1, ZNF436, ZNF841, GPBP1L1, TFCP2L1, SOX5, ZNF232, ELK1, ZNF514, MYT1, ZNF615, WT1, ARNT, ZNF229, TCF20, HOXA5, ZNF74, ZNF420, RUNX2, CREBL2, RFX8, MAFG, IKZF4, KLF7, IKZF3, CREBZF, KLF9, RFX7, SMAD4, TEAD1, FOXP1, ZNF629, ATF5, RNF4, ETS1, ZNF317, ETS2, TRPS1, MZF1, RFX3, ZNF117, ZFHX3, TCF12, TAF1B, E2F3, ZNF18, ZKSCAN8, GPBP1, ZNF532, NR6A1, PPARG, HR, PAX6, ZNF155, ZNF12, NFKB1, CTCF, ZEB1, GLI2, ZKSCAN5, ZNF207, FOS, MAX, HEY2, ZNF540, NR2F2, GATAD1, ZNF493, ZNF33A, EGR2, ZNF544, ZFP30, DMRT3, SOX11, ZNF354B, ZFX, RUNX1T1, TP53, ESR1, PURA, ZNF138, ZNF341, ZNF711, ZSCAN18, TBX18, ZNF483, ZNF275, KMT2A, CREM, ZNF367, NFYB, NFIX, ZNF35, NFYA, ZNF655, TCF7L2, STAT6, ZNF30, ZFP36L2, TSC22D3, REL, NR1D2, GBX2, NFAT5, POU2F1, GATAD2B, MLXIP, NFATC2, NFATC3, FOXD3, ZNF568, CREB1, CBL, ZFP1, ZNF24, RCAN1, ZFP3, ZNF23, SP1, ZSCAN30, IRF2, PBX3, NFIB	1.763906624	9.97E-12	1.23E-08
GOTERM_MF_DIRECT	GO:0003677~DNA binding	XRCC5, GTF2IRD2B, ZNF778, ZFP90, RARB, ZHX2, MECP2, ZNF791, PPARGC1A, ZNF37A, MTF1, MTF2, GPBP1L1, ZNF619, MYBL1, MYT1, ZNF514, ZNF512, ZNF615, ZNF229, TCF20, CENPBD1, CASZ1, ZNF74, VEZF1, KLF7, KLF9, SMAD4, RAD54L, ATM, ZNF629, RNF4, ETS1, ETS2, H3F3A, ZNF385A, ZNF18, ZNF518B, PPARG, ZMAT2, CCNT1, ZNF155, ZNF12, NFKB1, ZEB1, ZBTB37, ZNF14, FANCM, ZNF540, MYB, NR2F2, BHLHE41, ZNF493, ZBTB22, ZBTB20, ZNF33A, POGZ, AIFM2, POLR1D, ZFP30, ZNF544, ZNF354B, MBD6, TP53, POLR1A, MBD5, PAPD5, ZNF333, ZBTB26, MBD2, MBD1, RAD51, ZNF341, ZNF550, ZSCAN18, TBX18, ZNF483, REV3L, THOC1, THAP8, ZNF555, ZNF275, ZNF554, RABGAP1, ZNF559, CREM, THAP5, ZNF367, ABI2, ZNF35, ZNF655, ZNF652, STAT6, ZNF30, ZFP36L2, FAN1, SET, UPF1, ZNF568, ZNF24, ZFP1, ZNF25, ZFP3, ZNF662, ZNF23, ZNF865, ZNF362, POLDIP2, ZBTB2, PBX3, NEUROD6, REPIN1, FOXO4,	1.522411362	8.60E-11	5.32E-08

		CBFB, ZNF181, ZNF182, RNF141, MIER3, OLIG2, ZNF445, ZNF180, PMS1, MTA3, GABPA, ZNF189, ARID1A, PA2G4, KDM2B, EP300, SMARCA5, ZNF436, TSHZ3, ZFAND5, TSHZ2, AGFG1, ZNF841, ONECUT3, SOX5, CERS5, CXXC4, CERS3, ARNT, ZNF846, PEG10, HOXA5, STRBP, PALB2, ZNF420, RUNX2, RFX8, ZMYM2, CREBZF, RFX7, TEAD1, ARID3B, TET2, MSL3, TET3, GCM2, ZNF317, EBF3, ZIC5, RFX3, E2F3, GPBP1, DEDD, NR6A1, HR, PAX6, PAX5, ZKSCAN5, ZNF207, FOS, HEY2, ALX4, LBR, HEMK1, ZNF280B, EGR2, ZFX, ESR1, RUNX1T1, HMGA2, HIPK1, ZNF711, ZNF710, ZFPM2, LCOR, GLIS3, ZBTB8B, KMT2A, KMT2C, NFYB, NFIX, NFYA, RPA3, HIC2, CHD8, ZNF512B, MCMDC2, CHD2, MLXIP, NFATC2, NFATC3, ZNF700, CHD3, ZBTB7A, CREB1, HMBOX1, RCAN1, DACH1, EXO5, ATXN1, TOX, SP1, IRF2, DENND4A, NFIB			
GOTERM_MF_DIRECT	GO:0000978~RNA polymerase II core promoter proximal region sequence-specific DNA binding	ZNF486, NR6A1, ZNF676, PAX6, PAX5, CTCF, GLI2, ZNF253, MAX, FOS, ZNF148, ZNF737, BHLHE41, MYB, SOX10, PHOX2B, EGR2, ZNF506, GABPA, ESR1, SIX4, MBD2, ZNF138, MTF1, ZNF431, CUX2, MED1, ONECUT2, ELK1, MYBL1, TCF7L2, ZNF331, CHD7, HOXA5, BCL11B, BCL11A, NFAT5, GATAD2B, NFATC2, RUNX2, NFATC3, RFX8, ZBTB7A, AEBP2, CREB1, RFX7, SMAD4, TEAD1, ARID3B, ZNF626, FOXP1, SP1, ETS2, MZF1, RFX3, ZNF117, NEUROD6, TCF12, ZNF257, NFIB	1.975849557	5.25E-07	2.16E-04
GOTERM_MF_DIRECT	GO:0003676~nucleic acid binding	RNASEH1, SYNCRIP, SART3, ZNF253, GTF2IRD2B, ZNF778, ZFP91, ZNF181, ZFP90, ZNF773, DHX36, PTBP2, ZNF445, ZNF180, HFM1, ZNF449, PAN2, RREB1, ZNF506, ZNF649, ZNF791, ZNF189, ZNF793, PRDM7, CELF3, ZNF431, ZNF436, ZNF841, ZNF232, ZNF619, ZNF514, ZNF615, ZNF331, WT1, ZNF846, HNRNPA3, ZNF229, PEG10, HNRNPD, ZNF74, VEZF1, ZNF420, AEBP2, IKZF4, KLF7, IKZF3, KLF9, SREK1, RNPS1, ZNF626, SRSF3, HNRNPH3, ZNF317, MZF1, JAZF1, ZNF117, ZFHX3, ZNF385A, GPATCH8, DDX51, ZNF486, ZNF18, ZKSCAN8, CPEB3, ZNF532, KIAA0430, ZMAT2, ZNF676, ZNF155, ZNF12, ZEB1, GLI2, ZNF346, ZKSCAN5, ZBTB37, RBM4B, ZNF148, ZNF737, ZNF540, KRBOX4, ZNF493, ZNF33A, ZBTB20, EGR2, ZNF544, ZFP30, ZNF354B, ZFX, ZNF333, ZCCHC17, ZFR2, ZNF335, ZNF138, ZNF341, CPSF7, CPSF6, ZNF710, ZFPM2, ZNF550, ZSCAN18, ZNF483, MATR3, THAP8, GLIS3, ZNF554, ZNF275, ZNF559, THAP5, ZNF655, ZNF652, ZNF30, CHD1L, BCL11B, BCL11A, MSI2, POM121C, ZNF700, RBM24, ZNF568, ZFP1, ZNF24, ZNF662, ZNF23, ZNF865, ZSCAN30, SFPQ, ZBTB4, ZNF257, REPIN1	1.531032664	8.15E-07	2.52E-04
GOTERM_MF_DIRECT	GO:0046872~metal ion binding	TMPPE, ZNF253, PRIM1, ZFP91, ZNF778, ZNF773, ZFP90, NUDT17, POLH, RREB1, ZNF506, TRABD2B,	1.333467665	1.51E-06	3.73E-04

GOTERM_MF_DIRECT	GO:0005515~protein binding	ZHX2, ZNF791, BSN, ZNF649, ZNF793, ZNF37A, PITPNM2, MTF1, EXT1, GNAI3, GNAI2, NEK2, ARHGEF28, ZNF232, ASAP1, OAS1, ZNF619, OAS2, RIMS2, ZNF514, ZNF512, ZNF615, ZNF229, ARIH1, CASZ1, ZNF74, VEZF1, LFNG, SEC23IP, ZC3H14, AEBP2, IKZF4, KLF7, IKZF3, KLF9, TGFBR1, SMAD4, ZNF626, TNNI1, ZNF629, NUBPL, ATP2A2, TRPS1, JAZF1, MZF1, ZNF117, ZNF385A, NRP2, TAF1B, ZNF18, ZNF486, ZNF518B, ZNF532, ZNF155, ZNF676, ZNF12, ZEB1, GLI2, ZBTB37, ZNF14, ZNF148, ZNF737, ZNF540, ZNF493, ZFYVE1, ZBTB22, ZBTB20, ZNF33A, POGZ, ACO1, ZFP30, ZNF544, ZNF354B, TP53, PAPD5, ZNF333, ZBTB26, ZNF335, ZNF138, SNRNP48, ZNF341, KSR2, USP21, CHSY1, ZNF550, ZSCAN18, ADAM19, KSR1, ZNF483, REV3L, THAP8, ZNF555, ZNF275, ZNF554, PPP6C, ZNF559, THAP5, ZNF367, ZNF35, KIT, ZNF655, ZNF652, ZNF30, ZFP36L2, CYB561D1, GALNT10, PDE1C, BCL11B, DGKG, BCL11A, GALNT16, GALNT18, PRKAA2, ZNF568, MAT2A, ZNF24, ZFP1, ZNF25, DGKH, ZFP3, DGKI, ZNF662, ZNF23, ZNF865, ZNF362, ZSCAN30, ZBTB4, ZBTB2, NLN, ZNF257, REPIN1, SLC8A3, ZC3HAV1, GTF2E1, ZNF181, ASPA, ZNF182, ZNF445, ZNF180, HMGCL, ZNF449, STK24, ZNF189, RBKS, PDE4C, RPH3A, TESK2, RELN, ZNF431, ZNF436, TSHZ3, TSHZ2, AGFG1, ZNF841, WT1, ZNF331, ZNF846, ITGAX, PRR3, ANKIB1, MBNL3, ZNF420, CACNA2D1, ITGA4, RIMKLA, FOXP1, CACNA2D4, PDZD8, GCM2, ZNF317, EBF3, PDE7A, ZIC5, GPATCH8, METAP1, ZKSCAN8, DICER1, HR, ZKSCAN5, ZNF207, PRMT3, TRIM2, ZFYVE16, PRKCA, ZNF280B, EGR2, DMRT3, ZFX, ADIPOR2, RUNX1T1, IRF2BP2, PRKCE, PGM2L1, ACVR2B, EYA1, PPM1D, NPTXR, PPM1K, ZFYVE26, ZNF711, ZNF710, ZFPM2, GLIS3, MOB1B, ZBTB8B, GALNT6, COL9A1, HIC2, DDHD1, ZNF704, ZNF512B, ZC3H12B, PHF20L1, INPP5B, ZNF700, WDFY3, ZBTB7A, ADARB1, RACGAP1, EXO5, RPAP2, SP1, SP5, BMPR1A RBPMS2, VPS54, VPS53, SYNCRIP, RORB, GRIN3A, MYLIP, WTAP, ITSN1, SART3, AMOTL1, MED20, ZNF778, GRIN2B, MED29, MAP3K9, MAP3K8, DHX36, PIK3CB, LIFR, MECP2, ESPL1, TOX3, DCAF7, ROR1, ARL8A, PMP22, SH3GL2, ORAI2, RAD23B, PPP2R3A, ALCAM, PEA15, ARIH1, DUSP13, INS, GOLPH3L, SGIP1, ZC3H14, SPPL3, SEPT14, NTF3, PAIP2, TAOK1, PAIP1, TMEM120B, ATM, FNIP2, SRSF3, AIDA, SRSF6, FAM214A, PKP4, ARF4, RGS6, TAF1B, LDLR, ATP10B, ZNF346, ZNF148, CEACAM6, USP11, ZNF540, QKI, USP10, IL13RA1, BHLHE41, USP13, SH3PXD2B, SH3PXD2A, ZNF544, TP53, CLIC2,	1.111157665	2.57E-06	5.30E-04

MMP14, WAS, ZNF335, CARD10,
LRPAP1, SS18, ZNF341, EREG,
USP21, SORT1, ZNF550, KSR1,
ARL4C, THOC1, ZNF555, SNAP29,
RABGAP1, ZNF559, ABI2, AZIN1,
ZNF365, NUFIP2, AMPH, FAN1,
ANKS6, SNAP23, USP34, FOXD3,
PHLDA1, SLC8A1, BRD2, UPF1,
ANKHD1, APPL1, ETF1, CNTF,
PLSCR4, SFPQ, GSK3B, IST1,
POLDIP2, USP47, LRP6, USP49,
LRP2, MPZL1, PRC1, LMO4,
EIF4EBP3, FBXO27, ATG7,
FBXO28, TGFBI, RALA, FRS2,
GABPA, FMR1, RPH3A, CCDC6,
DCUN1D1, EP300, HIF1A,
TIMELESS, ARRB1, SMARCA5,
PRCP, TESK2, STMN1, RNF135,
DCUN1D4, ADORA2A, PPIL2,
CCL5, ARNT, MIA3, PEG10,
OSBPL1A, PRKRA, ADRA2A,
FBXW2, PALB2, RHOXF2, FBXO46,
ABCB9, RAB8B, CREBZF, TEAD1,
EHMT2, MPRIP, CDKN1C, ATF5,
MYO10, CDKN1B, CXCL14,
ZNF317, DYRK1A, ABL2, E2F3,
KIF24, RSF1, CDC14A, LRRC8B,
PAX6, PAX5, AMMECR1, PRMT3,
MAX, CASP7, ALS2CR12, SLC1A1,
PPP2R1B, SLA2, PKN2, GAN,
SLC7A11, KIF1C, CLDN1, ZFPM2,
PPP2R2A, UPP2, UBE2R2, MPPE1,
SLC35B4, CD4, BRK1, NFATC2,
EHD1, NFATC3, NOX4, WDFY3,
ADARB1, SNX27, CBL, C1ORF21,
HSP90B1, FAF2, CUL4B, SNX30,
TMEM19, IL6ST, RAB1A, CD47,
PICALM, CD46, GLE1, RECK,
EMX2, TRABD2B, VTI1B, PARP11,
WNK3, DCTN5, SYNJ2BP,
PPARGC1A, VTI1A, RND2, PRDM7,
PNKP, MTF1, RAB18, TRMT12,
PEX26, FBXL5, RAB12, LRCH3,
NEK2, OAS1, OAS2, RIMS2, FUZ,
EPHB2, HNRNPA3, R3HCC1L,
FBXO4, CDC37L1, ST6GAL1,
IKZF3, TP53BP2, NDFIP1, FDXR,
HILPDA, KDR, CCT4, NSMCE4A,
ATP2A2, RNF4, ETS1, ETS2,
CNIH4, GOSR2, TCF12, ZFHX3,
JPH2, CPEB3, PHKB, PCDHA4,
FERMT2, PPARG, ZMAT2, TTLL6,
ZEB1, GLI2, CANX, KCNIP1,
FANCM, GOLGA7, GPX3, GOLGA1,
ZBTB22, PALD1, MBD2, ZBTB26,
MBD1, GRM1, RAD51, FOXR2,
RAB11FIP4, ZDHHC17, REV3L,
THAP8, PPP6C, PARD3, RAB3B,
TFG, FKBP1A, KIT, PHIP, ZFP36L2,
DNALI1, FAM117B, ZDHHC22,
GPM6A, NR1D2, KLC1, BCL11B,
PRKAA2, FAM32A, CSNK1A1,
GDI1, DLGAP2, BCKDHB, RAB32,
YWHAG, COG6, KREMEN1, SVIP,
TMTC2, CAST, SEPT4, CASK,
PMAIP1, FOXO4, GDNF, KRT81,
WNT3, MYOCD, ROBO1, MS4A1,
ITCH, ZNF180, AKIRIN1, HRK,
FAM118A, FOXN2, FOXN3,
TRIM38, IGSF1, NBR1, TRIM32,
AKAP5, PYGO2, CLIP3, DST,
DYNLRB1, GLP1R, GPN1, TWSG1,
TMEM199, PLXNC1, AGFG1,
CCDC14, ADCYAP1R1, SNX4,
ITGB3, ZMYND8, ZNF846, PRR3,
ITGAX, ANKIB1, EIF3J, STK38L,
BMF, EIF3M, RHOBTB3, CFLAR,
ITGA4, TET2, FOXP1, EIF4B,
KCTD9, UBE2E3, TET3, EIF4E,
PHF2, SLAIN2, SPTBN1, RFX3,

CD79A, FAM84B, PLEKHA2, PLEKHA1, PDCD4, LARP1, FOS, RANBP9, SHC1, RANBP2, TUBA1A, C16ORF45, ZW10, CDK13, SHC4, PRKCA, IRAK2, GABRG2, IRAK1, DMRT3, UBE2F, PRKCE, PRKCB, CD84, EYA1, KLHL18, CD80, LARP7, UBE2W, MDM2, MDM4, MATR3, FRK, KRT6B, CYTH1, CAMK2G, RPA3, TNRC6C, NFAT5, POU2F1, SLC39A7, MR1, POM121C, PHF20L1, YES1, TNRC6B, TNRC6A, NEFM, PIK3R1, APC, TXNIP, ZBTB7A, MAP1B, NFASC, CCDC68, MON2, ATXN1, ATXN3, GSPT1, CEP68, IRF2, URI1, SCP2, BMPR1A, XRCC5, LTBP2, C16ORF72, CCDC148, NAA15, INTS2, WDR77, H1F0, TNIK, NCF2, MAGI1, ZHX2, ZNF649, SKP1, CTNNA1, BCL2L11, GRB10, VGLL4, TRIML2, GNAI3, GNAI2, ERBB4, ERBB2, ZNF232, UBA6, HADHA, HEXIM1, SYBU, CDC42EP4, SEC23IP, RPS23, TGFBR1, TNNI1, CCDC150, WDR62, TRPS1, FNBP4, MZF1, H3F3A, ZNF18, NAP1L1, PDIA6, NAP1L2, PLEKHG4B, POMGNT1, GRIPAP1, MAPT, MYB, STX5, STX3, ARHGEF7, ACO1, CMKLR1, CNOT10, TLE3, ERLIN2, ZSWIM7, FLNA, SRPK1, WDR47, CCND2, SRGAP3, USH1C, AICDA, TMSB4Y, EIF5A2, CCDC170, CREM, ZNF655, ZNF652, COL6A5, WIPF2, LIMD2, RAB11A, LIMD1, VMP1, TRIP10, FBXW11, TRIP12, MAT2A, NUB1, VTA1, PPP1R11, ZNF24, ELAVL1, DGKI, TTC17, ZNF23, ZBTB4, ZBTB2, SLC44A3, XPO5, AP1G1, EIF5, STYX, JAG1, CBFB, IQGAP1, LNX1, NRCAM, GTF2E1, ASPA, ANK3, ELOVL3, AAK1, SMARCD1, DDOST, ZNF449, STK24, MTA3, NUDT5, RBL1, ARID1A, RBKS, GRHL2, WEE1, PA2G4, KDM2B, LCLAT1, PTGFRN, SLC40A1, NMNAT1, ZNF436, AZI2, NIPBL, NUMB, TEK, HNRNPD, MBNL3, ZNF420, TRAF6, SMG9, KCNB1, ARID3B, SHANK2, SUV39H2, GLYCTK, HNRNPH3, ARMC8, CDKN2AIP, PRKAR1A, ADAM22, CACNA1B, SEC24B, GPBP1, DEDD, DICER1, PIP5K1B, CTCF, ZKSCAN5, RAB3IP, ZNF207, TRIM5, TRIM2, ZFYVE16, HEY2, DLG4, LBR, DLG2, HEMK1, VASN, CCNL1, ESR1, CDK8, IPO9, HMGA2, PURA, PPM1D, PPM1K, ZFYVE26, RRAS2, IPO5, CPSF7, CPSF6, LCOR, CPSF2, KMT2A, KMT2C, NFYB, NFYA, CDH2, ESYT2, TSPYL5, CHD8, CHD7, ZNF512B, CHD1L, REL, TOR1A, CHD2, PAFAH1B1, FBN2, NEDD4L, INPP5B, CHD3, CHDH, PLP2, PDCD10, NF2, CREB1, NF1, DACH1, TAB2, FAM9C, SMC3, IFIT3, NEB, APAF1, SYT1, ATP1B1, VAPA, VAPB, SNCA, ERLEC1, ZNRF3, AGPS, GAB1, INSIG1, PAN2, POLH, VASH1, PDGFRA, EFNA4, TAF9B, ASAP1, IGF2BP1, IGF2BP2, TRAM1, OSBPL3, TRPC3, SREK1, SMAD4, RAD54L, CHRNB2, KIAA0368, CCNT1, FCRL2, NFKB1, COPS8, CAMKK1,

		CKB, RBM4B, DNAJC15, BAG1, NR2F2, IP6K1, SH2D1B, ZFYVE1, SLC25A4, POGZ, POLR1D, FLOT2, POLR1A, FAM76B, GTF2B, ZCCHC17, PDIK1L, SIAH2, TBX18, MED1, LANCL1, STAT6, SUMO1, SET, STK40, GATAD2B, AGO3, AGO4, THBS1, DTNA, NOS1, NLK, FZD1, FZD3, MAPK10, ITPR3, FZD4, CAPRIN2, IYD, MAPK13, MRPL27, PSMD11, CDON, CALM2, TP53INP2, FAM126A, TP53INP1, STIL, ZC3HAV1, CHMP4C, FGF14, TBC1D19, KLHL3, CBX6, ANKRD52, TBC1D14, SPRED2, SLC4A4, NEGR1, SOCS3, BASP1, TBC1D22B, SLC38A1, ZFAND5, TSHZ3, TSHZ2, SYVN1, PKHD1, SOX5, ELK1, ENSA, WT1, PAQR5, PPP1R16B, HOXA5, STRBP, ENTPD1, AXIN2, RUNX2, MAFG, SESTD1, CPNE4, LPP, RNPS1, SUGT1, REEP5, GCM2, PSMC6, BBC3, RHOT1, AHCYL1, TBC1D20, GPATCH8, BMI1, PRX, NAMPT, C9ORF72, TLN2, BORA, SLA, KCNQ5, CTTN, KCNQ4, CNTNAP2, SV2B, C11ORF87, AKT3, SOX10, RSU1, EGR2, KHDRBS2, MIEF1, RUNX1T1, LDB3, MPP6, DYNLT1, MPP7, EIF4G1, EIF4G2, ACVR2B, CTH, CRKL, HIPK1, BTG2, ZNF711, KPNA6, PPP1R15B, KPNA2, KPNA1, CALCR, MOB1B, NANOS1, PPP3R1, NFIX, GSG1L, TCF7L2, TPM3, PSMF1, MSI2, DNM1L, PTPRA, HMBOX1, SUN2, RACGAP1, GORAB, ATXN7L3, SP1, CASP14, MYPN, KIAA0319, ATP8A1			
GOTERM_MF_DIRECT	GO:0003682~chromatin binding	EZH1, CCNT1, PPARG, PAX6, NFKB1, ZEB1, NAP1L2, GLI2, FOS, FANCM, SMARCD1, SOX10, EGR2, GABPA, FMR1, MTA3, TP53, MBD6, ESR1, POLR1A, MECP2, MBD5, MBD2, MBD1, TOX3, PRKCB, RAD51, EP300, PYGO2, ZNF431, NSD1, MED1, TSHZ3, TSHZ2, USP3, KMT2A, ELK1, ZMYND8, ARX, CHD8, CHD7, NIPBL, REL, HNRNPD, AUTS2, PRKAA2, NFATC2, RUNX2, BRD2, UPF1, NUCKS1, SMAD4, FOXP1, SMC3, ATF5, SFPQ, TRPS1, URI1, ATAD2B	1.764031073	2.41E-05	0.00394127
GOTERM_MF_DIRECT	GO:0043565~sequence-specific DNA binding	ZKSCAN8, ZNF532, EVX2, NR6A1, PPARG, PAX6, CTCF, RORB, FOXO4, GLI2, MAX, FOS, GTF2E1, ZNF148, HEY2, MKX, RARB, NR2F2, GATAD1, ZNF449, GABPA, MTA3, EMX2, TP53, ESR1, FOXN2, RHOXF2B, SIX4, PPARGC1A, GRHL2, FOXN3, FOXR2, HOXC10, HIF1A, ZNF711, CUX2, CAMTA1, IRX5, CREM, TFCP2L1, ELK1, ZNF232, NFYB, ZNF35, TCF7L2, WT1, ARNT, DRGX, NR1D2, HOXA5, BCL11B, POU2F1, GATAD2B, NKX2-4, RHOXF2, CREBL2, MAFG, IKZF4, IKZF3, CREBZF, CREB1, ZNF24, SMAD4, FOXP1, ATF5, GCM2, SP1, ETS1, ZSCAN30, ETS2, TRPS1, ZBTB4, PBX3	1.647494893	2.55E-05	0.00394127
GOTERM_MF_DIRECT	GO:0008270~zinc ion binding	LMO4, SNCA, RORB, MYLIP, TRIM51, ZNF253, ZNRF3, ZNRF2, LNX1, RNF141, GRIN2B, PGLYRP4, PAPPA, RARB, RC3H1, MTA3, UBR1, RPH3A, ADAMTS6, TRIM38, KDM2B, EP300, MTF2,	1.400053079	3.15E-05	0.004327

		NBR1, TRIM32, PYGO2, CPD, RNF135, ZFAND5, SYVN1, CXXC4, OAS1, OAS2, MYT1, ZMYND8, WT1, ZNF331, ARIH1, TRIM66, PEG10, TCF20, RNF165, SLC30A5, ANKIB1, TRAF6, PGGT1B, KLF7, ZMYM2, LPP, TET2, EHMT2, SUV39H2, MSL2, TET3, PHF3, PHF2, RNF150, RNF4, TRPS1, PRICKLE2, ZNF117, ZFHX3, ZNF385A, BMI1, SEC24B, RSF1, NR6A1, ZMAT2, PPARG, MMP26, CTCF, ZEB1, RLIM, GLI2, ZNF346, ZKSCAN5, ZNF207, RBM4B, TRIM5, TRIM2, MBTD1, RANBP2, NR2F2, GATAD1, ZFYVE1, USP13, PRKCA, ZCCHC3, ZDHHC7, TP53, LDB3, POLR1A, ZSWIM6, ESR1, ZSWIM7, MMP16, MMP14, ZFR2, MBD1, GTF2B, ZCCHC17, PRKCB, ZDHHC17, ZDHHC13, MDM2, AICDA, ZFPM2, ZADH2, SIAH2, MDM4, NSD1, MATR3, CPM, ADAMTS14, KMT2A, USP3, KMT2C, NANOS1, LANCL1, ZDHHC22, NR1D2, GATAD2B, LIMD2, LIMD1, CD4, DTNA, CHD3, DTX4, RNF144A, UPF1, MEX3A, CBL, ZNF24, PDZRN4, MARCH6, TAB2, ATXN7L3, RNF44, USP49, ZNF257			
GOTERM_MF_DIRECT	GO:0061630~ubiquitin protein ligase activity	SYVN1, PPIL2, MYLIP, RLIM, MED20, ZNRF3, UBE2R2, ZNRF2, ARIH1, C18ORF25, RNF165, ANKIB1, FBXO4, ITCH, TRAF6, FBXW11, RC3H1, RNF144A, CBL, UBE2J1, UBE2F, SKP1, UBR1, MSL2, RNF44, RNF4, TRIM32, MDM2, UBE2W, FBXL22, MDM4, CUL4B, SIAH2, MED1	2.12553513	4.87E-05	0.00602443
GOTERM_MF_DIRECT	GO:0001077~transcriptional activator activity, RNA polymerase II core promoter proximal region sequence-specific binding	CAMTA1, ONECUT3, NR6A1, ONECUT2, PAX6, PAX5, ELK1, MYBL1, GLI2, WT1, FOS, MYOCD, HOXA5, BCL11B, NFAT5, NFATC2, MYB, RUNX2, NFATC3, PHOX2B, EGR2, NUCKS1, CREB1, SOX11, GABPA, ESR1, SMAD4, ARID3B, TEAD1, SIX4, HMGA2, GCM2, HIF1A, MTF1, EBF3, MZF1, NEUROD6, TCF12, NFIB	1.931895277	1.07E-04	0.01200685
GOTERM_MF_DIRECT	GO:0044212~transcription regulatory region DNA binding	XRCC5, ERBB4, KMT2A, ZNF532, CCNT1, PPARG, TAF9B, SNCA, NFKB1, NFYB, CTCF, NFYA, TCF7L2, WT1, FOS, TCF20, BCL11B, DHX36, IKZF4, IKZF3, EGR2, CREB1, GABPA, TP53, SMAD4, ZNF649, BASP1, ZNF335, ATF5, SP1, ARRB1, SFPQ, MZF1, RFX3, ZFHX3, TCF12	1.975849557	1.31E-04	0.01345816
GOTERM_MF_DIRECT	GO:0001046~core promoter sequence-specific DNA binding	KMT2A, USP3, CREM, PPARG, ESR1, TP53, NFYA, MYOCD, SP1, NR1D2, CHD2, ZNF431, ZFHX3	3.534320041	1.82E-04	0.01735133
GOTERM_MF_DIRECT	GO:0005547~phosphatidylinositol-3,4,5-trisphosphate binding	MYO10, PARD3, PIRT, JPH2, MYO1B, ZFYVE16, FERMT2, ASAP1, KIF16B, RACGAP1, ZFYVE1, IQGAP1	3.691718909	2.36E-04	0.02088292
GOTERM_MF_DIRECT	GO:0035035~histone acetyltransferase binding	ZBTB7A, HIF1A, SP1, MTF1, MYOCD, ETS1, CREB1, TP53, PAX6, KANSL1L	4.17515829	3.67E-04	0.03026936
GOTERM_MF_DIRECT	GO:0008134~transcription factor binding	LMO4, CCNT1, TAF9B, PPARG, PAX6, NFKB1, RORB, ZEB1, FOXO4, TCF7L2, ARNT, FOS, SUMO1, MYOCD, ATG7, HEY2, GPX3, HNRNPD, NFATC2, PIK3R1, SOX10, NUCKS1, NLK, RBL1, TP53, MECP2, ESR1, HMGA2, PPARGC1A, GTF2B, FLNA, PURA, HIF1A, EP300, RNF4, SP1, ARRB1, ETS1, ZFPM2, TCF12, MED1	1.68770483	0.00117462	0.09081248

GOTERM_MF_DIRECT	GO:0019838~growth factor binding	NRP2, ACVR2B, LTBP2, IL6ST, TGFBR1, ERBB2, TEK, LIFR, KDR	3.896814404	0.00141686	0.10309749
GOTERM_MF_DIRECT	GO:0000979~RNA polymerase II core promoter sequence-specific DNA binding	RREB1, SOX11, PAX6, ZNF335, STAT6, FOS, EP300, KDM2B, MTF1, GBX2, H3F3A, RUNX2, NSD1	2.666241435	0.00275374	0.18924281
GOTERM_MF_DIRECT	GO:0004842~ubiquitin-protein transferase activity	HECW2, PPIL2, PAX6, MYLIP, KLHL4, RLIM, ASB15, KLHL3, ASB18, ZNRF3, ZNRF2, LNX1, UBE2R2, TRIM5, ARIH1, ZFP91, TRIM2, RNF141, HECTD2, KBTBD8, ANKIB1, FBXW2, FBXO4, ITCH, NEDD4L, TRAF6, FBXW11, TRIP12, DTX4, RNF144A, CBL, PDZRN4, GAN, SKP1, MARCH6, UBE2E3, KLHL18, RNF4, TRIM32, FBXL5, UBE2W, MDM2, SIAH2, RNF135	1.56346353	0.00338095	0.22011754
GOTERM_MF_DIRECT	GO:0031624~ubiquitin conjugating enzyme binding	RNF144A, ARIH1, ZMYM2, DCUN1D1, ANKIB1, SIAH2, TRAF6, MARCH6, DCUN1D4	3.393999643	0.00370382	0.22908109
GOTERM_MF_DIRECT	GO:0005545~1-phosphatidylinositol binding	WDFY3, SYT1, PICALM, SESTD1, ZFYVE16, ZFYVE1, PLEKHA2	4.091655125	0.00524673	0.30905733
GOTERM_MF_DIRECT	GO:0019903~protein phosphatase binding	PARD3, NEK2, ERBB2, PPARG, TP53, CDH2, IQGAP1, STAT6, HSP90B1, CDKN1B, DLG4, ANAPC7, PIK3R1	2.412313679	0.00646332	0.36341466
GOTERM_MF_DIRECT	GO:0003730~mRNA 3'-UTR binding	ZFP36L2, RBM24, CPEB3, FMR1, IGF2BP1, ELAVL1, IGF2BP2, RNPS1, ZNF385A, RC3H1, LARP1	2.624385211	0.00759126	0.3994219
GOTERM_MF_DIRECT	GO:0031625~ubiquitin protein ligase binding	XRCC5, PAX6, ABI2, ASB15, ASB18, UBE2R2, CKB, ARIH1, SUMO1, KBTBD4, RALA, AXIN2, TRAF6, USP13, TXNIP, DNM1L, EGR2, NLK, TP53, UBE2J1, UBE2F, ERLIN2, PPARGC1A, FZD4, BTBD9, DBT, PA2G4, ATXN3, HIF1A, DIO2, ARRB1, GSK3B, PRKAR1A, MDM2, UBE2W, FAF2, AICDA, CUL4B	1.547863561	0.0077495	0.3994219
GOTERM_MF_DIRECT	GO:0005088~Ras guanyl-nucleotide exchange factor activity	ERBB4, ERBB2, CAMK2G, PTPRA, RASGEF1A, KIT, GDNF, GRIN2B, EREG, TEK, PDGFRA, DLG4, SPTBN1, SHC1, NRG1, FRS2, CALM2, GFRA2, IL2	1.931464531	0.0087055	0.42012129
GOTERM_MF_DIRECT	GO:0000166~nucleotide binding	RBPMS2, CPEB3, SRSF12, KIAA0430, IGF2BP1, SYNCRIP, IGF2BP2, SART3, HNRNPA3, RBM4B, R3HCC1L, CHD1L, TNRC6C, HNRNPD, MSI2, PTBP3, PTBP2, TNRC6B, TNRC6A, CYR61, NOX4, RBM24, MOCS2, SREK1, ELAVL1, RNPS1, RCAN1, PPARGC1A, HNRNPA0, RAD51, SRSF3, EIF4B, HNRNPH3, TRNAU1AP, SRSF6, LARP7, SFPQ, CPSF7, RBMXL2, CELF3, CPSF6, APAF1, MATR3, REV3L	1.478102015	0.00909341	0.42012129
GOTERM_MF_DIRECT	GO:0044822~poly(A) RNA binding	CAST, XRCC5, ZC3HAV1, XPO5, EIF5, NAA15, SYNCRIP, SART3, PTBP3, DHX36, PTBP2, MRPL32, FNDC3A, RC3H1, MTO1, FNDC3B, H1F0, GTPBP1, FMR1, MECP2, CTNNA1, PA2G4, UBFD1, GIGYF2, SRSF12, IGF2BP1, IGF2BP2, HNRNPA3, PEG10, TCF20, PRR3, PRKRA, NGRN, HNRNPD, MBNL3, STRBP, CDC42EP4, NOVA2, SEC23IP, NOVA1, RPS23, ZC3H14, SREK1, MCAT, RNPS1, HNRNPA0, SRSF3, EIF4B, HNRNPH3, CCT4, EIF4E, SRSF6, SPTBN1, ZNF385A, GPATCH8, DDX51, IBA57, CPEB3, NAP1L1, TRMT10A, ZNF346, CANX, LARP1, ZNF207, BZW1, RBM4B, FAM133B, TRMT6, QKI, USP10, LBR, CDK13, ZCCHC3, PKN2, ANKHD1-EIF4EBP3, PAPD5, ZCCHC17, SRPK1, FLNA, PURA, KIF1C, EIF4G1, EIF4G2,	1.242562609	0.00916999	0.42012129

		TRNAU1AP, CRKL, LARP7, IPO5, CPSF7, CPSF6, KPNA2, MATR3, MYO5A, FAM98B, KMT2C, WBSCR22, PDAP1, NUFIP2, ZFP36L2, SUMO1, CHD2, MSI2, AGO3, TNRC6B, FAM32A, TNRC6A, CHD3, UPF1, ADARB1, NBPF10, NUCKS1, MEX3A, ELAVL1, ANKHD1, ETF1, YWHAG, GSPT1, MRPL27, IFIT5, SFPQ, REPIN1			
GOTERM_MF_DIRECT	GO:0017124~SH3 domain binding	KHDRBS2, TP53BP2, CBL, LANCL1, GRIK5, ABI2, AFAP1L2, WAS, SHANK2, SH3BP5L, CCDC6, PLSCR4, MAPT, MYPN, QKI, DNAJC6, ADAM19, LRP2, SGIP1	1.866541353	0.01226218	0.53219059
GOTERM_MF_DIRECT	GO:0001078~transcriptional repressor activity, RNA polymerase II core promoter proximal region sequence-specific binding	ZBTB7A, AEBP2, TSHZ3, TSHZ2, ZBTB20, CTCF, DACH1, HMGA2, BTG2, ZNF148, ETS2, ZFP90, BCL11A, MZF1, ZFPM2, CUX2, BHLHE41, NFATC2	1.895747548	0.01303976	0.53219059
GOTERM_MF_DIRECT	GO:0003723~RNA binding	XPO5, CPEB3, ZMAT2, KIAA0430, SYNCRIP, RNASEH1, RLIM, PDCD4, QKI, PTBP3, RANBP2, PTBP2, DLG2, ACO1, FMR1, PPARGC1A, PURA, EIF4G1, EIF4G2, EYA1, LARP7, TRIM32, CELF3, CPSF6, CPSF2, THOC1, PUS3, AGFG1, SRSF12, ARHGEF28, NANOS1, IGF2BP1, NUFIP2, WT1, RPP14, HNRNPA3, TNRC6C, TDRKH, HNRNPD, ZNF74, STRBP, NOVA2, NOVA1, ADARB1, UPF1, PAIP1, ELAVL1, RNPS1, ANKHD1, ETF1, HNRNPA0, CAPRIN2, ATXN1, IFIT3, SRSF3, EIF4B, HSP90B1, HNRNPH3, SRSF6, IFIT5, CDKN2AIP, RBMXL2, AHCYL1	1.346431302	0.01327086	0.53219059
GOTERM_MF_DIRECT	GO:0004674~protein serine/threonine kinase activity	CDK19, NEK2, CAMK2G, CCNT1, CASK, CAMKK1, STK40, SBK1, AAK1, MAP3K9, MAP3K8, PRKAA2, STK38L, AKT3, CDK13, CSNK1A1, PRKCA, IRAK2, IRAK1, ALPK1, TNIK, TAOK1, STK24, NLK, TGFBR1, CDK8, PKN2, WNK3, PRKCE, MARK1, ATM, SRPK1, PRKCB, PDIK1L, MAST4, ACVR2B, KSR2, HIPK1, MAPK13, HIPK3, GSK3B, DYRK1A, TESK2, NRK, BMPR1A, CAMK1D	1.430213797	0.01333703	0.53219059
GOTERM_MF_DIRECT	GO:0016874~ligase activity	TRIML2, SYVN1, PPIL2, TTLL6, MYLIP, RLIM, TTLL1, ZNRF3, LNX1, ZNRF2, TRIM5, ARIH1, ZFP91, TRIM2, C18ORF25, RNF165, HECTD2, RANBP2, NEDD4L, TRAF6, TRIP12, DTX4, RNF144A, EGR2, CBL, UBE2F, UBR1, MARCH6, TRIM38, MSL2, RNF4, TRIM32, MDM2, SIAH2, RNF135	1.515427824	0.01447539	0.55542742
GOTERM_MF_DIRECT	GO:0071837~HMG box domain binding	EGR2, SP1, PAX6, OLIG2, ALX4, TCF12	3.896814404	0.01511699	0.55542742
GOTERM_MF_DIRECT	GO:0044325~ion channel binding	SLC8A1, NOS1, PIRT, SCN3B, KCNB1, FMR1, FKBP1A, RIMS2, FLNA, RIMS3, SUMO1, ARRB1, ANK3, NPTN, USP10, NEDD4L, YES1, CALM2	1.862194494	0.01541309	0.55542742
GOTERM_MF_DIRECT	GO:0005484~SNAP receptor activity	SNAP29, STX5, STX3, VTI1B, GOSR2, GOSR1, SNAP23, STX1B, VTI1A	2.697794588	0.01571541	0.55542742
GOTERM_MF_DIRECT	GO:0032266~phosphatidylinositol-3-phosphate binding	SH3PXD2B, PARD3, JPH2, SESTD1, SNX27, ZFYVE26, KIF16B, SNX24	2.922610803	0.01654192	0.56839877
GOTERM_MF_DIRECT	GO:0017137~Rab GTPase binding	MYO5A, GDI1, RAB3GAP2, C9ORF72, DNM1L, RABGAP1, AP1G1, KIF16B, RIMS2, RPH3A, TBC1D22B, USP6NL, DENND1B, RAB11FIP4, CHML, TBC1D14,	1.744842271	0.01958697	0.65484018

		TBC1D13, EHD1, TBC1D20, RHOBTB3			
GOTERM_MF_DIRECT	GO:0004672~protein kinase activity	CDK19, NEK2, ERBB2, CASK, CCL5, MAP3K9, AAK1, MAP3K8, TEK, PRKAA2, STK38L, AKT3, CDK13, CSNK1A1, PRKCA, IRAK1, TNIK, TAOK1, STK24, NLK, TGFBR1, PKN2, CDK8, WNK3, PKDCC, MAPK10, PRKCE, WEE1, SRPK1, PRKCB, KSR2, HIPK1, CAMK4, MAPK13, HIPK3, GSK3B, DYRK1A, PDGFRA, ROR1, TESK2, NRK, KSR1, ABL2	1.400248073	0.02308262	0.75140017
GOTERM_MF_DIRECT	GO:1990841~promoter-specific chromatin binding	BMI1, CHD7, RBL1, EHMT2, FOXO4, PPARGC1A	3.507132964	0.02372239	0.75242559
GOTERM_MF_DIRECT	GO:0035197~siRNA binding	FMR1, DICER1, MECP2, MBD2	5.845221607	0.02516116	0.77810888
GOTERM_MF_DIRECT	GO:0001228~transcriptional activator activity, RNA polymerase II transcription regulatory region sequence-specific binding	MAFG, IKZF3, GABPA, TP53, SMAD4, ARID3B, CTCF, GRHL2, ATF5, EP300, HIF1A, MYOCD, TRPS1, IRF2, ALX4	1.845859455	0.03079307	0.92904934
GOTERM_MF_DIRECT	GO:0003725~double-stranded RNA binding	ADARB1, PRKRA, DICER1, ELAVL1, OAS1, AGO3, DHX36, AGO4, OAS2, STRBP, ZNF346	2.108112711	0.03300488	0.96448927
GOTERM_MF_DIRECT	GO:0005159~insulin-like growth factor receptor binding	YWHAG, ARRB1, INS, SHC1, PIK3R1	3.896814404	0.03380189	0.96448927
GOTERM_MF_DIRECT	GO:0048027~mRNA 5'-UTR binding	FMR1, IGF2BP1, IGF2BP2, LARP1	5.195752539	0.0353828	0.96448927
GOTERM_MF_DIRECT	GO:0046934~phosphatidylinositol-4,5-bisphosphate 3-kinase activity	EREG, ERBB4, CD80, PIK3CB, ERBB2, GAB1, PDGFRA, KIT, NRG1, FRS2, PIK3R1	2.074110893	0.0364531	0.96448927
GOTERM_MF_DIRECT	GO:0016301~kinase activity	MOB1B, PHKB, FGGY, ERBB2, CASK, SGMS1, TK2, FLT3LG, CKB, MAP3K9, PRKRA, PRKAA2, PIK3R1, CSNK1A1, PRKCA, IRAK1, ALPK1, TAOK1, PIK3CB, CHKB-CPT1B, PKN2, TAB2, MARK1, WEE1, CDKN1C, CDKN1B, GSK3B, PRKAR1A, NRK, CALM2	1.455241894	0.03821451	0.96448927
GOTERM_MF_DIRECT	GO:0017112~Rab guanyl-nucleotide exchange factor activity	RAB3GAP2, SBF1, DENND4A, DENND5B, ST5, RAB3IP, DENND1B	2.727770083	0.03867887	0.96448927
GOTERM_MF_DIRECT	GO:0036033~mediator complex binding	NIPBL, SMC3, MED1	8.76783241	0.03898617	0.96448927
GOTERM_MF_DIRECT	GO:0043125~ErbB-3 class receptor binding	ERBB2, NRG1, PIK3R1	8.76783241	0.03898617	0.96448927
GOTERM_MF_DIRECT	GO:0070579~methylcytosine dioxygenase activity	TET3, ALKBH1, TET2	8.76783241	0.03898617	0.96448927
GOTERM_MF_DIRECT	GO:0004714~transmembrane receptor protein tyrosine kinase activity	ERBB4, ERBB2, TEK, PDGFRA, ROR1, NPTN, KIT, KDR	2.46114594	0.03976472	0.96448927
GOTERM_MF_DIRECT	GO:0005154~epidermal growth factor receptor binding	EREG, ERBB4, ARF4, MS4A1, SHC1, SNX4, YES1	2.6397775	0.04462001	1
GOTERM_MF_DIRECT	GO:0005158~insulin receptor binding	PHIP, GRB10, INS, SHC1, SNX4, FRS2, PIK3R1	2.6397775	0.04462001	1
GOTERM_MF_DIRECT	GO:0008013~beta-catenin binding	ESR1, SKP1, CDH2, CTNNA1, FOXO4, TCF7L2, CHD8, EP300, GSK3B, NUMB, BCL9L, AXIN2, APC	1.853362948	0.04543122	1
GOTERM_MF_DIRECT	GO:0003707~steroid hormone receptor activity	NR1D2, NR6A1, PPARG, ESR1, ABHD2, RORB, RARB, OR51E2, NR2F2, PAQR5	2.087579145	0.04672061	1
GOTERM_MF_DIRECT	GO:0004716~receptor signaling protein tyrosine kinase activity	ERBB4, ERBB2, KIT, KDR	4.676177285	0.04740325	1
GOTERM_MF_DIRECT	GO:0000980~RNA polymerase II distal enhancer sequence-specific DNA binding	ARX, SOX10, REL, SFPQ, CREB1, ARID3B, GATAD2B, H3F3A, NFKB1, MBD2, PBX3	1.978382698	0.04827369	1

**pathways and processes involved in severe malaria-associated processes*

***p value obtained using Benjamini-Hochberg correction*

Table S5: Results of the receiver operating characteristic (ROC) curve analysis to detect the fatal cerebral malaria (CM) patients.

	**Markers***	**AUC (95%CI) [%]**	**p value**	**Cut-off**	**Sensitivity [%]**	**Specificity [%]**
fatal CM *vs*. non-fatal CM	hsa-miR-150-5p	88.6 (77.6-99.7)	0.005	17.28	100	78.4
	hsa-miR-3158-3p	77.8 (57.2-98.5)	0.045	1.08	80	75.7
fatal CM *vs*. SNCM	hsa-miR-150-5p	91.3 (80.5-100)	0.004	16.55	100	82.6
	hsa-miR-3158-3p	79.1 (60.8-97.5)	0.044	1.05	80	73.9
fatal CM *vs*. UM	hsa-miR-150-5p	97.1 (90.3-100)	0.002	16.48	100	92.9
	hsa-miR-3158-3p	95.7 (87.2-100)	0.003	0.39	100	85.7
fatal CM *vs*. HC	hsa-miR-150-5p	100 (100-100)	0.0004	14.66	100	96.9
	hsa-miR-3158-3p	78.1 (48.9-100)	0.046	1.14	85	75

SNCM: severe non-cerebral malaria; UM: uncomplicated malaria; HC: European malaria-naive adults; AUC: Area under the ROC curve; CI: confidence interval.

**combination of hsa-miR-150-5p and hsa-miR-3158-3p using logistic regression did not improve AUC, sensitivity and specificity values significantly*

Table S6: Adjusted p values were obtained using the Benjamini-Hochberg correction method.

Comparisons	miRNAs	P value	Benjamini-Hochberg P-value
SM vs. UM	miR-222-3p	0.0004	0.0035
	miR-150-5p	0.0007	0.0035
	miR-146a-5p	0.004	0.014
	miR-3158-3p	0.014	0.035
	mir-16-5p	0.063	0.126
	miR-223-3p	0.175	0.292
	miR-155-5p	0.215	0.307
	miR-27a-3p	0.494	0.617
	miR-210-3p	0.816	0.907
	miR-451a	0.945	0.945
CM vs. UM	miR-222-3p	0.000617	0.002
	miR-3158-3p	0.001	0.002
	miR-150-5p	0.002	0.003
	miR-146a-5p	0.004	0.004
CM vs. HC	miR-222-3p	0.000001	0.000004
	miR-146a-5p	0.000006	0.000012
	miR-150-5p	0.006	0.008
	miR-3158-3p	0.175	0.175
SNCM vs. UM	miR-150-5p	0.002	0.008
	miR-222-3p	0.004	0.008
	miR-146a-5p	0.031	0.041
	miR-3158-3p	0.049	0.049
SNCM vs. HC	miR-222-3p	0.000022	0.000088
	miR-146a-5p	0.000068	0.000136
	miR-150-5p	0.039	0.046
	miR-3158-3p	0.046	0.046
UM vs. HC	miR-3158-3p	0.000013	0.000052
	miR-150-5p	0.375	0.648
	miR-146a-5p	0.486	0.648
	miR-222-3p	0.804	0.804
fatal CM vs. non-fatal CM	miR-150-5p	0.003	0.012
	miR-3158-3p	0.045	0.09
	miR-222-3p	0.123	0.164
	miR-146a-5p	0.426	0.426
fatal CM^+ vs. non-fatal CM^+	miR-150-5p	0.006	0.024
	miR-3158-3p	0.03	0.06
	miR-222-3p	0.109	0.145
	miR-146a-5p	0.614	0.614
fatal CM^+ vs. non-fatal CM*	miR-150-5p	0.008	0.032
	miR-146a-5p	0.206	0.31
	miR-3158-3p	0.254	0.31
	miR-222-3p	0.31	0.31
Day0 vs. Day30	miR-3158-3p	0.031	0.124
	miR-146a-5p	0.156	0.208
	miR-222-3p	0.156	0.208
	miR-150-5p	0.312	0.312

SM: severe malaria; UM: uncomplicated malaria; CM: cerebral malaria; SNCM: severe non-cerebral malaria; HC: European malaria-naive adults; CM^+: CM patient with another concomitant organ involvement; CM: patients with CM alone*

REFERENCES

1. World Health Organization. Guidelines for the treatment of malaria. Third edition, April 2015. https://www.who.int/malaria/publications/atoz/9789241549127/en/. Accessed 01/09/2020, 2020.
2. Severe malaria. *Trop Med Int Health.* 2014;19 Suppl 1:7-131.
3. Mohanty S, Benjamin LA, Majhi M, et al. Magnetic Resonance Imaging of Cerebral Malaria Patients Reveals Distinct Pathogenetic Processes in Different Parts of the Brain. *mSphere.* 2017;2(3).
4. Barker KR, Lu Z, Kim H, et al. miR-155 Modifies Inflammation, Endothelial Activation and Blood-Brain Barrier Dysfunction in Cerebral Malaria. *Mol Med.* 2017;23:24-33.
5. Baro B, Deroost K, Raiol T, et al. Plasmodium vivax gametocytes in the bone marrow of an acute malaria patient and changes in the erythroid miRNA profile. *PLoS Negl Trop Dis.* 2017;11(4):e0005365.
6. Chamnanchanunt S, Fucharoen S, Umemura T. Circulating microRNAs in malaria infection: bench to bedside. *Malar J.* 2017;16(1):334.
7. Cohen A, Zinger A, Tiberti N, Grau GER, Combes V. Differential plasma microvesicle and brain profiles of microRNA in experimental cerebral malaria. *Malar J.* 2018;17(1):192.
8. El-Assaad F, Hempel C, Combes V, et al. Differential microRNA expression in experimental cerebral and noncerebral malaria. *Infect Immun.* 2011;79(6):2379-2384.
9. Gupta H, Rubio M, Sitoe A, et al. Plasma MicroRNA Profiling of Plasmodium falciparum Biomass and Association with Severity of Malaria Disease. *Emerg Infect Dis.* 2021;27(2).
10. Martin-Alonso A, Cohen A, Quispe-Ricalde MA, et al. Differentially expressed microRNAs in experimental cerebral malaria and their involvement in endocytosis, adherens junctions, FoxO and TGF-beta signalling pathways. *Sci Rep.* 2018;8(1):11277.
11. Rangel G, Teerawattanapong N, Chamnanchanunt S, Umemura T, Pinyachat A, Wanram S. Candidate microRNAs as Biomarkers in Malaria Infection: A Systematic Review. *Curr Mol Med.* 2019;20(1):36-43.
12. LaMonte G, Philip N, Reardon J, et al. Translocation of sickle cell erythrocyte microRNAs into Plasmodium falciparum inhibits parasite translation and contributes to malaria resistance. *Cell Host Microbe.* 2012;12(2):187-199.
13. Wang Z, Xi J, Hao X, et al. Red blood cells release microparticles containing human argonaute 2 and miRNAs to target genes of Plasmodium falciparum. *Emerg Microbes Infect.* 2017;6(8):e75.
14. Marabita F, de Candia P, Torri A, Tegner J, Abrignani S, Rossi RL. Normalization of circulating microRNA expression data obtained by quantitative real-time RT-PCR. *Brief Bioinform.* 2016;17(2):204-212.
15. Metsalu T, Vilo J. ClustVis: a web tool for visualizing clustering of multivariate data using Principal Component Analysis and heatmap. *Nucleic Acids Res.* 2015;43(W1):W566-570.
16. Ioannidis LJ, Nie CQ, Hansen DS. The role of chemokines in severe malaria: more than meets the eye. *Parasitology.* 2014;141(5):602-613.
17. Rowe JA, Claessens A, Corrigan RA, Arman M. Adhesion of Plasmodium falciparum-infected erythrocytes to human cells: molecular mechanisms and therapeutic implications. *Expert Rev Mol Med.* 2009;11:e16.
18. Banerjee R, Khandelwal S, Kozakai Y, Sahu B, Kumar S. CD47 regulates the phagocytic clearance and replication of the Plasmodium yoelii malaria parasite. *Proc Natl Acad Sci U S A.* 2015;112(10):3062-3067.

19. Green YS, Sargis T, Reichert EC, et al. Hypoxia-Associated Factor (HAF) Mediates Neurofibromin Ubiquitination and Degradation Leading to Ras-ERK Pathway Activation in Hypoxia. *Mol Cancer Res.* 2019;17(5):1220-1232.
20. Cardone J, Le Friec G, Vantourout P, et al. Complement regulator CD46 temporally regulates cytokine production by conventional and unconventional T cells. *Nat Immunol.* 2010;11(9):862-871.
21. Morgan-Rowe L, Nikitorowicz J, Shiwen X, et al. Thrombospondin 1 in hypoxia-conditioned media blocks the growth of human microvascular endothelial cells and is increased in systemic sclerosis tissues. *Fibrogenesis Tissue Repair.* 2011;4:13.
22. Mingyuan X, Qianqian P, Shengquan X, et al. Hypoxia-inducible factor-1alpha activates transforming growth factor-beta1/Smad signaling and increases collagen deposition in dermal fibroblasts. *Oncotarget.* 2018;9(3):3188-3197.
23. Zhang H, Akman HO, Smith EL, Zhao J, Murphy-Ullrich JE, Batuman OA. Cellular response to hypoxia involves signaling via Smad proteins. *Blood.* 2003;101(6):2253-2260.
24. Faleiro R, Karunarathne DS, Horne-Debets JM, Wykes M. The Contribution of Co-signaling Pathways to Anti-malarial T Cell Immunity. *Front Immunol.* 2018;9:2926.
25. Rao A, Kumar MK, Joseph T, Bulusu G. Cerebral malaria: insights from host-parasite protein-protein interactions. *Malar J.* 2010;9:155.
26. Hu C, Tao L, Cao X, Chen L. The solute carrier transporters and the brain: Physiological and pharmacological implications. *Asian J Pharm Sci.* 2020;15(2):131-144.
27. Scorziello A, Savoia C, Sisalli MJ, et al. NCX3 regulates mitochondrial Ca(2+) handling through the AKAP121-anchored signaling complex and prevents hypoxia-induced neuronal death. *J Cell Sci.* 2013;126(Pt 24):5566-5577.
28. Karakashev SV, Reginato MJ. Hypoxia/HIF1alpha induces lapatinib resistance in ERBB2-positive breast cancer cells via regulation of DUSP2. *Oncotarget.* 2015;6(4):1967-1980.
29. Whelan KA, Schwab LP, Karakashev SV, et al. The oncogene HER2/neu (ERBB2) requires the hypoxia-inducible factor HIF-1 for mammary tumor growth and anoikis resistance. *J Biol Chem.* 2013;288(22):15865-15877.
30. Fischer M, Reuter J, Gerich FJ, et al. Enhanced hypoxia susceptibility in hippocampal slices from a mouse model of rett syndrome. *J Neurophysiol.* 2009;101(2):1016-1032.
31. Kron M, Zimmermann JL, Dutschmann M, Funke F, Muller M. Altered responses of MeCP2-deficient mouse brain stem to severe hypoxia. *J Neurophysiol.* 2011;105(6):3067-3079.
32. Sato Y, Inoue M, Yoshizawa T, Yamagata K. Moderate hypoxia induces beta-cell dysfunction with HIF-1-independent gene expression changes. *PLoS One.* 2014;9(12):e114868.
33. Bencokova Z, Kaufmann MR, Pires IM, Lecane PS, Giaccia AJ, Hammond EM. ATM activation and signaling under hypoxic conditions. *Mol Cell Biol.* 2009;29(2):526-537.
34. Olcina MM, Grand RJ, Hammond EM. ATM activation in hypoxia - causes and consequences. *Mol Cell Oncol.* 2014;1(1):e29903.
35. D'Ignazio L, Rocha S. Hypoxia Induced NF-kappaB. *Cells.* 2016;5(1).
36. Taylor CT, Cummins EP. The role of NF-kappaB in hypoxia-induced gene expression. *Ann N Y Acad Sci.* 2009;1177:178-184.
37. Tripathi AK, Sha W, Shulaev V, Stins MF, Sullivan DJ, Jr. Plasmodium falciparum-infected erythrocytes induce NF-kappaB regulated inflammatory pathways in human cerebral endothelium. *Blood.* 2009;114(19):4243-4252.
38. Chen M, Shen C, Zhang Y, Shu H. MicroRNA-150 attenuates hypoxia-induced excessive proliferation and migration of pulmonary arterial smooth muscle cells through reducing HIF-1alpha expression. *Biomed Pharmacother.* 2017;93:861-868.

39. Sahu PK, Hoffmann A, Majhi M, et al. Brain Magnetic Resonance Imaging Reveals Different Courses of Disease in Pediatric and Adult Cerebral Malaria. *Clin Infect Dis.* 2020.
40. Soni R, Sharma D, Rai P, Sharma B, Bhatt TK. Signaling Strategies of Malaria Parasite for Its Survival, Proliferation, and Infection during Erythrocytic Stage. *Front Immunol.* 2017;8:349.
41. Eltahir EM, El Ghazali G, TM AE, IE AE, Elbashir MI, Giha HA. Raised plasma insulin level and homeostasis model assessment (HOMA) score in cerebral malaria: evidence for insulin resistance and marker of virulence. *Acta Biochim Pol.* 2010;57(4):513-520.
42. Cahayani WA, Norahmawati E, Budiarti N, Fitri LE. Increased CD11b and Hypoxia-Inducible Factors-1alpha Expressions in the Lung Tissue and Surfactant Protein-D Levels in Serum Are Related with Acute Lung Injury in Severe Malaria of C57BL/6 Mice. *Iran J Parasitol.* 2016;11(3):303-315.
43. Medana IM, Day NP, Roberts R, et al. Induction of the vascular endothelial growth factor pathway in the brain of adults with fatal falciparum malaria is a non-specific response to severe disease. *Histopathology.* 2010;57(2):282-294.
44. Ly A, Hansen DS. Development of B Cell Memory in Malaria. *Front Immunol.* 2019;10:559.
45. Donnelly S, Huston WM, Johnson M, et al. Targeting the master regulator mTOR: a new approach to prevent the neurological of consequences of parasitic infections? *Parasit Vectors.* 2017;10(1):581.
46. Ng S, March S, Galstian A, et al. Hypoxia promotes liver-stage malaria infection in primary human hepatocytes in vitro. *Dis Model Mech.* 2014;7(2):215-224.
47. Gupta H, Chaudhari S, Rai A, et al. Genetic and epigenetic changes in host ABCB1 influences malaria susceptibility to Plasmodium falciparum. *PLoS One.* 2017;12(4):e0175702.
48. Turner GD, Ly VC, Nguyen TH, et al. Systemic endothelial activation occurs in both mild and severe malaria. Correlating dermal microvascular endothelial cell phenotype and soluble cell adhesion molecules with disease severity. *Am J Pathol.* 1998;152(6):1477-1487.
